# Effectiveness of dietary interventions in individuals with diabetes for preventing and healing chronic wounds; a systematic review with meta‐analysis

**DOI:** 10.1111/dme.70100

**Published:** 2025-07-09

**Authors:** Hailey R. Donnelly, Clare E. Collins, Erin D. Clarke, Prudence I. Morrissey, Natalie Gilbertson‐Viljevac, Lucy Leigh, Peta E. Tehan

**Affiliations:** ^1^ School of Health Sciences College of Health, Medicine and Wellbeing, University of Newcastle Newcastle New South Wales Australia; ^2^ Food and Nutrition Research Program Hunter Medical Research Institute New Lambton Heights New South Wales Australia; ^3^ School of Medicine and Public Health College of Health, Medicine and Wellbeing, University of Newcastle Newcastle New South Wales Australia; ^4^ Hunter Medical Research Institute New Lambton Heights New South Wales Australia; ^5^ School of Clinical Sciences Faculty of Medicine, Nursing and Health Sciences, Monash University Melbourne Victoria Australia

**Keywords:** diabetes, diabetes‐related foot ulcer, diet, dietary, nutrition intervention, systematic review, wounds

## Abstract

**Background:**

Optimising the nutritional status of individuals with diabetes is essential to optimise glycaemic control, as well as to prevent and promote wound healing. A variety of nutrition interventions are available. This systematic review and meta‐analysis aimed to describe and synthesise the effectiveness of nutrition interventions for the prevention and management of chronic wounds in people with diabetes.

**Methods:**

Five databases and four clinical trial registries were searched for nutrition intervention studies. Included studies involved a nutrition intervention, such as personalised medical nutrition therapy, education and/or nutrient supplementation for those with diabetes and a chronic wound. Meta‐analysis was completed utilising mean wound size at follow‐up and non‐adjusted data where available. Quality was appraised using RoB 2 and ROBINS, and certainty of evidence was assessed using GRADE.

**Results:**

Twenty‐three studies were included; all included studies were in diabetes‐related foot ulcer populations. Meta‐analyses demonstrated nutrient supplements, including single and multi‐nutrient supplements, significantly reduced wound depth (MWD −0.200 [95% CI −0.364, −0.035], *p* = 0.0172), width (WMD −0.466 [95% CI −0.724, −0.208], *p* = 0.0004) and length (−0.443 [95% CI −0.841, −0.045], *p* = 0.0292), the quality of included studies was low and the certainty of evidence was very low. While substantial heterogeneity was detected (*I* ^2^ = 56%–68%), a random‐effects meta‐analysis was conducted to account for between‐study variability, providing an overall estimate while acknowledging differences in study characteristics. Two studies evaluating the effect of nutrition education demonstrated significant wound size reduction (*n* = 1) and proportion of people healed (*n* = 1).

**Conclusion:**

There is low‐quality evidence that nutrient supplementation improves wound healing. Nutrition education may have a role in enhancing wound size reduction. Further studies are needed using robust methodologies to comprehensively determine the effectiveness of nutrition interventions for wound healing.

**What's new?**

- No systematic reviews have explored all available dietary interventions for wound healing in individuals with diabetes.
- We found a lack of consistency in the measurement of wound healing and a lack of assessment of diet quality.
- There is low‐quality evidence that nutrition supplementation improves wound healing, specifically wound depth, width and length reduction.
- There was a lack of studies on dietary interventions in wound prevention and in wound types other than diabetes‐related foot ulceration.
- This study highlights the need for a standardised methodology and outcomes for trials exploring dietary interventions in individuals living with chronic wounds and diabetes.

## 1 INTRODUCTION

Chronic wounds are hard‐to‐heal wounds that do not achieve timely healing despite optimal care in 4–6 weeks.^1^, ^2^ Chronic wounds have a substantial impact on an affected individual's quality of life, but also present a substantial financial burden to the healthcare system.^3^ Diabetes is one of the primary contributors to impaired or delayed wound healing.^4^, ^5^ There are a combination of underlying pathologies that complicate wound healing processes in diabetes, including neuropathic, vascular, immune and biochemical factors.^6^, ^7^ Optimal wound healing requires adequate intake of macro‐ and micronutrients.^8^, ^9^ Previous cross‐sectional studies have demonstrated that individuals with diabetes and chronic wounds demonstrate poor diet quality, micronutrient deficiencies, with a high prevalence of malnutrition.^10^, ^11^, ^12^, ^13^, ^14^, ^15^, ^16^, ^17^ Many studies have investigated the relationship between nutrition and wound healing, particularly in pressure injuries (PI).^9^, ^10^, ^18^ Specific nutrients have also been associated with delayed healing, resulting in multiple studies aiming to supplement specific nutrients to improve healing outcomes.^9^, ^19^, ^20^, ^21^

Adequate nutritional status also plays an important role in the prevention of wounds and their recurrence.^22^ A previous quasi‐experimental study demonstrated that vitamin E supplementation was associated with reduced incidence of DFU.^23^ However, there is limited high‐level evidence investigating the effectiveness of specific nutrients in the prevention of wounds, with most research focused on wound healing. This is despite the important role that many nutrients play in maintaining skin integrity, critical to wound prevention as well as tissue repair and fighting infection.^24^, ^25^ Protein is required for collagen synthesis, activation of the immune response and aids in skin integrity.^25^ Vitamins A, C and E play roles in the maintenance of skin homeostasis and protect the skin from oxidative and UV irradiation‐mediated damage,^24^ all essential in collagen production.^25^ Vitamin D plays a role in immunomodulation, inflammation, angiogenesis and wound healing,^24^ and zinc activates antibodies and lymphocytes in the immune response, which subsequently aids in the prevention of infection.^24^ Whilst targeting specific nutrients is a logical solution to potentially improving wound healing, nutritional interventions can also include behavioural modification to improve dietary intake using medical nutrition therapy (MNT).^26^ The potential benefits of this extend beyond wound prevention and healing to improving other important risk factors such as glycaemic control, lipid management and hypertension.^26^

MNT is a dietitian‐led, holistic, evidence‐based approach which presents an opportunity for individuals with diabetes to make long‐term behaviour change with long‐term positive health effects.^26^ MNT has been demonstrated to be effective in reducing HbA1c within 3–6 months by up to 1.9% and 2% in those with type 1 and type 2 diabetes, with ongoing MNT supporting in maintaining lower HbA1c.^27^ In people with diabetes, MNT has been shown to reduce dietary intake of added sugars and refined grains, increase wholegrains and dietary fibre and manage carbohydrates.^26^ A randomised controlled trial also demonstrated that a 12‐week diabetes nutrition education group session intervention led by a nutritionist can significantly reduce HbA1c compared with the control group.^28^ Furthermore, a cluster randomised controlled trial conducted in individuals living with type 2 diabetes in China using dietitian‐led group education in addition to individualised nutrition counselling sessions demonstrated a significant improvement in HbA1c at 1 year compared with controls.^29^

Whilst fats and carbohydrates contribute to the inflammatory response, cell functioning and collagen deposition in the proliferative phase of wound healing,^8^ diabetes populations have very specific dietary considerations including glucose control, fats and carbohydrates.^30^ Individuals with diabetes and a chronic wound, therefore, present even more complex challenges in optimising dietary intake to address any nutrient deficiencies to optimise wound healing. Previous systematic reviews and meta‐analyses exploring diet and wound healing have focused on the effectiveness of specific nutrition supplements for different wound aetiologies (VLU, DFU, PI),^10^, ^31^, ^32^, ^33^, ^34^, ^35^, ^36^, ^37^, ^38^, ^39^ none have explored the range of available dietary interventions (behavioural and/or supplementation) on wound prevention and/or chronic wound management, and none have focused on these interventions for individuals with diabetes.

### 1.1 Objectives

The aims of this systematic review and meta‐analysis were:

1. To determine the effectiveness of dietary interventions in people with diabetes, primarily for chronic wound prevention and/or wound healing,
2. To describe the characteristics of the dietary interventions,
3. To determine the quality of the evidence available,
4. To aggregate results in a meta‐analysis, if possible.

## 2 METHODS

This systematic review with meta‐analysis has been reported in accordance with the Preferred Reporting Items for Systematic reviews and Meta‐Analysis checklist (PRISMA).^40^ The protocol has been previously published^41^ and the review registered with Prospero (CRD42023434910).

### 2.1 Eligibility criteria

The PICOS (Population, Intervention, Comparison, Outcomes and Study design) criteria were utilised to guide eligibility criteria (Table 1). Further details were published elsewhere.^41^ In variance to the protocol, studies that did not report WIfI,^42^ Texas^43^ or Wagner's^44^ wound classification system were included. Our protocol failed to recognise older studies that may have preceded these systems, and also the potential inclusion of wound types other than DFU. Furthermore, the primary outcome was wound healing, not severity, so it was deemed appropriate to include studies that did not necessarily report a grading system. This decision was made prior to the screening of abstracts. When a cohort included people with and without diabetes, the population with diabetes needed to be able to be analysed separately to be eligible for inclusion.

**TABLE 1:** PICOS eligibility criteria.

Participants/setting	Inclusion: Individuals with type 1 or 2 diabetes with a wound, of any age, sex and ethnicity, with any co‐morbidities In any healthcare setting (i.e., hospital, community, residential aged care) Wounds: Diabetes‐related foot ulcerations Pressure ulcerations/injuries Arterial/ischaemic ulcers Venous leg ulcerations Mixed leg ulcerations Wound classification systems including but not limited to: Texas Wound Classification System^43^ WIFi Classification System^42^ Wagner's Classification.^44^
Intervention(s)	Inclusion: Dietary interventions such as: Supplementation (oral, enteral, or parenteral) of any type (food pattern, macronutrient, vitamin, mineral, or multi‐nutrient), dose, mode, duration; any special diet Nutrition education Dietitian intervention and/or nutrition counselling
Comparator(s)	Inclusion: The control group did not receive a dietary intervention OR Usual care/diet, OR Two or more types of dietary interventions for wound prevention/treatment
Outcome(s)	Inclusion: Primary outcomes: Incidence of ulcers/wounds Wound healing indicators, including time to healing, change in healing rate, wound area and depth, total number of wounds, or proportion healed at study completion Secondary outcomes: Food and nutrient intakes and/or diet quality, food behaviour HbA1c, fasting blood glucose, lipids, inflammatory markers (e.g., CRP, IL6, TNF‐alpha) Acceptability measures including satisfaction with intervention, cost and attrition Quality of life Admission and length of stay – if in hospital Surgical interventions including lower extremity amputation
Study design	Inclusion: Randomised control trials Pseudo‐randomised control trials Quasi‐experimental studies Pre‐ and post‐studies of any time frame

### 2.2 Information sources

Five databases were searched: Medline, Embase, CINAHL, Scopus and Cochrane Library from inception up until May 2024. Reference lists of included studies were also screened for any additional relevant studies. Authors were contacted for additional information where necessary. Clinical trials databases (Europe, Australia and New Zealand, World Health Organisation and the USA trials database) were also searched for applicable registered studies up until May 2024. Undertaking the trial database searches was used to confirm that all completed studies were included in the current systematic review, with the full text of relevant trials also included. Authors of any trials that had not been published were contacted to determine the progress of the study. If a study was completed and published and met eligibility criteria, it was included in the systematic review.

### 2.3 Search strategy

The research team developed the search strategy in consultation with a health and medical research librarian. To determine if the search strategy was efficient in identifying relevant articles, an initial search was conducted on Medline. The Medline search strategy is presented in the published protocol,^41^ whilst the other search strategies can be found in Supplementary Material S1. Limits were placed on the search such as limited to human subjects and studies published in English.^41^

### 2.4 Study records

#### 2.4.1 Data management

Covidence systematic review software® (Veritas Health Innovation, Melbourne, Australia) was utilised for storage of eligible studies and to facilitate title and abstract screening, full text screening and data extraction.

#### 2.4.2 Selection process

Each abstract and full text was independently screened by two members of the research team. Conflicts were resolved by another researcher or through discussion. If studies were determined ineligible and subsequently excluded during full‐text screening, a reason for exclusion was recorded. The level of agreement between reviewers was determined utilising Cohen's Kappa,^45^ provided by Covidence systematic review software®, and reported in the results section.

#### 2.4.3 Data collection process

Data was extracted from included articles by an independent researcher, and a uniform data extraction criterion was developed by the research team. The data extraction criterion was pilot tested with five studies randomly selected. An independent second reviewer checked 10% of the data extraction.

### 2.5 Risk of bias in individual studies

The Rob‐2 and ROBINS quality appraisal tools were utilised to evaluate the risk of bias of individual studies by two independent reviewers (HD and PM).^46^, ^47^ A third reviewer addressed any disagreements that arose (EC).

### 2.6 Data synthesis

Data from included studies were characterised by intervention type and outcome variables and synthesised based on study aims. Where adjusted and unadjusted results were available for the narrative results, we reported the results for the adjusted models. Between‐group difference at follow‐up was reported for all primary and secondary outcomes. If between‐group differences were reported in the included paper, they were directly used (and the associated reported *p*‐values were used). If between‐group differences at follow‐up were not reported in the study publication, they were calculated manually using individual group means and standard deviations, and a *p*‐value and confidence intervals were estimated using a *t*‐test. Where standard deviations were not available, they were back‐calculated from confidence intervals or the inter‐quartile range (IQR). Similarly, for count outcomes, when the between‐group difference and *p*‐value were reported, these were used directly. When these were not available, individual group *N*(%) were used to calculate the difference in proportions, and the *p*‐value and confidence intervals were obtained via the difference in proportions test. For the results text, mean and standard deviation (SD) or median and IQR were reported, depending on the distribution of the data.^48^

For the meta‐analysis, data were extracted primarily from RCTs at the 12‐week follow‐up time point for wound depth, width, length and the proportion of participants healed. Analyses were performed using STATA for Windows (version BE 17.0). For continuous outcomes, sample size (N), means and SD per study arm were extracted. For categorical outcomes, the number of events and non‐events was extracted for each study arm. Where SDs were not reported, confidence intervals were used to be calculated.^49^ In instances where the mean at follow‐up was not reported, mean differences within groups were used.^48^ For clustered studies, the design effect was calculated, and the sample size was adjusted accordingly.^50^ Where data were available, the meta‐analysis used unadjusted data; adjusted data were utilised, and a sensitivity analysis was conducted to assess the incremental impact. Forest plots were created for each outcome of interest (wound depth, width and length and proportion of people healed).

A number of sensitivity analyses were performed to test the robustness of the study findings. Firstly, for each outcome, non‐randomised studies were excluded to explore the impact of the non‐randomised studies on the overall effect. Further, for outcomes where a mix of adjusted and non‐adjusted effect sizes were reported, sensitivity analyses excluding the adjusted effect sizes were performed. Funnel plots were used to assess potential bias and skewness related to publication bias, with trim and fill methods used to estimate the impact of this bias on the results. Finally, to explore whether follow‐up duration contributed to heterogeneity, we conducted meta‐regressions comparing studies with and without the 12‐week time point. A random‐effects model was used due to the high heterogeneity between studies.

### 2.7 Confidence in cumulative evidence

The Grading of Recommendations Assessment, Development and Evaluation (GRADE) system assessed the certainty and strength of the body of evidence.^51^ This was applied by two authors independently (HD, PT).

## 3 RESULTS

### 3.1 Summary of search

**FIGURE 1:**
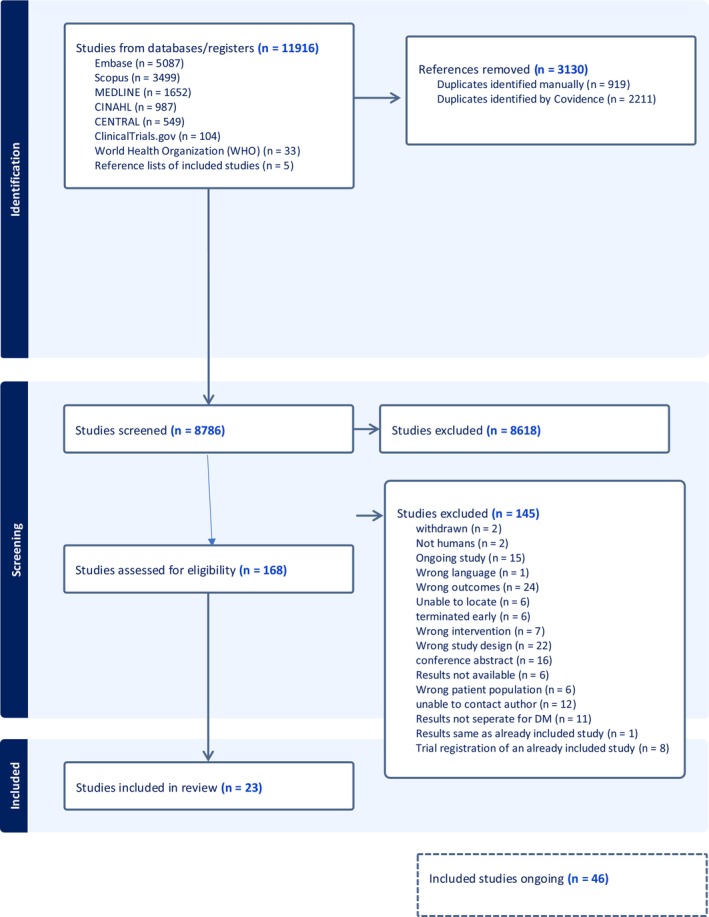
PRISMA flow diagram.

Results of the search are presented in the PRISMA flow diagram (Figure 1). Twenty‐three studies met the inclusion criteria.

Trial databases identified 37 relevant trials; 11 had been completed, with two not yet having results published, 17 were ongoing trials, six were terminated, two were withdrawn and one prematurely ended. There were no additional completed published studies that had not been identified from the original search strategy (Supplementary Table S1).

There was a moderate level of agreement between the reviewers in the title and abstract screening (Cohen's Kappa: range 0.41 to 0.52), and a substantial level of agreement for full text screening (Cohen's Kappa: range 0.68 to 1).^45^

### 3.2 Characteristics of included studies

Of the 23 included studies, seventeen were randomised controlled trials (RCT)^21,52–67^ (Supplementary Table S2). All included studies were in DFU populations. The number of participants ranged from 16^57^ to 308,^23^ with a median of 60 ((IQR) 50), across a range of countries (Supplementary Table S2). The median age (IQR) for the intervention group and control group was 58.8 years (IQR 5.7) and 59.3 years (IQR 2.3), respectively.

Across the included studies, 21 included individuals with diabetes only; two studies included both individuals with and without diabetes, where data were available for the diabetes participants.^57,68^ Ten studies reported diabetes duration, with the mean/median duration ranging from 0 to 51 years. Ten studies did not report what type of diabetes included participants had.^21,52,56,59–62,64,65,69^ Seven studies included participants with both type 1 and type 2 diabetes, and six studies included those with type 2 diabetes only (Supplementary Table S2).^55,63,66,68,70,71^

For wound classification systems, 14 studies utilised Meggitt–Wagner's criteria,^21,52,54,56,59–68^ two utilised the University of Texas^53,55^ and seven did not report a wound classification system. No studies utilised the WiFI classification system. Of the 14 studies that reported Meggitt–Wagner's criteria, five studies included only participants with a grade 1 or 2 ulcer,^54,56,63,66,67^ one included only grade 2 ulcers,^68^ seven included grade 3 ulcers only,^21,52,60–62,64,65^ and one included grade 2 or 3 ulcers.^59^ One RCT included participants with at least one University of Texas grade 1A foot ulcer,^53^ while the other study included those with at least one University of Texas grade 1A or 2A foot ulcer.^55^

A history of amputation was reported in one study only,^67^ and previous ulceration was reported in one study only.^53^

Thirteen studies performed power calculations and reported that they were powered to detect change. However, it is important to note that seven studies based their power calculation on HOMA‐IR, despite the primary outcome being reported as wound healing.^21,52,60–62,64,65^ Ten studies did not report whether the study sample size target was informed by a power calculation (Table 2).^23,54–56,59,68–72^

**TABLE 2:** Summary of the included studies' primary aim and how each study assessed wound healing.

Reference, country	Study details							How wound healing was assessed								
	Primary outcomes						Study powered to detect change	Proportion healed	Wound reduction	Wound area/volume	Wound surface area	Wound length	Wound width	Wound depth	Time to healing	Other
	Wound healing	Metabolic Status^b^	Protein‐Energy Malnutrition	Feasibility of intervention	Rehabilitation and QOL^c^	Preventing the development and progression of diabetes‐related complications										
Afzali 2019, Iran^a^	✓	✓					✓					✓	✓	✓		
Armstrong 2014, USA, Europe and Taiwan^a^	✓						✓	✓	✓	✓						
Bashmakov 2014, Egypt^a^	✓						NR	✓			✓	✓	✓	✓		✓
Basiri 2020, USA^a^	✓						NR	✓	✓							
Bosede 2012, Nigeria	✓	✓					NR									✓
Das 2022, India	✓						NR									✓
Eneroth 2004, Sweden^a^	✓		✓				NR	✓								
Gunton 2021, Australia^a^	✓						✓		✓						✓	
Halschou‐Jensen 2021, Denmark^a^	✓						✓	✓	✓	✓						
Jain 2012, India						✓	NR									✓
Kamble 2020, India^a^	✓	✓					NR				✓					
Mohseni 2018, Iran^a^	✓	✓					✓					✓	✓	✓		
Mokhtari 2020, Iran^a^	✓	✓					✓					✓	✓	✓		
Momen‐Heravi 2017, Iran^a^	✓	✓					✓					✓	✓	✓		
Mozaffari‐Khosravi 2016, Iran^a^	✓						✓			✓						
Rangabashyam 2020, India	✓						NR					✓	✓	✓		
Razzaghi 2017, Iran^a^	✓	✓					✓					✓	✓	✓		
Razzaghi 2018, Iran^a^	✓	✓					✓					✓	✓	✓		
Soleimani 2017, Iran^a^	✓	✓					✓					✓	✓	✓		
Sung 2021, Australia	✓			✓			NR	✓		✓						
Yanes‐Quesada 2021, Cuba^a^	✓						✓	✓								
Yang 2023, China					✓		NR	✓								
Yarahmadi 2021, Iran^a^	✓	✓					✓	✓		✓		✓	✓			

^a^ Randomised controlled trials.

^b^ Metabolic status (Glycaemic control, lipids and/or oxidative and inflammatory markers).

^c^ QOL = quality of life.

### 3.3 Interventions

All studies included dietary interventions for treatment only of DFU (*n* = 23), whereas one included both prevention and treatment of DFU.^23^ Twenty studies evaluated nutrient supplementation alone (Table 3). Of the remaining three studies, one included a dietitian as part of a multidisciplinary team in addition to standard podiatry care,^72^ one utilised nutrition education as a standalone intervention,^69^ and the other one involved nutrition education in combination with nutrient supplementation (Supplementary Table S2).^55^

**TABLE 3:** Summary of included studies nutrition intervention utilised.

Reference, country	Vit C^a^	Vit D^a^	Vit E^a^	Se^b^	Zn^c^	Mg^d^	Probiotic	Nano‐curcumin	Omega 3	HMB^e^ + glutamine + arginine	Amino acids^f^	t‐RSV^g^	Nutricia Fortimel ONS^h^	Nestle Blood Glucose Control ONS^h^	Diamel	Nurse‐led early nutrition intervention	Dietitian
Afzali 2019, Iran^i^			✓			✓											
Armstrong 2014, USA, Europe and Taiwan^i^										✓							
Bashmakov 2014, Egypt^i^												✓					
Basiri 2020, USA^i^														✓			✓
Bosede 2012, Nigeria	✓		✓	✓													
Das 2022, India											✓						
Eneroth 2004, Sweden^i^													✓				
Gunton 2021, Australia^i^	✓																
Halschou‐Jensen 2021, Denmark^i^		✓															
Jain 2012, India			✓														
Kamble 2020, India^i^		✓															
Mohseni 2018, Iran^i^							✓										
Mokhtari 2020, Iran^i^								✓									
Momen‐Heravi 2017, Iran^i^					✓												
Mozaffari‐Khosravi 2016, Iran^i^		✓															
Rangabashyam 2020, India		✓															
Razzaghi 2017, Iran^i^		✓															
Razzaghi 2018, Iran^i^						✓											
Soleimani 2017, Iran^i^									✓								
Sung 2021, Australia																	✓
Yanes‐Quesada 2021, Cuba^i^															✓		
Yang 2023, China																✓	
Yarahmadi 2021, Iran^i^	✓		✓														

^a^ Vit = vitamin.

^b^ Se = selenium.

^c^ Zn = zinc.

^d^ Mg = magnesium.

^e^ HMB = beta‐hydroxy‐beta‐methylbutyrate.

^f^ Amino acids (does not specify).

^g^ t‐RSV = trans‐resveratrol.

^h^ ONS = Oral nutritional supplement.

^i^ Randomised controlled trials.

**FIGURE 2:**
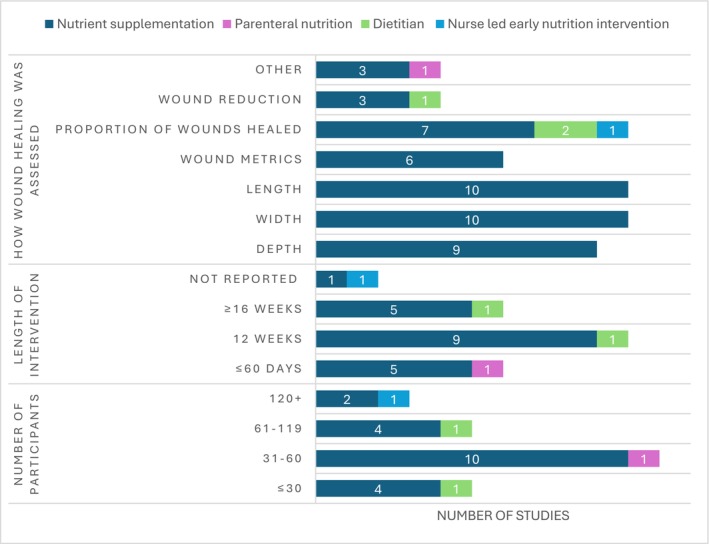
Study characteristics according to the type of nutrition intervention provided.

Twelve studies evaluated the effectiveness of oral single‐nutrient supplements (Figure 2). Multi‐nutrient supplements were used in eight studies,^52,53,55,56,66–68,70^ none of which used the same nutrient combinations (Table 3). One study tested the intravenous administration of amino acids; however, it did not specify which amino acids the supplement contained.^70^ The majority (*n* = 12) of interventions were oral supplements that were compared with placebo supplement control groups.^21,52–54,56,57,60–62,64,65,67^ Study duration varied, ranging from 4 weeks to 1 year, or until wound closure or surgical intervention. The mean retention rate for the studies that reported retention rates (*n* = 16) was 88.81%, with seven not reporting this data.^23,59,68–72^

### 3.4 Wound healing outcomes

Studies primarily measured wound width (*n* = 10),^21,52,54,60–62,64,65,67,71^ length (*n* = 10)^21,52,54,60–62,64,65,67,71^ and depth (*n* = 9)^21,52,54,60–62,64,65,71^ as indicators of wound healing (Supplementary Table S3).

Across the five studies investigating reduction in wound size, three reported a statistically significant result favouring the nutrition intervention (vitamin C vs. placebo for 56 days,^57^ high‐dose vitamin D (intervention) vs. low‐dose vitamin D for up to 48 weeks^58^ and the other a multi‐nutrient supplement + dietitian vs. standard care for up to 12 weeks or until wound closure^55^); two studies found no significant differences between the nutrition intervention and control group (MDT vs. standard care over 6 months^72^ and HMB + glutamine + arginine vs. placebo for 16 weeks^53^).

Of the nine included studies reporting the proportion of people healed, four studies (High‐dose vs. low‐dose vitamin D for up to 48 weeks,^58^ vitamin C + E vs. placebo for 8 weeks,^67^ multi‐nutrient supplement for 1 year,^66^ and an early nursing nutrition intervention with duration not reported^69^) demonstrated significant results favouring the intervention and five reported no significant difference (HMB + glutamine + arginine vs. placebo for 16 weeks,^53^ trans‐resveratrol vs. placebo for 60 days,^54^ a multi‐nutrient supplement + dietitian vs. standard care for up to 12 weeks or until wound closure,^55^ a multi‐nutrient supplement vs. placebo for 6 months^56^ and MDT vs. standard care over 6 months^72^).

Six studies recorded wound metrics (wound area, volume, surface area) to determine wound healing; three reported significant effects favouring the intervention (two vitamin D studies,^58,59^ one high‐dose versus low‐dose vitamin D for up to 48 weeks^58^ and one vitamin D versus placebo for 12 weeks,^59^ and a vitamin C + E trial vs. placebo for 8 weeks^67^), with two noting non‐significant results (HMB + glutamine + arginine vs. placebo for 16 weeks^53^ and a vitamin D intramuscular injection study comparing high‐dose versus low‐dosevitamin D for 4 weeks^63^). One RCT investigating trans‐resveratrol vs. placebo for 60 days reported a significant reduction in wound size, favouring the intervention at 4 weeks; however, it found no significant difference at 4 weeks for wound area and no significant difference at 8 weeks for wound size or area.^54^ It is of note that the supplemental vitamin D studies reporting statistical significance between groups for wound metrics were of longer duration (48 weeks^58^ and 12 weeks^59^) than the short‐duration vitamin D study that found a non‐significant result (4 weeks^63^).

Seven studies, all with a duration of 12 weeks, had statistically significant between‐group differences favouring the intervention, with statistically significant improvements in wound width and length (vitamin E and Magnesium,^52^ probiotic,^60^ zinc,^62^ vitamin D,^21^ magnesium,^64^ omega‐3^65^ and vitamin C + E^67^). Of these, six studies reported a significant improvement in wound depth (vitamin E and Magnesium,^52^ probiotic^60^, zinc,^62^ vitamin D,^21^ magnesium^64^ and omega‐3^65^). However, one RCT investigating trans‐resveratrol versus placebo for 60 days reported a significant reduction in wound depth, favouring the intervention at 4 weeks; however, it found no significant differences at week 8 for wound depth, and no significant differences at any time point for wound width or length.^54^ Moreover, one quasi‐experimental study exploring vitamin D versus control for 6 weeks reported a significant reduction in wound width, favouring the intervention; it found no significant reductions in wound length or depth.^71^ Nano‐curcumin supplementation made no difference to wound outcomes.^61^

It should be noted that two studies used wound assessment tools to measure wound healing outcomes.^68,70^ One three‐arm quasi‐experimental study found statistical significance favouring the intervention using the ABDEFS (Aetiology, Base, Discharge, Edge, Floor, Size) Tool,^68^ and the other reported statistically significant results favouring the intervention when using the Southampton Scoring System and the Asepsis Wound Scoring System after 15 days of intravenous amino acids.^70^

One RCT investigated time to healing in days and noted a significantly faster median time to 50% ulcer healing in the intervention group receiving 500 mg of oral vitamin C in a slow‐release capsule compared with placebo (*p* = 0.028).^57^ One study reported a significant improvement in the incidence of foot ulcers, favouring the intervention group receiving vitamin E supplementation compared with standard care at the end of 24 months.^23^

### 3.5 Secondary outcomes

Secondary outcomes reported are presented in Supplementary Tables S4–S8.

### 3.6 Dietary intake (*n* = 7)

Measurement of dietary intake was varied, with six studies using 3‐day food records^21,52,60,62,64,65^ and one study using 24‐hour recalls to assess dietary intake (Supplementary Table S4).^55^

### 3.7 Nutritional status (*n* = 1)

One case–control study exploring the impact of an early nursing nutrition intervention evaluated participants' nutritional status utilising the nutrition risk screening tool (NRS 2002),^73^ which found a significant reduction in nutrition risk at the end of the study, favouring the intervention group (Supplementary Table S4).^69^

### 3.8 Glycaemic control (*n* = 17)

Out of 17 studies reporting differences in glycaemic control between groups at the end of the intervention, five found statistically greater reductions in HbA1c and fasting blood glucose in the intervention groups compared with placebo at the end of the 12‐week trial (vitamin E + magnesium,^52^ probiotic,^60^ zinc,^62^ vitamin D^21^ and omega‐3^65^), whilst three reported non‐significant differences at the end of the intervention compared with placebo (magnesium for 12 weeks,^64^ vitamin C + E for 8 weeks^67^ and vitamin C + E + selenium for 16 weeks^68^) (Supplementary Table S5). One RCT testing nano‐curcumin found a significantly greater reduction in the intervention group compared with placebo for fasting blood glucose post‐intervention; however, differences in HbA1c between groups were not significant.^61^ Another study reported a significantly greater reduction in HbA1c in the vitamin D supplement group compared with standard care, who had an increase in HbA1c after 12 weeks,^59^ while another RCT investigating high‐dose vitamin D versus low‐dose vitamin D supplementation for up to 48 weeks reported no significant differences between groups.^58^ One study exploring vitamin D intramuscular injections reported a significantly greater reduction in fasting blood glucose among the group receiving a once‐off dose of 300,000 IU of vitamin D compared with the group receiving a once‐off dose of 150,000 IU of vitamin D.^63^ One RCT providing a multi‐nutrient supplement intervention reported a significant reduction in HbA1c at the 1‐year follow‐up; however, blood glucose was non‐significant between groups.^66^ One quasi‐experimental study utilising vitamin E supplementation intervention reported non‐significant differences between groups for fasting plasma glucose; however, it reported significant reductions in post‐prandial glucose, favouring the intervention after 2 years.^23^ Another quasi‐experimental study using a vitamin D supplementation intervention also reported significant reductions in post‐prandial blood glucose levels favouring the intervention, and also found significant reductions in fasting blood glucose and HbA1c favouring the intervention after 6 weeks.^71^ One RCT investigating trans‐resveratrol versus placebo for 60 days reported no significant differences between groups for fasting plasma glucose.^54^

The dietitian, as part of the multidisciplinary team study reported a non‐significant difference between groups for HbA1c 5 months later.^72^ The dietitian and nutrient supplement study with a duration of up to 12 weeks or until wound closure did not report statistically or clinically significant results, and it was unable to be calculated as no SDs or confidence intervals were reported.^55^

### 3.9 Biochemistry (*n* = 21)

Most studies exploring the impact of nutrient supplementation did not find significant differences between groups for plasma lipid biomarkers post‐intervention, despite one study evaluating the impact of omega‐3 supplementation over 12 weeks (Supplementary Table S6).^65^

All studies that investigated micronutrient supplementation also reported biological outcomes for the respective nutrient, except for three RCTs testing the impact of vitamin E^23,52,67^ (Supplementary Table S5). Significant improvements were identified favouring the intervention in two RCTs testing supplements of magnesium,^52,64^ four studies (3 RCTs and 1 quasi‐experimental) used vitamin D,^21,59,63,71^ one quasi‐experimental study used vitamins C, E and selenium,^68^ and one RCT evaluated zinc supplements.^62^ Another RCT investigated high‐dose vitamin D versus low‐dose vitamin D supplementation for up to 48 weeks and reported significant improvements in vitamin D levels favouring the intervention (high dose) up to 24 weeks; however, it reported no significant differences between groups for 36 weeks and 48 weeks.^58^

### 3.10 Inflammatory markers (*n* = 12)

Inflammatory markers had mixed results, with only one RCT testing vitamin C and E supplementation noting significant reductions for all inflammatory markers, favouring the intervention group^67^. Seven studies identified significant reductions in high‐sensitivity C‐reactive protein favouring the intervention,^21,52,60,62–65^ while one study reported non‐significance (nanocurcumin).^61^ Moreover, one RCT (trans‐resveratrol) found no significant differences between groups for C‐reactive protein.^54^ Four studies reported significant reductions in erythrocyte sedimentation rate (vitamin D ONS,^21^ vitamin D intramuscular injection,^63^ zinc ONS,^62^ and vitamin E + magnesium ONS^52^), with three studies showing non‐significant differences between groups (probiotic,^60^ nano‐curcumin^61^ and magnesium^64^) (Supplementary Table S7).

### 3.11 Other biochemistry (*n* = 12)

Of the six studies exploring nitric oxide, two showed significant increases in nitric oxide in the intervention group (probiotic^60^ and zinc^62^), with four reporting non‐significant results (vitamin D,^21^ magnesium,^64^ omega‐3,^65^ and magnesium + vitamin E^52^). Total antioxidant capacity was investigated within eight studies, of which six reported significant improvements in total antioxidant capacity favouring the intervention group (probiotic,^60^ nano‐curcumin^61^, zinc,^62^ omega‐3,^65^ vitamin E + magnesium^52^ and vitamin C + E + selenium^68^); two reported non‐significant results (magnesium^64^ and vitamin D^21^). Furthermore, three studies (nano‐curcumin^61^, zinc^62^ and omega‐3^65^) reported significant improvements for total glutathione, favouring the intervention group; however, four studies reported non‐significance.^21,52,60,64^

Across the seven studies examining malondialdehyde, five studies noted significant differences favouring the intervention (probiotic,^60^ zinc,^62^ magnesium,^64^ vitamin D,^21^ and vitamin E + magnesium^52^), whilst two had non‐significant differences (nano‐curcumin^61^ and omega‐3^65^) (Supplementary Table S7).

### 3.12 Lower limb amputation (*n* = 4)

Of the four studies illustrating amputation rate, none found a significant difference between groups (Supplementary Table S8).^53,56–58^

### 3.13 Quality appraisal

**FIGURE 3:**
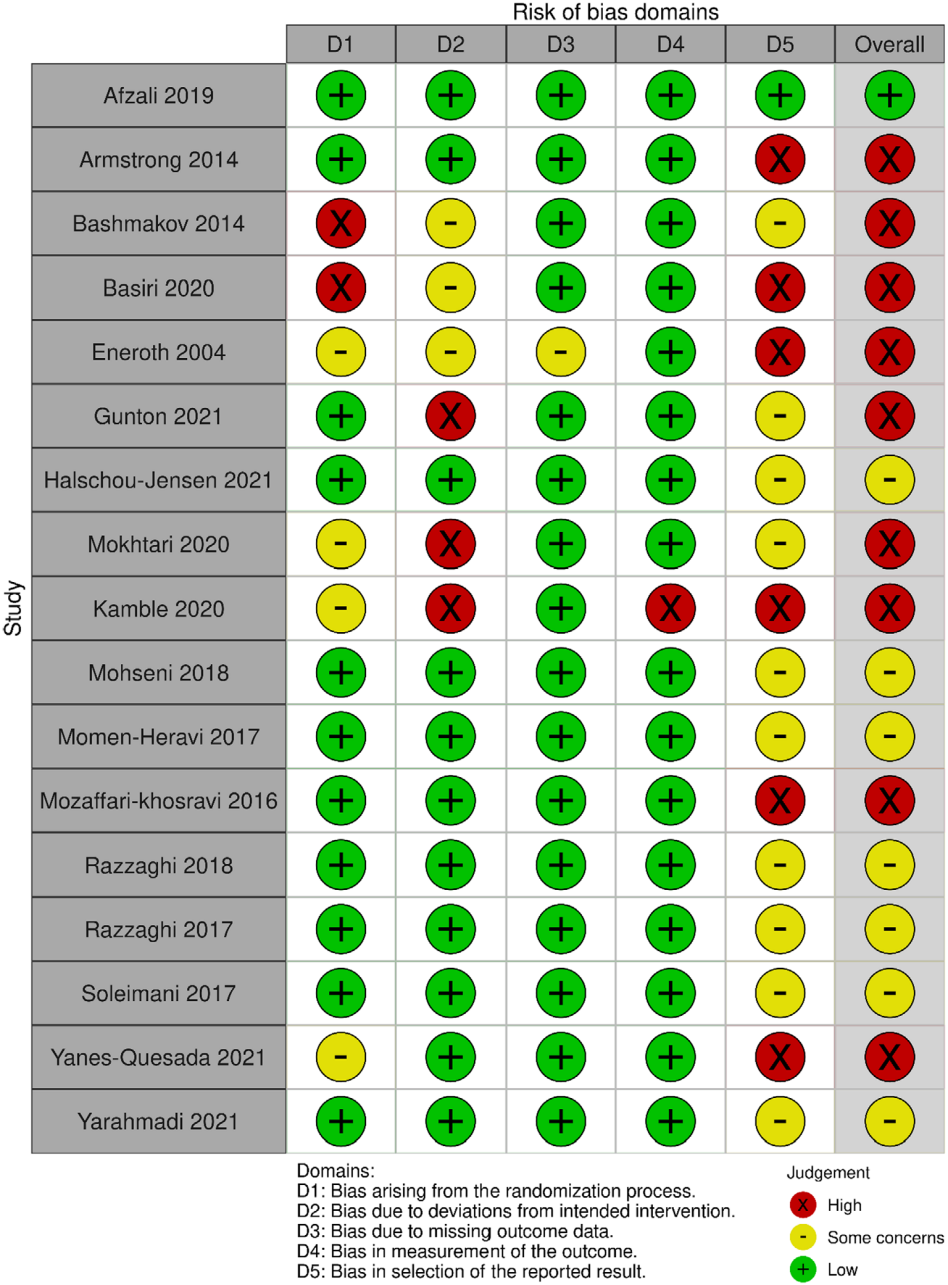
Risk of bias results for randomised controlled trials utilising ROB‐2 tool.

The ROB‐2 quality appraisal tool demonstrated that only one study was at low risk of bias,^52^ with seven studies at moderate risk of bias^21,58,60,62,64,65,67^ and nine studies that had a high risk of bias (Figure 3).^53–57,59,61,63,66^

**FIGURE 4:**
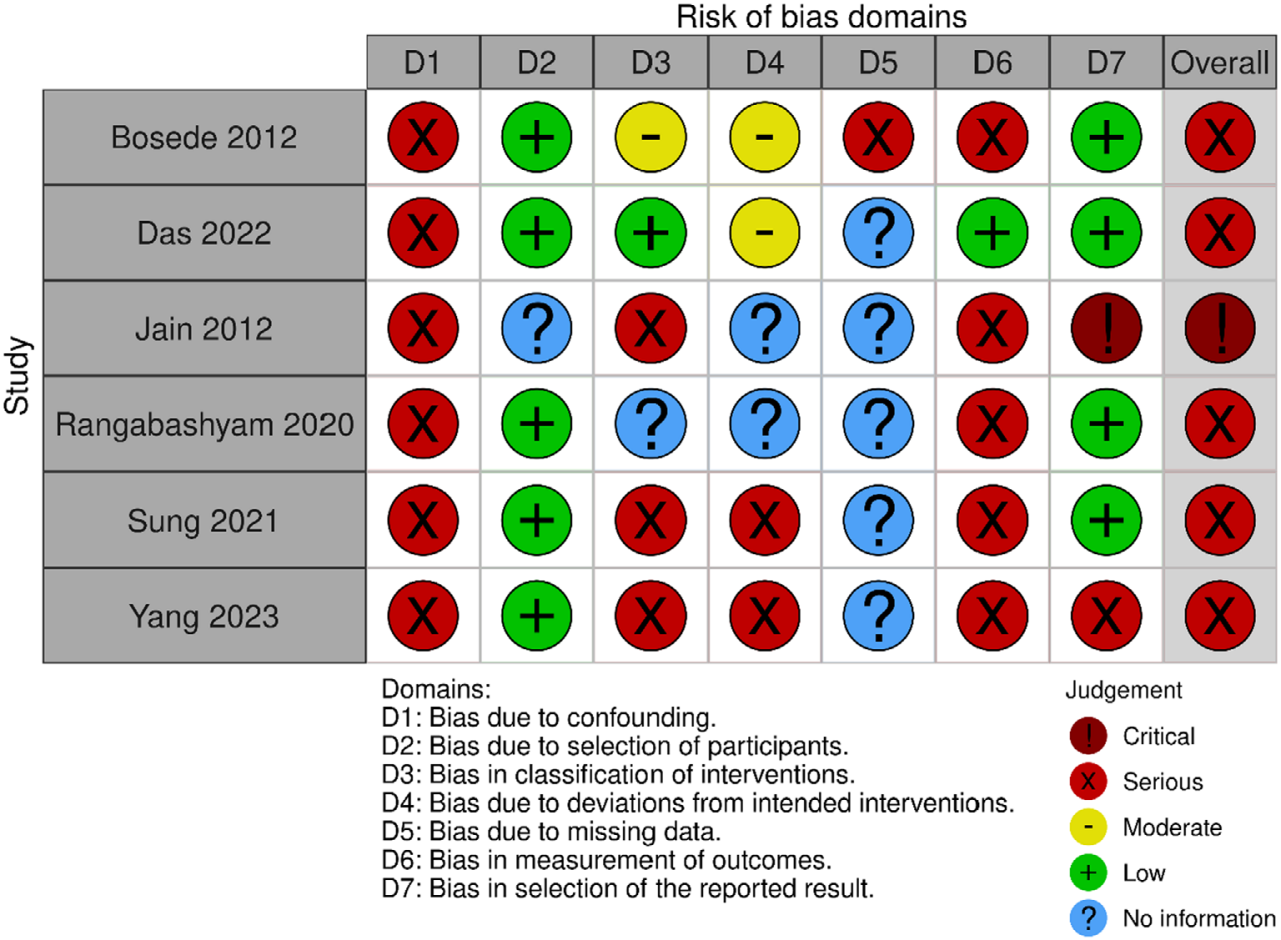
Risk of bias results for non‐randomised studies utilising ROBINS‐I tool^75^.

For non‐randomised studies, five were classified as serious risk of bias overall,^68–72^ with one considered to be at critical risk of bias using the ROBINS‐I tool (Figure 4).^23^

### 3.14 Meta‐analysis

Sixteen studies reported outcome data for wound depth, width and length, or proportion of people healed, and were eligible for inclusion in the meta‐analysis.^21,52–56,58,60–62,64–67,69,71^ The demographics reported for one quasi‐experimental study were not stratified per group; therefore, potential confounding was not able to be determined,^71^ and sensitivity analyses excluding it from the model were conducted for wound depth, width and length. Additional meta‐analysis information can be found in Supplementary Material S2.

#### 3.14.1 Wound depth

**FIGURE 5:**
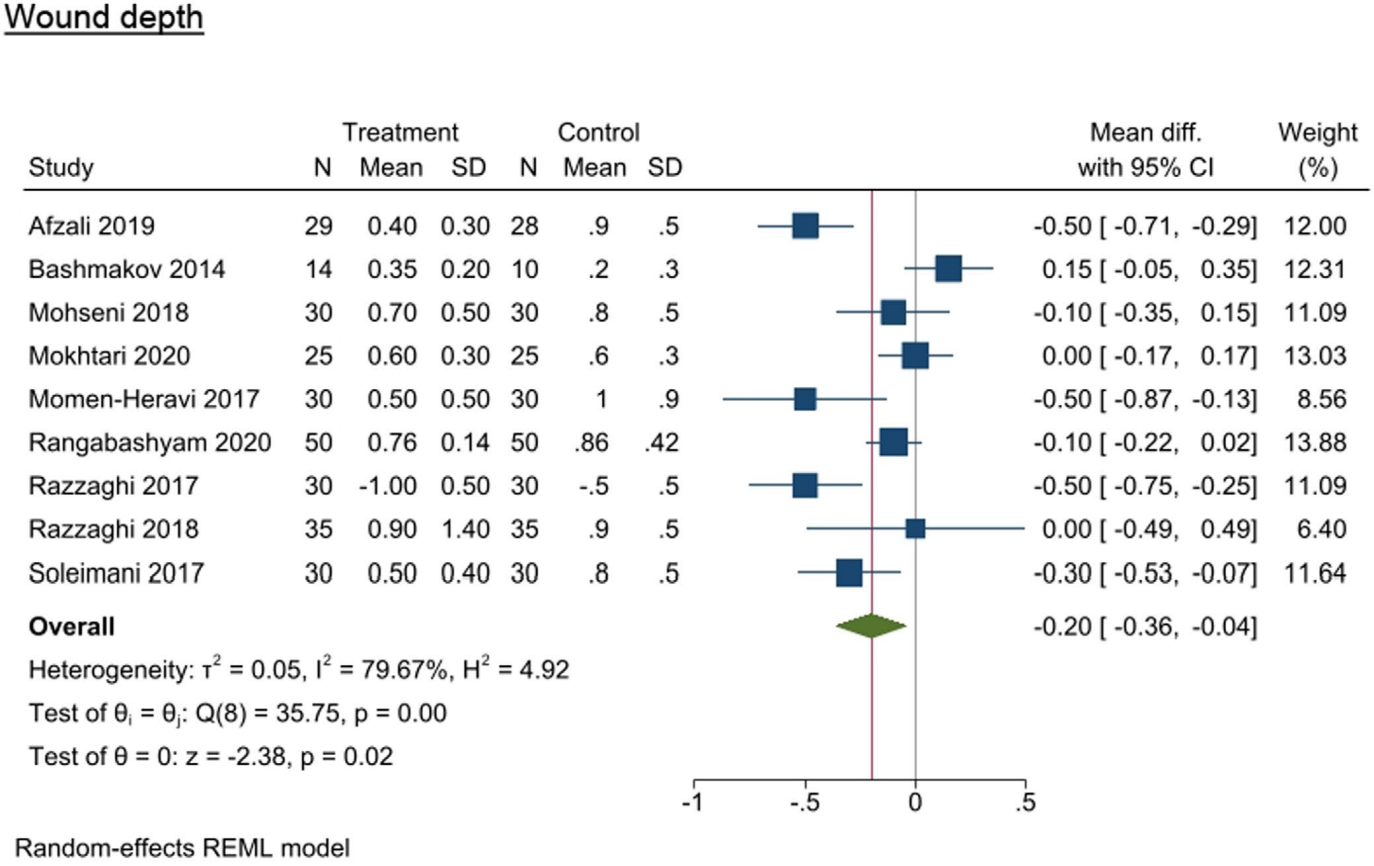
Nutrition intervention effect on wound depth (cm) relative to all control interventions.

Wound depth was assessed in nine studies (8 RCTs and 1 Quasi‐experimental study).^21,52,54,60–62,64,65,71^ There was a significant difference in wound depth across all studies, with the nutrition intervention group demonstrating a smaller wound depth (MWD −0.200 [95% CI −0.364, −0.035], *I* ^2^ = 79.67%, *p* = 0.0172) compared with the control group (Figure 5).

#### 3.14.2 Wound width

**FIGURE 6:**
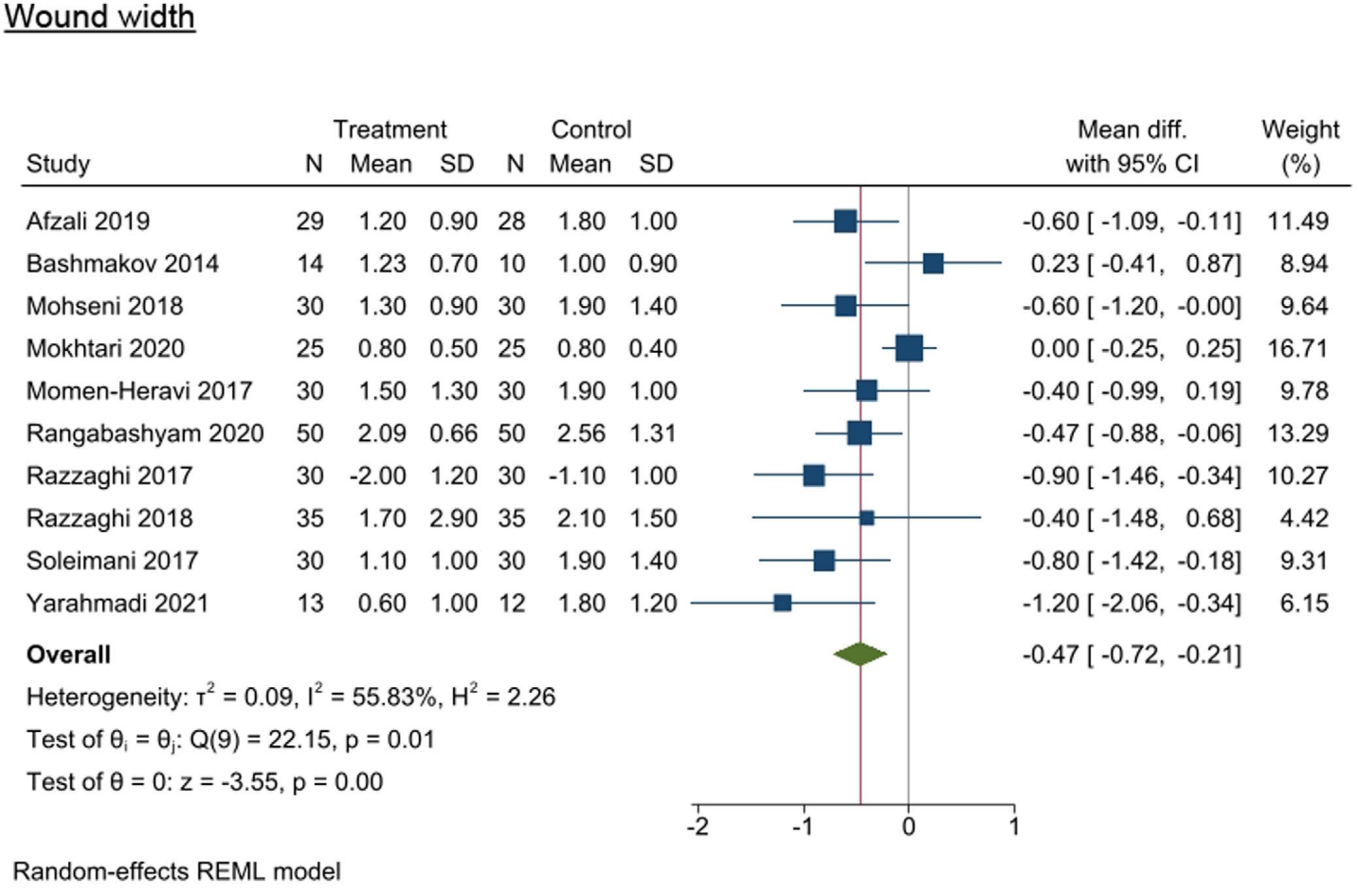
Nutrition intervention effect on wound width (cm) relative to all control interventions.

Results from all 10 studies exploring wound width (9 RCTS and 1 quasi‐experimental study) were eligible for inclusion in the meta‐analysis, which found a significant difference in wound width in the intervention group (WMD −0.466 [95% CI −0.724, −0.208], *I* ^2^ = 55.83%, *p* = 0.0004) compared with control (Figure 6).^21,52,54,60–62,64,65,67,71^

#### 3.14.3 Wound length

**FIGURE 7:**
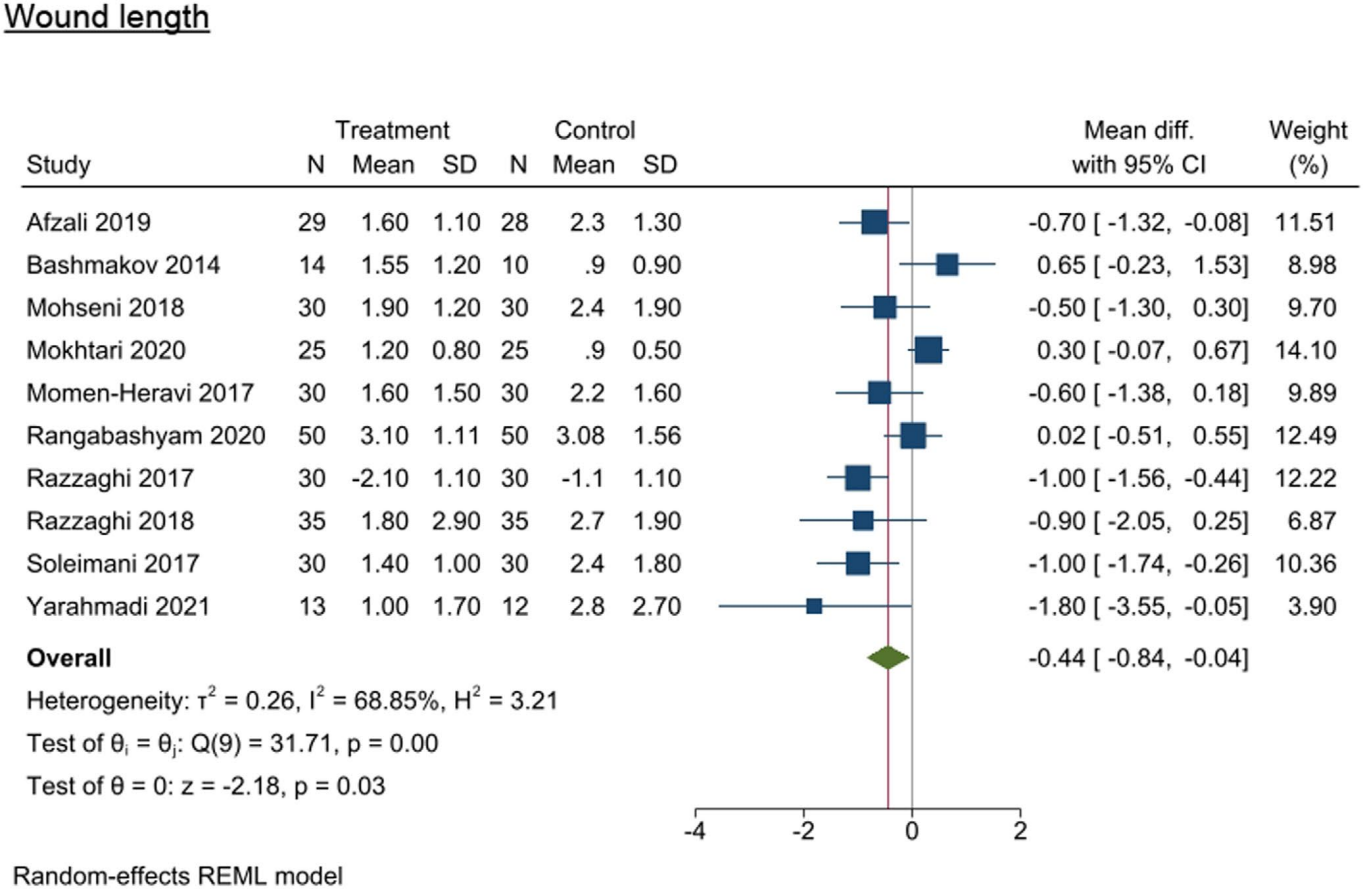
Nutrition intervention effect on wound length (cm) relative to all control interventions.

The meta‐analysis including 10 studies reporting wound length identified a significant difference between the intervention group (−0.443 [95% CI −0.841, −0.045], *I* ^2^ = 68.85%, *p* = 0.0292) compared with the control group.^21,52,54,60–62,64,65,67,71^ After imputing potential missing studies using the trim and fill method, the effect size reduced (WMD −0.08) and became non‐significant (95% CI −0.508, 0.354). However, for this to occur, it would require there to be at least 4 unpublished studies showing a negative effect of the intervention (Figure 7).

#### 3.14.4 Proportion of people healed

**FIGURE 8:**
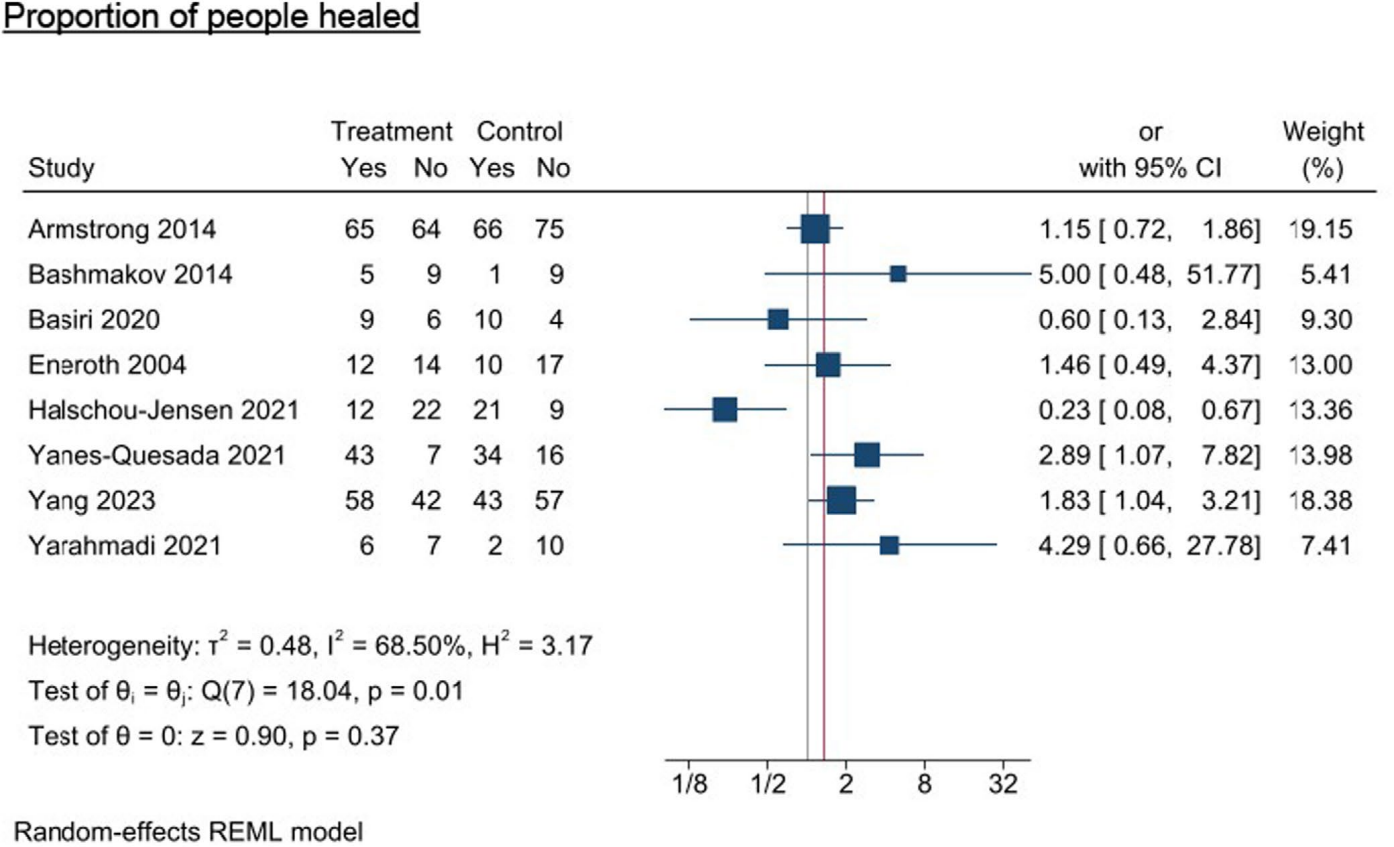
Nutrition intervention effect on the proportion of people (n) healed relative to all control interventions.

The proportion of people healed was measured in nine studies; however, only eight articles were eligible for inclusion.^53–56,58,66,67,69^ A retrospective case–control study exploring the proportion of people healed was excluded from the meta‐analysis as the nutrition intervention (multidisciplinary team including a dietitian) was very different to the supplement nutrition interventions included in the analysis.^72^ Due to the conflicting positive and negative results for wound healing, an overall effect was not calculated as it was judged not to be appropriate when also considering the high study heterogeneity (*I* ^2^ = 68.50%) and Rob‐2 risk of bias results. Two studies reported a significant positive effect,^66,69^ one had a significant negative effect,^58^ and five reported no statistically significant effects (Figure 8).^53–56,67^

### 3.15 GRADE assessment

For wound depth, width and length, and the proportion of people healed, the certainty of evidence was determined to be very low (Supplementary Material S3).

## 4 DISCUSSION

This systematic review and meta‐analysis aimed to summarise the quality of the contemporary scientific literature and evaluate the effectiveness of dietary interventions in the prevention and treatment of chronic wounds in individuals with diabetes. The meta‐analyses provide preliminary evidence that single and combination nutrient supplements may significantly reduce wound depth, length and width. However, the results should be interpreted with caution due to the quality of the evidence being low and the very low certainty of evidence. The supplements which demonstrated effectiveness in the included trials included zinc, probiotics, omega‐3, vitamin D, magnesium and/or vitamin E and vitamin C combined with vitamin E. However, studies utilising nanocurcumin and trans‐resveratrol did not report any significant improvements in wound healing.^54,61^ Although nutrient supplementation demonstrated improved wound healing, the limited data available precluded subgroup analysis. It is, therefore, not yet known if one specific supplement is superior to another or if any other interventions are effective.

The current study's findings are confirmatory of a recent prospective cohort study investigating factors contributing to DFU healing, which reported that nutrient supplementation was associated with 4.36 times increased odds of healing.^74^ Although the studies included in this systematic review primarily focused on single or two nutrients combined, the literature demonstrates a wide range of specific nutrients that contribute to metabolic processes in wound healing.^8,20,75^A recent review emphasised that isolated versions of several nutrients do not offer the same benefits as consuming these nutrients within whole foods.^76^ These discrepancies arise from the numerous interactions (positive and negative) that occur between nutrients, the food matrix and the other components in the food.^76^ For example, there is a positive relationship in whole apples between fibre and polyphenols; however, consuming clear apple juice that is low in fibre and high in fructose can have adverse nutritional effects, particularly on total cholesterol and LDL‐cholesterol.^77^ As the inaccessibility and bioavailability of nutrients are directly related to the food matrix, future research exploring nutrition interventions for wound healing in those with diabetes should also include whole foods.^76^ Food sources of the key nutrients effective in wound healing can be found in Supplementary Material S4 and Supplementary Table S9.

Although most included studies focused on supplementation, three of the included studies included nutrition education interventions. One RCT provided dietitian education,^55^ with one case–control study providing nurse‐led nutrition education.^69^ Both of these included studies reported improved wound healing compared with controls; however,^55,69^ one study, which used a multidisciplinary team including a dietitian, did not find significant differences between groups.^72^ Whilst dietitian interventions and nutrition education were uncommon, the results suggest that MNT improves wound healing, as well as other related factors that affect wound healing in people with diabetes, including glycaemic control. While there are limited intervention studies using dietary education for individuals with diabetes and chronic wounds specifically, research demonstrates that MNT can improve client knowledge and skills related to food and nutrition, and their relationship with blood glucose management, and should therefore be considered when implementing nutrition interventions for these population groups.

People with diabetes experience a range of different chronic wound aetiologies, with international guidelines recognising nutrition as an important consideration in people with DFU, PI and VLU.^8,9,78^ Despite this, the current review only uncovered studies completed in DFU populations meeting the eligibility criteria. Although some studies conducted in VLU and PI were identified, participants with diabetes in these studies were not able to be analysed separately as they were included in a larger cohort of people without diabetes. Given the specific nutritional considerations needed in people with diabetes, in relation to glycaemic control,^26^ this was an interesting finding. These results suggest that the influence of diabetes on dietary intake and interventions in wound healing is underestimated. For instance, an oral liquid supplement designed to support wound healing can have up to approximately 50 g of carbohydrate, which is more than three average slices of bread. A standard nutritional supplement with a large carbohydrate and low fibre content can compromise glycaemic control.^79^ Therefore, this warrants the need for additional nutrition intervention research, specifically in people with diabetes and other wound types, that considers these additional needs.

Dietary assessment underpins personalised dietary interventions; despite this, there was a distinct lack of any dietary assessment in the included studies. Dietary assessment alongside biochemistry needs to be considered in wound populations as it allows identification of nutrient deficiencies and determines the dietary intervention required.^80,81^ If an individual already has an adequate intake of nutrients needed to optimise wound healing from their diet, excessive intakes caused by additional supplementation can lead to adverse health effects if intake exceeds the upper limit.^82^ Furthermore, only one trial reported baseline and end of intervention dietary intakes utilising a 24‐hour recall,^55^ and only one study evaluated participants nutritional status,^69^ despite the high prevalence of malnutrition in this population.^13^, ^17^ Early identification of individuals who are malnourished or at risk of malnutrition and subsequent nutrition intervention by a dietitian is vital for timely and appropriate nutrition support to address malnutrition.^83^ Future trials utilising dietary interventions to support healing of chronic wounds in individuals with diabetes need to consider the inclusion of dietary assessment and malnutrition screening and assessment to ensure safe optimisation of nutrient status, as malnutrition can delay the wound healing process.^17^

Many of the included studies also evaluated secondary outcomes including blood inflammatory markers and lipid profiles. Antioxidant capacity is an important consideration for wound healing, as an imbalance between free radicals and antioxidants can lead to excess reactive oxygen species, resulting in subsequent cell and tissue damage and impaired wound healing.^84^ Findings from this systematic review also suggest that nutrition supplementation increases total antioxidant capacity (TAC), with TAC improving in five studies (probiotic,^60^ nano‐curcumin,^61^ zinc,^62^ omega‐3,^65^ and vitamin E + magnesium^52^). Studies exploring the role of these nutrients in TAC confirm these findings for probiotics, curcumin, zinc and omega‐3,^85–88^ with vitamin E being an antioxidant.^89^ Moreover, seven studies suggested nutrition supplementation reduced high‐sensitivity C‐reactive protein levels (Vitamin E + magnesium,^52^ probiotic,^60^ zinc,^62^ omega‐3,^65^ magnesium,^64^ and vitamin D^21,63^). According to the dietary inflammatory index, these nutrients (vitamin D, vitamin E, magnesium, zinc and omega‐3) are anti‐inflammatory.^90^ Probiotics are not included in the dietary inflammatory index. Conversely, the results from this systematic review conclude it is unlikely that nutrient supplements impact blood lipid levels. As diabetes is a risk factor for cardiovascular disease,^91^ consideration of MNT to support blood lipid management is important to minimise cardiovascular disease risk.^92^ From the three included studies providing nutrition education, none reported blood lipids; therefore, whether dietary education aids in improving blood lipid profile for individuals living with diabetes and a chronic wound remains unknown. However, a systematic review exploring the impact of dietetic consultation on blood lipids reported that dietetic counselling was effective in lowering triglycerides.^93^ Findings from two systematic reviews and meta‐analyses for total‐, HDL‐ and LDL‐cholesterol in people with pre‐diabetes had conflicting results, with one suggesting significant improvements^94^ and the other reporting inconsistent findings.^95^ Consequently, providing dietary advice rather than a single‐nutrient supplement may demonstrate larger improvements in lipid status. Therefore, tailoring nutrition interventions that benefit not just wound healing, but also other co‐morbidities is optimal.

The overall quality of evidence was graded to be very low, and the majority of studies were high/serious risk of bias with inconsistencies between studies and imprecision related to studies not reporting confidence intervals and/or statistical significance for between‐group differences. For example, some RCTs did not report randomisation methods or concealment, or did not have a pre‐specified analysis plan with some deviations from the protocol, which affected their bias evaluations. Some non‐randomised studies suggested baseline group differences. Therefore, the overall low quality and very low certainty of the studies suggest the need for more high‐quality RCTs with consistent methodologies and proper randomisation, and more consistent outcomes and reporting of outcomes in wound healing to allow for comparison to be made. The results are suggestive of a potential positive effect of nutrient supplements on wound healing. However, as studies had moderate to high risk of bias, there is potential for the findings of the current meta‐analysis to also be biased, which further highlights the need for more high‐quality RCTs in this area.

## 5 LIMITATIONS

Whilst a systematic search strategy was piloted and used, it is possible that some studies were missed. If data could not be extracted from the full text, authors were contacted for missing data. However, despite contacting numerous authors, we did not receive any responses. There was too small a number of studies per arm and insufficient data on potential predictors to run regression models to explain any additional heterogeneity in the outcome, or to explore the effect size within each nutrient supplementation type or other non‐supplementation dietary interventions.

Although two reviewers independently completed quality appraisal with a third reviewer resolving any disagreements, these tools can be subjective, and researcher bias may have occurred. Furthermore, one study that was retrieved from an included study reference list was not found in the search strategy. Therefore, there is potential for some studies being missed; however, this is unlikely given the rigorous search of intervention databases as well as the systematic search strategy applied. Moreover, the search was limited to those published in English, and therefore, studies published in other languages may have been missed. The data collection process was completed by only one researcher, with a second reviewer checking 10% of studies for data extraction, which increases the potential for human error.

## 6 CONCLUSION

There is low‐quality evidence that nutrition supplementation promotes wound healing in people with diabetes, in terms of achieving reductions in wound depth, width and length. However, the quality and certainty of the evidence were low or very low, and therefore, caution must be taken when interpreting the results. There was no difference between single and multi‐nutrient supplements and wound healing, and it is not clear if any specific supplement is superior to another. Nutrition interventions, including education, may be beneficial for wound healing; however, there is currently limited evidence available. The results of this systematic review also suggest that blood lipids are not impacted by nutrient supplements; however, the results suggest an anti‐inflammatory role of nutrient supplements, with an increase in total antioxidant capacity and a reduction in high‐sensitivity C‐reactive protein being identified, which supports wound healing. However, these results could not be meta‐analysed.

Due to the high risk of bias in studies included in the meta‐analysis, additional high‐quality RCT evaluating the effectiveness of dietary interventions are required to inform future high‐quality, robust meta‐analyses in order to identify the most effective dietary interventions for the prevention and management of chronic wounds in those living with diabetes. Moreover, the use of standardised methodologies and outcomes, with between‐group differences reported, would support understanding the effectiveness of dietary interventions for wound healing.

## AUTHOR CONTRIBUTIONS

HRD, PET and CEC designed the systematic review protocol. The search strategy was developed within the research team and with assistance from a senior research librarian with experience in health and medical research. HRD, NG and PIM completed title and abstract screening; HRD and NG completed full text screening; and HRD completed data extraction. PET resolved conflicts in the title and abstract screening and full text screening. HRD and PIM completed quality appraisal tools, and EDC resolved conflicts that arose in quality appraisal. HRD and LL completed the meta‐analysis, and PET and HRD completed GRADE assessments. HRD, PET, CEC and EDC drafted the manuscript. All authors reviewed the manuscript prior to submission.

## FUNDING INFORMATION

HRD is undertaking this systematic review as part of her PhD (Nutrition and Dietetics) at the University of Newcastle and is supported by a University of Newcastle research training program scholarship. CEC is supported by an NHMRC Leadership Research Fellowship (L3, APP2009340).

## CONFLICT OF INTEREST STATEMENT

The authors declare no conflicts of interest.

## Supporting information


Data S1.

## Data Availability

The data that support the findings of this study are available from the corresponding author upon reasonable request.
