# Supplementary material for: Effectiveness of dietary interventions in individuals with diabetes for preventing and healing chronic wounds; a systematic review with meta‐analysis
Source: Diabet Med. 2025 Jul 9;42(9):e70100. doi: 10.1111/dme.70100 (PMC12352720; doi:10.1111/dme.70100)
Supplement: Supplementary file 1 — Data S1. [file DME-42-e70100-s001.zip › dme70100-sup-0001-Supinfo01..pdf]

Supplementary Material 1. Search Strategies

EBSCOhost

#	Query	Limiters/Expanders	Last Run Via	Results
S51	S25 AND S46 AND S49	Limiters - English Language<br>Expanders - Apply equivalent subjects<br>Search modes - Boolean/Phrase	Interface - EBSCOhost Research Databases<br>Search Screen - Advanced Search<br>Database - CINAHL Complete	
S50	S25 AND S46 AND S49	Expanders - Apply equivalent subjects<br>Search modes - Boolean/Phrase	Interface - EBSCOhost Research Databases<br>Search Screen - Advanced Search<br>Database - CINAHL Complete	Display
S49	S47 OR S48	Expanders - Apply equivalent subjects<br>Search modes - Boolean/Phrase	Interface - EBSCOhost Research Databases<br>Search Screen - Advanced Search<br>Database - CINAHL Complete	Display
S48	"diabet*"	Expanders - Apply equivalent subjects<br>Search modes - Boolean/Phrase	Interface - EBSCOhost Research Databases<br>Search Screen - Advanced Search<br>Database - CINAHL Complete	Display
S47	(MH "Diabetes Mellitus, Type 2") OR (MH "Diabetes Mellitus") OR "diabetes" or "diabetes mellitus"	Expanders - Apply equivalent subjects<br>Search modes - Boolean/Phrase	Interface - EBSCOhost Research Databases<br>Search Screen - Advanced Search<br>Database - CINAHL Complete	Display
S46	S26 OR S27 OR S28 OR S29 OR S30 OR S31 OR S32 OR S33 OR S34 OR S35 OR S36 OR S37 OR S38 OR S39 OR S40 OR S41 OR S42 OR S43 OR S44 OR S45	Expanders - Apply equivalent subjects<br>Search modes - Boolean/Phrase	Interface - EBSCOhost Research Databases<br>Search Screen - Advanced Search<br>Database - CINAHL Complete	Display
S45	"decubitus*"	Expanders - Apply equivalent subjects<br>Search modes - Boolean/Phrase	Interface - EBSCOhost Research Databases<br>Search Screen - Advanced Search<br>Database - CINAHL Complete	Display

S44	TI diabet* N3 defect* OR AB diabet* N3 defect*	Expanders - Apply equivalent subjects<br>Search modes - Boolean/Phrase	Interface - EBSCOhost Research Databases<br>Search Screen - Advanced Search<br>Database - CINAHL Complete	Display
S43	TI diabet* N3 wound* OR AB diabet* N3 wound*	Expanders - Apply equivalent subjects<br>Search modes - Boolean/Phrase	Interface - EBSCOhost Research Databases<br>Search Screen - Advanced Search<br>Database - CINAHL Complete	Display
S42	TI (diabet* N3 (foot or feet)) OR AB (diabet* N3 (foot or feet))	Expanders - Apply equivalent subjects<br>Search modes - Boolean/Phrase	Interface - EBSCOhost Research Databases<br>Search Screen - Advanced Search<br>Database - CINAHL Complete	Display
S41	TI diabet* N3 ulcer* OR AB diabet* N3 ulcer*	Expanders - Apply equivalent subjects<br>Search modes - Boolean/Phrase	Interface - EBSCOhost Research Databases<br>Search Screen - Advanced Search<br>Database - CINAHL Complete	Display
S40	(MH "Diabetic Foot") OR "diabetic foot ulcer*"	Expanders - Apply equivalent subjects<br>Search modes - Boolean/Phrase	Interface - EBSCOhost Research Databases<br>Search Screen - Advanced Search<br>Database - CINAHL Complete	Display
S39	"chronic foot ulcer*"	Expanders - Apply equivalent subjects<br>Search modes - Boolean/Phrase	Interface - EBSCOhost Research Databases<br>Search Screen - Advanced Search<br>Database - CINAHL Complete	Display
S38	"isch#emic ulcer*"	Expanders - Apply equivalent subjects<br>Search modes - Boolean/Phrase	Interface - EBSCOhost Research Databases<br>Search Screen - Advanced Search<br>Database - CINAHL Complete	Display
S37	"arterial ulcer*"	Expanders - Apply equivalent subjects<br>Search modes - Boolean/Phrase	Interface - EBSCOhost Research Databases<br>Search Screen - Advanced Search<br>Database - CINAHL Complete	Display
S36	"ulcus cruris"	Expanders - Apply equivalent subjects<br>Search modes - Boolean/Phrase	Interface - EBSCOhost Research Databases<br>Search Screen - Advanced	Display

			Search<br>Database - CINAHL Complete	
S35	"crural ulcer*"	Expanders - Apply equivalent subjects<br>Search modes - Boolean/Phrase	Interface - EBSCOhost Research Databases<br>Search Screen - Advanced Search<br>Database - CINAHL Complete	Display
S34	"stasis ulcer*"	Expanders - Apply equivalent subjects<br>Search modes - Boolean/Phrase	Interface - EBSCOhost Research Databases<br>Search Screen - Advanced Search<br>Database - CINAHL Complete	Display
S33	"varicose ulcer*"	Expanders - Apply equivalent subjects<br>Search modes - Boolean/Phrase	Interface - EBSCOhost Research Databases<br>Search Screen - Advanced Search<br>Database - CINAHL Complete	Display
S32	(MH "Venous Ulcer") OR "venous leg ulcer*"	Expanders - Apply equivalent subjects<br>Search modes - Boolean/Phrase	Interface - EBSCOhost Research Databases<br>Search Screen - Advanced Search<br>Database - CINAHL Complete	Display
S31	(MH "Leg Ulcer") OR "leg ulcer*"	Expanders - Apply equivalent subjects<br>Search modes - Boolean/Phrase	Interface - EBSCOhost Research Databases<br>Search Screen - Advanced Search<br>Database - CINAHL Complete	Display
S30	(MH "Foot Ulcer") OR "foot ulcer*"	Expanders - Apply equivalent subjects<br>Search modes - Boolean/Phrase	Interface - EBSCOhost Research Databases<br>Search Screen - Advanced Search<br>Database - CINAHL Complete	Display
S29	"pressure injury"	Expanders - Apply equivalent subjects<br>Search modes - Boolean/Phrase	Interface - EBSCOhost Research Databases<br>Search Screen - Advanced Search<br>Database - CINAHL Complete	Display
S28	(MH "Pressure Ulcer") OR (MH "Heel Ulcer")	Expanders - Apply equivalent subjects<br>Search modes - Boolean/Phrase	Interface - EBSCOhost Research Databases<br>Search Screen - Advanced Search<br>Database - CINAHL Complete	Display

S27	(MH "Wound Healing") OR "wound healing"	Expanders - Apply equivalent subjects<br>Search modes - Boolean/Phrase	Interface - EBSCOhost Research Databases<br>Search Screen - Advanced Search<br>Database - CINAHL Complete	Display
S26	"wound*"	Expanders - Apply equivalent subjects<br>Search modes - Boolean/Phrase	Interface - EBSCOhost Research Databases<br>Search Screen - Advanced Search<br>Database - CINAHL Complete	Display
S25	S1 OR S2 OR S3 OR S4 OR S5 OR S6 OR S7 OR S8 OR S9 OR S10 OR S11 OR S12 OR S13 OR S14 OR S15 OR S16 OR S17 OR S18 OR S19 OR S20 OR S21 OR S22 OR S23 OR S24	Expanders - Apply equivalent subjects<br>Search modes - Boolean/Phrase	Interface - EBSCOhost Research Databases<br>Search Screen - Advanced Search<br>Database - CINAHL Complete	Display
S24	"feeding"	Expanders - Apply equivalent subjects<br>Search modes - Boolean/Phrase	Interface - EBSCOhost Research Databases<br>Search Screen - Advanced Search<br>Database - CINAHL Complete	Display
S23	(MH "Nutritional Support") OR "nutritional support" OR (MH "Parenteral Nutrition") OR (MH "Enteral Nutrition")	Expanders - Apply equivalent subjects<br>Search modes - Boolean/Phrase	Interface - EBSCOhost Research Databases<br>Search Screen - Advanced Search<br>Database - CINAHL Complete	Display
S22	TI ((arginine or glutamine or “b-hydroxy-b-methylbutyrate” or “omega-3” or “omega-3 fatty acid*” or probiotic* or antioxidant) N3 (supplement* or fortification or capsule* or tablet* or liquid*)) OR AB ((arginine or glutamine or “b-hydroxy-b-methylbutyrate” or “omega-3” or “omega-3 fatty acid*” or probiotic* or antioxidant) N3 (supplement* or	Expanders - Apply equivalent subjects<br>Search modes - Boolean/Phrase	Interface - EBSCOhost Research Databases<br>Search Screen - Advanced Search<br>Database - CINAHL Complete	Display

	fortification or capsule* or tablet* or liquid*))			
S21	TI (nutrient* N3 (supplement* or fortification or capsule* or tablet* or liquid*)) OR AB (nutrient* N3 (supplement* or fortification or capsule* or tablet* or liquid*))	Expanders - Apply equivalent subjects<br>Search modes - Boolean/Phrase	Interface - EBSCOhost Research Databases<br>Search Screen - Advanced Search<br>Database - CINAHL Complete	Display
S20	TI ((macronutrient* or macro-nutrient* or protein* or "amino acid*" or carbohydrate* or calorie* or energ* or fat* or lipid*) N3 (supplement* or fortification or capsule* or tablet* or liquid*)) OR AB ((macronutrient* or macro-nutrient* or protein* or "amino acid*" or carbohydrate* or calorie* or energ* or fat* or lipid*) N3 (supplement* or fortification or capsule* or tablet* or liquid*))	Expanders - Apply equivalent subjects<br>Search modes - Boolean/Phrase	Interface - EBSCOhost Research Databases<br>Search Screen - Advanced Search<br>Database - CINAHL Complete	Display
S19	TI ((micronutrient* or "micro-nutrient*" or vitamin* or multivitamin* or mineral* or "trace next element*" or zinc or iodine or iron or cobalt or chromium or copper or manganese or magnesium or fluoride or sodium or selenium or molybdenum or “vitamin A” or “vitamin B9” or “vitamin C” or “ascorbic acid” or “vitamin D” or “vitamin E” or ��folic acid”) N3 (supplement* or fortification or capsule* or tablet* or liquid*)) OR AB ((micronutrient* or "micro-nutrient*" or vitamin* or multivitamin* or mineral* or "trace next element*" or	Expanders - Apply equivalent subjects<br>Search modes - Boolean/Phrase	Interface - EBSCOhost Research Databases<br>Search Screen - Advanced Search<br>Database - CINAHL Complete	Display

	zinc or iodine or iron or cobalt or chromium or copper or manganese or magnesium or fluoride or sodium or selenium or molybdenum or “vitamin A” or “vitamin B9” or “vitamin C” or “ascorbic acid” or “vitamin D” or “vitamin E” or “folic acid”) N3 (supplement* or fortification or capsule* or tablet* or liquid*))			
S18	(MH "Micronutrients") OR "micronutrients"	Expanders - Apply equivalent subjects<br>Search modes - Boolean/Phrase	Interface - EBSCOhost Research Databases<br>Search Screen - Advanced Search<br>Database - CINAHL Complete	Display
S17	(MH "Energy Intake") OR "energy intake"	Expanders - Apply equivalent subjects<br>Search modes - Boolean/Phrase	Interface - EBSCOhost Research Databases<br>Search Screen - Advanced Search<br>Database - CINAHL Complete	Display
S16	"dietary carbohydrates" OR "dietary proteins" OR "dietary fats"	Expanders - Apply equivalent subjects<br>Search modes - Boolean/Phrase	Interface - EBSCOhost Research Databases<br>Search Screen - Advanced Search<br>Database - CINAHL Complete	Display
S15	(MH "Dietary Carbohydrates") OR (MH "Dietary Proteins") OR (MH "Dietary Fats")	Expanders - Apply equivalent subjects<br>Search modes - Boolean/Phrase	Interface - EBSCOhost Research Databases<br>Search Screen - Advanced Search<br>Database - CINAHL Complete	Display
S14	(MH "Nutrients") OR "nutrients*"	Expanders - Apply equivalent subjects<br>Search modes - Boolean/Phrase	Interface - EBSCOhost Research Databases<br>Search Screen - Advanced Search<br>Database - CINAHL Complete	Display
S13	"oral supplement*"	Expanders - Apply equivalent subjects<br>Search modes - Boolean/Phrase	Interface - EBSCOhost Research Databases<br>Search Screen - Advanced Search<br>Database - CINAHL Complete	Display
S12	"supplement*"	Expanders - Apply equivalent subjects	Interface - EBSCOhost Research Databases	Display

		Search modes - Boolean/Phrase	Search Screen - Advanced Search<br>Database - CINAHL Complete	
S11	(MH "Dietary Supplements") OR (MH "Dietary Supplementation")	Expanders - Apply equivalent subjects<br>Search modes - Boolean/Phrase	Interface - EBSCOhost Research Databases<br>Search Screen - Advanced Search<br>Database - CINAHL Complete	Display
S10	"nutri* supplement*"	Expanders - Apply equivalent subjects<br>Search modes - Boolean/Phrase	Interface - EBSCOhost Research Databases<br>Search Screen - Advanced Search<br>Database - CINAHL Complete	Display
S9	"nutri* intervention"	Expanders - Apply equivalent subjects<br>Search modes - Boolean/Phrase	Interface - EBSCOhost Research Databases<br>Search Screen - Advanced Search<br>Database - CINAHL Complete	Display
S8	"diet* intervention"	Expanders - Apply equivalent subjects<br>Search modes - Boolean/Phrase	Interface - EBSCOhost Research Databases<br>Search Screen - Advanced Search<br>Database - CINAHL Complete	Display
S7	(MH "Nutritional Counseling") OR "nutri* counsel*"	Expanders - Apply equivalent subjects<br>Search modes - Boolean/Phrase	Interface - EBSCOhost Research Databases<br>Search Screen - Advanced Search<br>Database - CINAHL Complete	Display
S6	(MH "Nutrition Education") OR "nutri* education"	Expanders - Apply equivalent subjects<br>Search modes - Boolean/Phrase	Interface - EBSCOhost Research Databases<br>Search Screen - Advanced Search<br>Database - CINAHL Complete	Display
S5	(MH "Dietitians") OR "dieti?ian" OR (MH "Nutrition Services") OR (MH "Dietetics")	Expanders - Apply equivalent subjects<br>Search modes - Boolean/Phrase	Interface - EBSCOhost Research Databases<br>Search Screen - Advanced Search<br>Database - CINAHL Complete	Display
S4	"nutri* therapy"	Expanders - Apply equivalent subjects<br>Search modes - Boolean/Phrase	Interface - EBSCOhost Research Databases<br>Search Screen - Advanced Search<br>Database - CINAHL Complete	Display

S3	(MH "Nutrition") OR "nutrition*" OR (MH "Diet Therapy")	Expanders - Apply equivalent subjects<br>Search modes - Boolean/Phrase	Interface - EBSCOhost Research Databases<br>Search Screen - Advanced Search<br>Database - CINAHL Complete	Display
S2	(MH "Food") OR "food"	Expanders - Apply equivalent subjects<br>Search modes - Boolean/Phrase	Interface - EBSCOhost Research Databases<br>Search Screen - Advanced Search<br>Database - CINAHL Complete	Display
S1	(MH "Diet") OR "diet*"	Expanders - Apply equivalent subjects<br>Search modes - Boolean/Phrase	Interface - EBSCOhost Research Databases<br>Search Screen - Advanced Search<br>Database - CINAHL Complete	Display

Search Name: SR Wounds with Diabetes new search for 2023

Date Run: 13/03/2024 05:00:44

Comment: COCHRANE

ID	Search	Hits
#1	MeSH descriptor: [Diet] explode all trees	26550
#2	diet*:ti,ab,kw	116352
#3	nutrition*:ti,ab,kw	55378
#4	MeSH descriptor: [Nutrition Therapy] this term only	304
#5	"nutri* therapy":ti,ab,kw	0
#6	MeSH descriptor: [Nutritionists] this term only	82
#7	dieti?ian:ti,ab,kw	3276
#8	"nutri* education":ti,ab,kw	0
#9	"nutrition* counsel*":ti,ab,kw	0
#10	"diet* intervention":ti,ab,kw	1211
#11	"nutri* intervention":ti,ab,kw	1
#12	MeSH descriptor: [Dietary Supplements] explode all trees	19873
#13	"nutri* supplement*":ti,ab,kw	0
#14	"diet* supplement*":ti,ab,kw	46
#15	supplement*:ti,ab,kw	87519
#16	"oral supplement*":ti,ab,kw	302
#17	MeSH descriptor: [Nutrients] explode all trees	6731
#18	nutrients:ti,ab,kw	3897
#19	MeSH descriptor: [Micronutrients] 2 tree(s) exploded	6433
#20	Micronutrients:ti,ab,kw	2604
#21	MeSH descriptor: [Energy Intake] this term only	6095
#22	"energy intake":ti,ab,kw	9896
#23	MeSH descriptor: [Dietary Fats] 1 tree(s) exploded	9991
#24	"dietary fats":ti,ab,kw	5089
#25	MeSH descriptor: [Dietary Proteins] 1 tree(s) exploded	5042
#26	"dietary proteins":ti,ab,kw	3347

#27 MeSH descriptor: [Dietary Carbohydrates] 1 tree(s) exploded 8161

#28 "dietary carbohydrates":ti,ab,kw 3990

#29 MeSH descriptor: [Nutritional Support] explode all trees 4408

#30 "nutritional support":ti,ab,kw 2477

#31 MeSH descriptor: [Parenteral Nutrition] explode all trees 2023

#32 "parenteral nutrition":ti,ab,kw 4615

#33 MeSH descriptor: [Enteral Nutrition] explode all trees 2478

#34 "enteral nutrition":ti,ab,kw 5334

#35 #1 or #2 or #3 or #4 or #5 or #6 or #7 or #8 or #9 or #10 or #11 or #12 or #13 or #14 or #15 or #16 or #17 or #18 or #19 or #21 or #22 or #23 or #24 or #25 or #26 or #27 or #28 or #29 or #30 or #31 or #32 or #33 or #34 201591

#36 MeSH descriptor: [Wounds and Injuries] this term only 4279

#37 wound*:ti,ab,kw 39716

#38 MeSH descriptor: [Wound Healing] this term only 6293

#39 "wound healing":ti,ab,kw 13980

#40 MeSH descriptor: [Pressure Ulcer] explode all trees 1049

#41 "pressure ulcer*":ti,ab,kw 1578

#42 "pressure sore":ti,ab,kw194

#43 "pressure injury":ti,ab,kw 259

#44 MeSH descriptor: [Foot Ulcer] 1 tree(s) exploded 1599

#45 "foot ulcer*":ti,ab,kw 1641

#46 MeSH descriptor: [Diabetic Foot] this term only 1496

#47 "diabetic foot ulcer*":ti,ab,kw 924

#48 "chronic foot ulcer*":ti,ab,kw 8

#49 MeSH descriptor: [Varicose Ulcer] explode all trees 841

#50 "venous ulcer*":ti,ab,kw 329

#51 "venous leg ulcer*":ti,ab,kw 273

#52 MeSH descriptor: [Leg Ulcer] this term only 623

#53 "leg ulcer*":ti,ab,kw 1338

#54 "arterial ulcer*":ti,ab,kw 10

#55 "decubitus*":ti,ab,kw 1865

#56 #36 or #37 or #38 or #39 or #40 or #41 or #42 or #43 or #44 or #45 or #46 or #47 or #48 or #49 or #50 or #51 or #52 or #53 or #54 or #55 43549

#57 #35 and #56 2516

#58 MeSH descriptor: [Diabetes Mellitus] 1 tree(s) exploded 40382

#59 diabetes:ti,ab,kw 106231

#60 diabetic:ti,ab,kw 42782

#61 #58 or #59 or #60 120234

#62 #35 and #56 and #61

Embase Search Strategy

1.	diet therapy/ or diet/ or diet*.mp.
2.	nutrition* counsel*.mp.
3.	dietitian/ or dieti?ian.mp.
4.	nutri* therapy.mp. or enteric feeding/ or parenteral nutrition/
5.	feeding.mp.
6.	food.mp.
7.	nutrition education/ or nutri* education.mp.
8.	energy intake.mp. or caloric intake/
9.	dietary supplement/ or diet supplementation/ or diet* supplement*.mp.
10.	dietary carbohydrates.mp. or carbohydrate intake/
11.	dietary fats.mp. or fat intake/
12.	dietary proteins.mp. or protein intake/
13.	supplement*.mp.
14.	nutrition supplement/ or nutri* supplement*.mp.
15.	nutrition*.mp. or nutrition/
16.	diet* intervention.mp.
17.	nutri* intervention.mp.
18.	nutrients.mp. or nutrient/
19.	micronutrients.mp. or trace element/
20.	((micronutrient* or "micro-nutrient*" or vitamin* or multivitamin* or mineral* or "trace next element*" or zinc or iodine or iron or cobalt or chromium or copper or manganese or magnesium or fluoride or sodium or selenium or molybdenum or "vitamin A" or "vitamin B9" or "vitamin C" or "ascorbic acid" or "vitamin D" or "vitamin E" or "folic acid") adj3 (supplement* or fortification or capsule* or tablet* or liquid*)).mp.
21.	(nutrient* adj3 (supplement* or fortification or capsule* or tablet* or liquid*)).mp.
22.	((macronutrient* or "macro-nutrient*" or protein* or "amino next acid*" or carbohydrate* or calorie* or energ* or fat* or lipid*) adj3 (supplement* or fortification or capsule* or tablet* or liquid)).mp.
23.	((arginine or glutamine or "b-hydroxy-b-methylbutyrate" or "omega-3" or "omega-3 fatty acid*" or probiotic* or antioxidant) adj3 (supplement* or fortification or capsule* or tablet* or liquid*)).mp.
24.	oral supplement*.mp.
25.	nutritional support.mp. or nutritional support/
26.	1 or 2 or 3 or 4 or 5 or 6 or 7 or 8 or 9 or 10 or 11 or 12 or 13 or 14 or 15 or 16 or 17 or 18 or 19 or 20 or 21 or 22 or 23 or 24 or 25
27.	wound*.mp. or wound/ or wound healing/
28.	pressure ulcer*.mp.
29.	pressure injury.mp.
30.	pressure sore.mp.
31.	decubitus/ or decubitus*.mp.
32.	foot ulcer/ or foot ulcer*.mp.
33.	diabetic foot/ or diabetic foot ulcer*.mp.
34.	(diabet* adj3 ulcer*).mp.
35.	(diabet* adj3 (foot or feet)).mp.
36.	(diabet* adj3 wound*).mp.

Embase Search Strategy

37.	(diabet* adj3 defect*).mp.
38.	leg ulcer/
39.	((arterial or ischaemic or ischemic) adj (wound* or ulcer*)).mp.
40.	venous ulcer*.mp.
41.	(venous leg ulcer* or varicose ulcer* or stasis ulcer* or crural ulcer* or ulcus cruris).mp.
42.	chronic foot ulcer*.mp.
43.	27 or 28 or 29 or 30 or 31 or 32 or 33 or 34 or 35 or 36 or 37 or 38 or 39 or 40 or 41 or 42
44.	diabetes.mp. or diabetes mellitus/
45.	diabet*.mp.
46.	diabetes mellitus.mp.
47.	44 or 45 or 46
48.	26 and 43 and 47
49.	limit 48 to english language
50.	animals/ not humans/
51.	49 not 50
