# Supplementary material for: Effectiveness of dietary interventions in individuals with diabetes for preventing and healing chronic wounds; a systematic review with meta‐analysis
Source: Diabet Med. 2025 Jul 9;42(9):e70100. doi: 10.1111/dme.70100 (PMC12352720; doi:10.1111/dme.70100)
Supplement: Supplementary file 1 — Data S1. [file DME-42-e70100-s001.zip › dme70100-sup-0002-Supinfo02.docx]

**Supplementary Material 2. Meta-analysis additional information**

Although there was evidence of unexplained heterogeneity for wound depth, width and length, and proportion of people healed, there was insufficient information on potential predictors to adequately model this further via meta-regression.

# Wound Depth

Sensitivity analyses excluding non-RCTs ( -0.217 [95%CI -0.405, -0.028], p = 0.0243) or per-protocol studies (MWD -0.186 [95%CI -0.369, -0.002], p = 0.0476) both showed no meaningful differences in the effect estimate, statistical significance or model fit when compared to the main results. The trim and fill method were utilised to assess potential bias from missing studies, which did not impact the results significantly.

**Wound depth meta-analysis summary random-effects model**

**
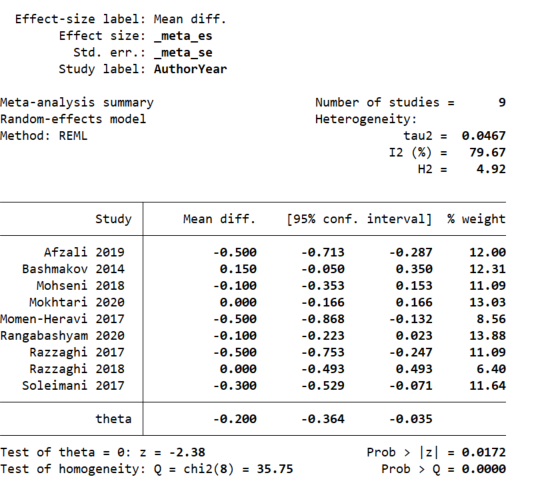
**

**Wound depth meta-regression on follow-up time**

**
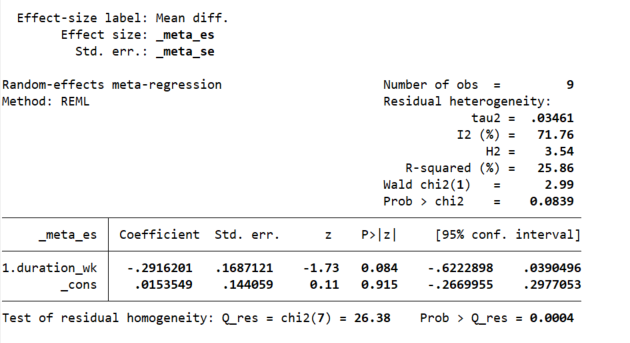
**

**Wound depth sensitivity analysis to exclude the one non-RCT study**

**
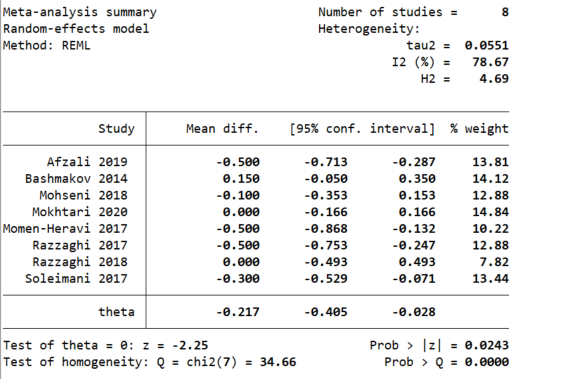
**

**Wound depth sensitivity analysis to exclude the two per-protocol papers**

**
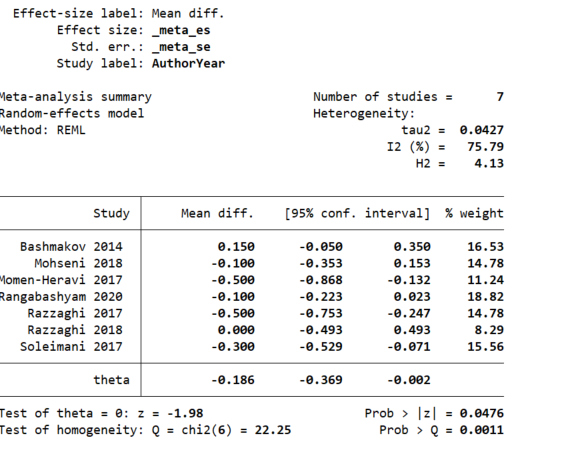
**

**Wound depth sensitivity analysis to exclude the adjusted results papers**

Not completed as all studies reported non-adjusted data.

**Wound depth funnel plot**


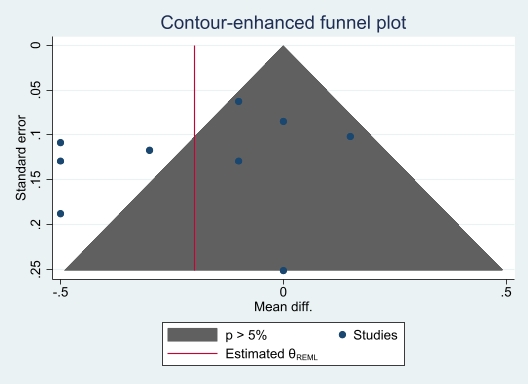


**Wound depth funnel plot with trim fill function**


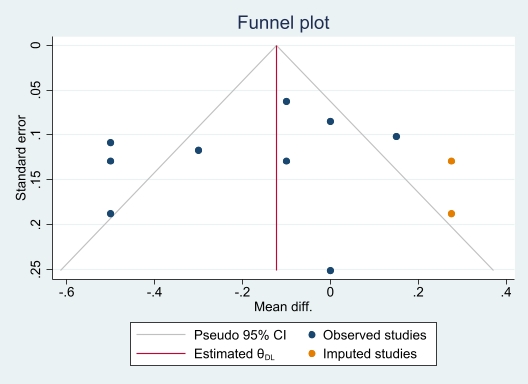


# Wound width

A sensitivity analysis excluding the non-RCT showed a slightly stronger effect size compared to the main results (-0.474 [95%CI -0.770, -0.178, p = 0.0017). Another sensitivity analysis excluding the two per-protocol papers suggested a similar effect size and remained significant, as well as significantly less heterogeneity in the model (tau-square and i-square tests decreased and the test for homogeneity was no longer rejected) (-0.550 [95%CI -0.815, -0.284, p = 0.0000). After accounting for potential missing studies, the trim and fill method did not impact the effect size or statistical significance.

**Wound width meta-analysis summary random-effects model**

**
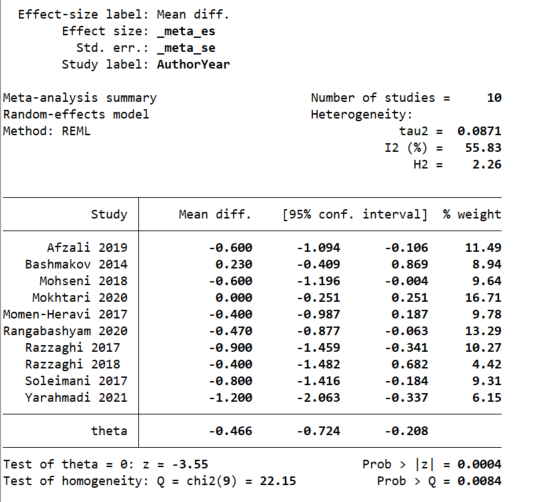
**

**Wound width meta-regression on follow-up time**

**
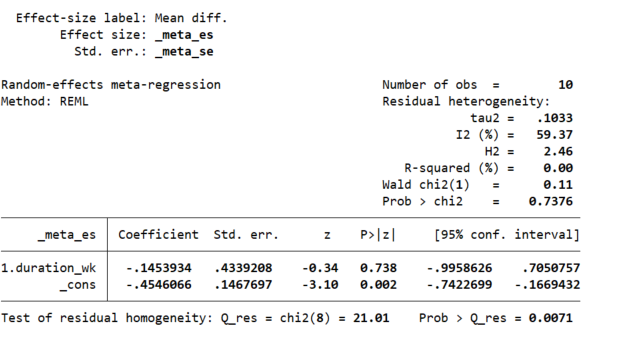
**

**Wound width sensitivity analysis to exclude the one non-RCT study**

**
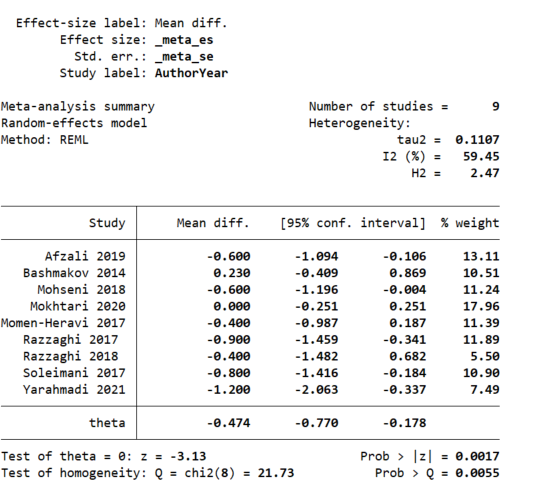
**

**Wound width sensitivity analysis to exclude the one per-protocol paper**

**
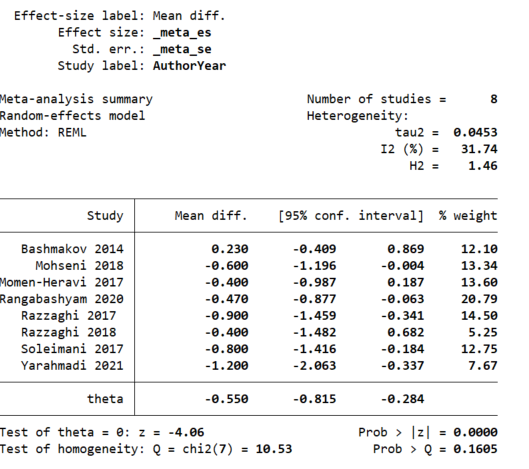
**

**Wound width sensitivity analysis to exclude the adjusted results papers**

Not completed as all studies reported non-adjusted data.

**Wound width funnel plot**

**
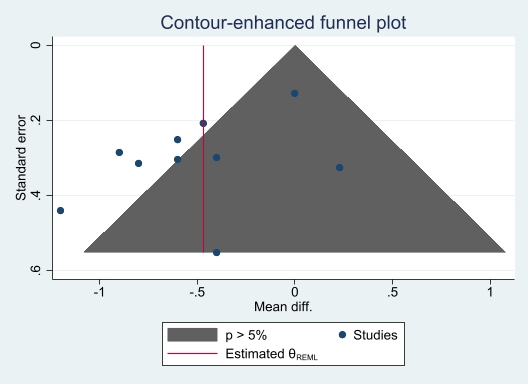
**

**Wound width funnel plot with trim fill function**

**
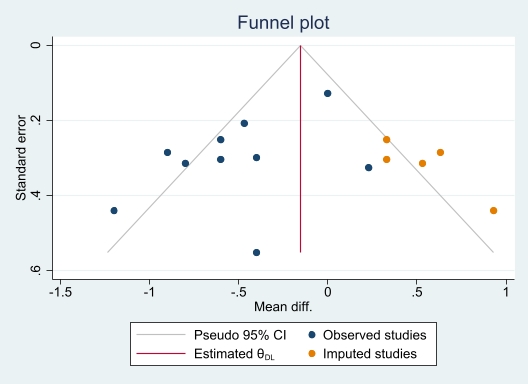
**

# Wound length

Following exclusion of non-randomised studies, the effect size increased slightly, however overall, there was no significant changes to the interpretation or conclusions drawn from the main results (-0.513 [95%CI -0.951, -0.074, p = 0.0219). An additional sensitivity analyses excluding the 2 per-protocol papers demonstrated no significant changes to the effect estimate, statistical significance or model fit when the per-protocol papers were excluded (-0.541 [95%CI -0.987, -0.096, p = 0.0172).

**Wound length meta-analysis summary random-effects model**

**
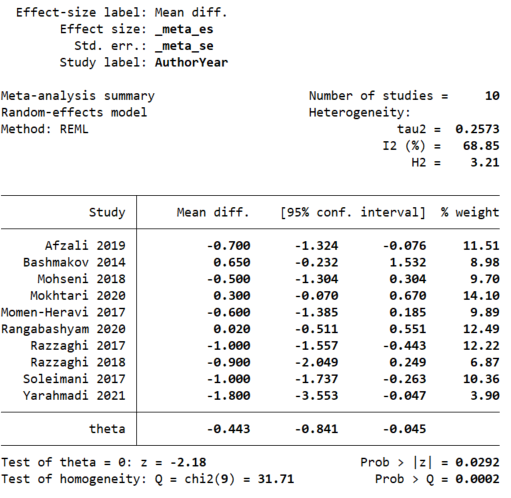
**

**Wound length meta-regression on follow-up time**

**
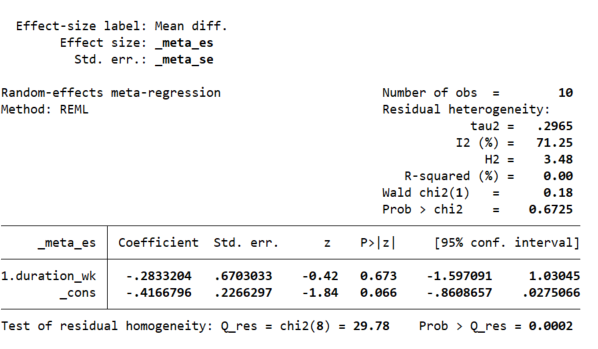
**

**Wound length sensitivity analysis to exclude the one non-RCT study**

**
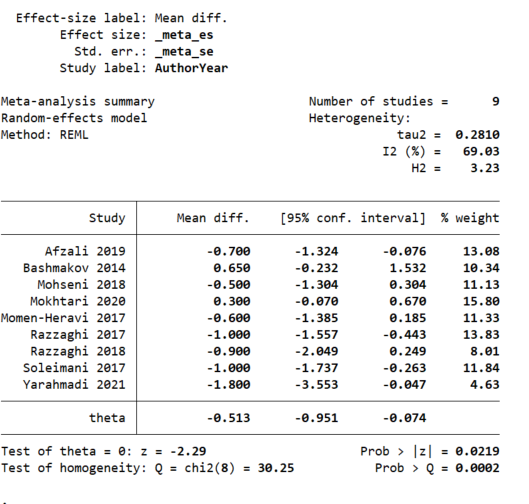
**

**Wound length sensitivity analysis to exclude the two per-protocol papers**

**
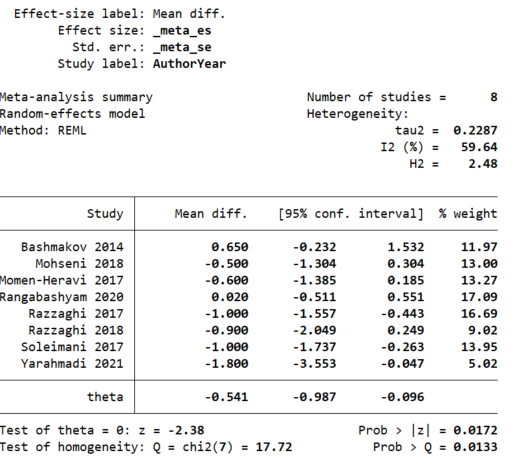
**

**Wound length sensitivity analysis to exclude the adjusted results papers**

Not completed as all studies reported non-adjusted data.

**Wound length funnel plot**

**
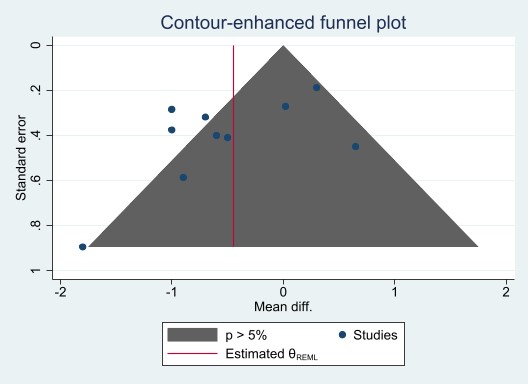
**

**Wound length funnel plot with trim fill function**

**
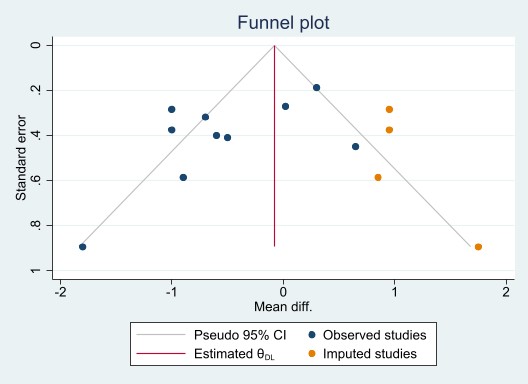
**

# Proportion of people healed

A sensitivity analysis excluding the one per-protocol paper (1.459 [95%CI 0.733, 2.905, p = 0.2823) and other sensitivity analysis excluding the one study only presenting adjusted data (1.177 [95%CI 0.600, 2.310, p = 0.6349), did not significantly change the results. Moreover, exclusion of non-randomised studies in another sensitivity analysis did not change significantly from the main results (1.264 [95%CI 0.588, 2.719, p = 0.5488). Following imputing potential missing studies, the trim and fill method did not significantly impact the effect size or change our conclusions.

**Proportion of people healed meta-analysis summary random-effects model**

**
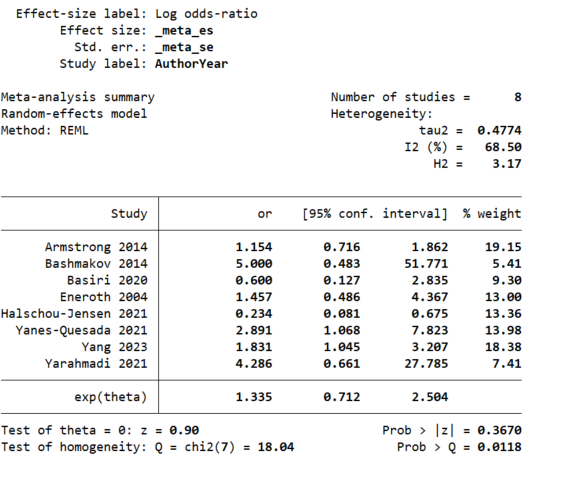
**

**Proportion of people healed sensitivity analysis to exclude the one non-RCT study**

**
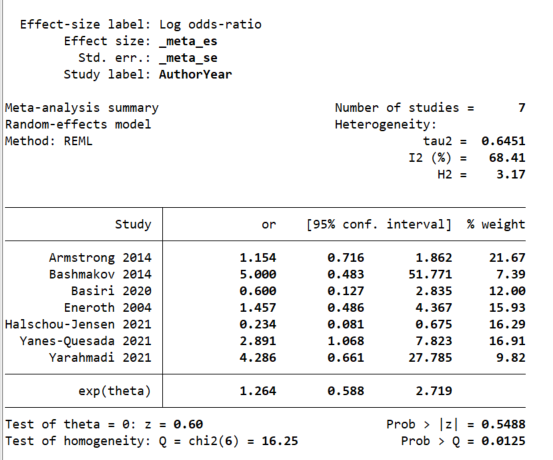
**

**Proportion of people healed sensitivity analysis to exclude the one per-protocol paper**

**
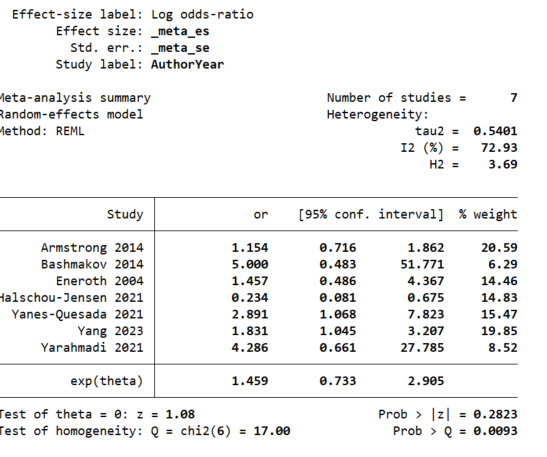
**

**Proportion of people healed sensitivity analysis to exclude the one adjusted results paper**

**
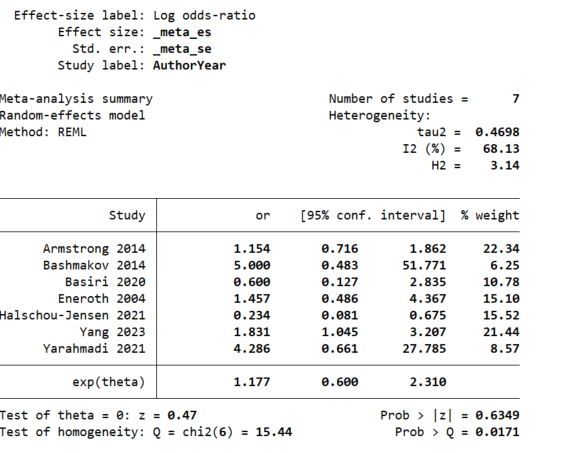
**

**Proportion of people healed funnel plot**

**
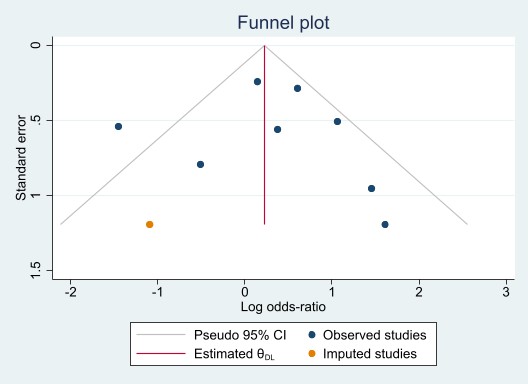
**

**Proportion of people healed funnel plot for trim fill function**

**
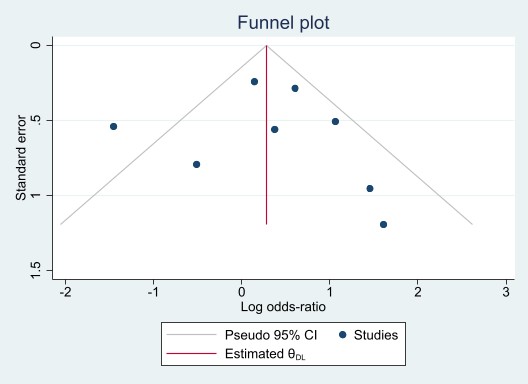
**
