# Supplementary material for: Effectiveness of dietary interventions in individuals with diabetes for preventing and healing chronic wounds; a systematic review with meta‐analysis
Source: Diabet Med. 2025 Jul 9;42(9):e70100. doi: 10.1111/dme.70100 (PMC12352720; doi:10.1111/dme.70100)
Supplement: Supplementary file 1 — Data S1. [file DME-42-e70100-s001.zip › dme70100-sup-0003-Supinfo03.docx]

**Question:** Dietary Intervention compared to control/placebo for wound healing in people with diabetes

| **Certainty assessment** | | | | | | | **№ of patients** | | **Effect** | | **Certainty** | **Importance** |
| --- | --- | --- | --- | --- | --- | --- | --- | --- | --- | --- | --- | --- |
| **№ of studies** | **Study design** | **Risk of bias** | **Inconsistency** | **Indirectness** | **Imprecision** | **Other considerations** | **Dietary Intervention** | **control/placebo** | **Relative (95% CI)** | **Absolute (95% CI)** |  |  |
| **Wound Depth (follow-up: range 6 weeks to 12 weeks)** | | | | | | | | | | | | |
| 9 | randomised trials | serious^a^ | serious^b,c^ | not serious | serious^d^ | none | 273 | 268 | - | MD **0.2 lower** (0.36 lower to 0.04 lower) | ⨁◯◯◯ Very low^a,b,c,d^ | IMPORTANT |
| **Wound Width (follow-up: range 6 weeks to 12 weeks)** | | | | | | | | | | | | |
| 10 | randomised trials | serious^e^ | serious^f^ | not serious | serious^d^ | none | 286 | 280 | - | MD **0.47 lower** (0.72 lower to 0.21 lower) | ⨁◯◯◯ Very low^d,e,f^ | IMPORTANT |
| **Wound Length (follow-up: range 6 weeks to 12 weeks)** | | | | | | | | | | | | |
| 10 | randomised trials | serious^e^ | serious^f^ | not serious | serious^b,d^ | none | 286 | 280 | - | MD **0.44 lower** (0.84 lower to 0.05 lower) | ⨁◯◯◯ Very low^b,d,e,f^ | IMPORTANT |
| **Proportion of People Healed (follow-up: range 8 weeks to 52 weeks)** | | | | | | | | | | | | |
| 8 | randomised trials | very serious^g^ | serious^b,d^ | not serious | serious^d^ | none | 210/371 (56.6%) | 187/378 (49.5%) | **OR 1.34** (0.71 to 2.50) | **73 more per 1,000** (from 85 fewer to 215 more) | ⨁◯◯◯ Very low^b,d,g^ | CRITICAL |

**CI:** confidence interval; **MD:** mean difference; **OR:** odds ratio

#### Explanations

a. 5 of the included studies were unclear risk of bias, 3 high risk of bias, and 1 low risk of bias

b. Results of interventions varied from significant to null effects.

c. 6 studies reported significant findings, with one finding non-significant difference and two did not report significance.

d. Results presented did not always include reporting of 95%CI and some included studies did not report comparative outcomes statistically between groups.

e. 6 of the included studies were unclear risk of bias, with 3 high risk of bias, and 1 low risk of bias

f. 7 studies reported significant findings, with one finding non-significant difference and two did not report significance

g. 6 out of 8 included studies demonstrated high risk of bias, the additional 2 were unclear risk of bias
