# Supplementary material for: Effectiveness of dietary interventions in individuals with diabetes for preventing and healing chronic wounds; a systematic review with meta‐analysis
Source: Diabet Med. 2025 Jul 9;42(9):e70100. doi: 10.1111/dme.70100 (PMC12352720; doi:10.1111/dme.70100)
Supplement: Supplementary file 1 — Data S1. [file DME-42-e70100-s001.zip › dme70100-sup-0005-Supinfo05..docx]

**Supplementary Material 5. Additional References**

51. Group TGW. GRADE. Accessed 13th March, 2023. https://www.gradeworkinggroup.org/

52. Afzali H, Jafari Kashi AH, Momen-Heravi M, et al. The effects of magnesium and vitamin E co-supplementation on wound healing and metabolic status in patients with diabetic foot ulcer: A randomized, double-blind, placebo-controlled trial. Randomized Controlled Trial

Research Support, Non-U.S. Gov't. *Wound Repair Regen*. 05 2019;27(3):277-284. doi:https://dx.doi.org/10.1111/wrr.12701

53. Armstrong DG, Hanft JR, Driver VR, et al. Effect of oral nutritional supplementation on wound healing in diabetic foot ulcers: a prospective randomized controlled trial. *Diabet Med*. Sep 2014;31(9):1069-77. doi:10.1111/dme.12509

54. Bashmakov YK, Assaad-Khali SH, Seif MA, et al. Resveratrol Promotes Foot Ulcer Size Reduction in Type 2 Diabetes Patients. *ISRN Endocrinology*. 2014:1-8. doi:10.1155/2014/816307

55. Basiri R, Spicer MT, Levenson CW, Ormsbee MJ, Ledermann T, Arjmandi BH. Nutritional Supplementation Concurrent with Nutrition Education Accelerates the Wound Healing Process in Patients with Diabetic Foot Ulcers. *Biomedicines*. Aug 3 2020;8(8)doi:10.3390/biomedicines8080263

56. Eneroth M, Larsson J, Oscarsson C, Apelqvist J. Nutritional supplementation for diabetic foot ulcers: the first RCT. Clinical Trial; Journal Article; Randomized Controlled Trial; Research Support, Non‐U.S. Gov't. *Journal of wound care*. 2004;13(6):230‐234. doi:10.12968/jowc.2004.13.6.26627

57. Gunton JE, Girgis CM, Lau T, Vicaretti M, Begg L, Flood V. Vitamin C improves healing of foot ulcers: a randomised, double-blind, placebo-controlled trial. *Br J Nutr*. Nov 28 2021;126(10):1451-1458. doi:10.1017/s0007114520003815

58. Halschou-Jensen PM, Sauer J, Bouchelouche P, Fabrin J, Brorson S, Ohrt-Nissen S. Improved Healing of Diabetic Foot Ulcers After High-dose Vitamin D: A Randomized Double-blinded Clinical Trial. *Int*. Jul 02 2021:15347346211020268. doi:https://dx.doi.org/10.1177/15347346211020268

59. Kamble A, Ambad RS, Padamwar M, Kakade A, Yeola M. To study the effect of oral vitamin d supplements on wound healing in patient with diabetic foot ulcer and its effect on lipid metabolism. *International Journal of Research in Pharmaceutical Sciences*. 2020;11(2):2701-2706. doi:http://dx.doi.org/10.26452/ijrps.v11i2.2290

60. Mohseni S, Bayani M, Bahmani F, et al. The beneficial effects of probiotic administration on wound healing and metabolic status in patients with diabetic foot ulcer: a randomized, double-blind, placebo-controlled trial. *Diabetes/Metabolism Research and Reviews*. 2017;(no pagination)doi:10.1002/dmrr.2970

61. Mokhtari M, Razzaghi R, Momen-Heravi M. The effects of curcumin intake on wound healing and metabolic status in patients with diabetic foot ulcer: A randomized, double-blind, placebo-controlled trial. *Phytother Res*. Apr 2021;35(4):2099-2107. doi:10.1002/ptr.6957

62. Momen-Heravi M, Barahimi E, Razzaghi R, Bahmani F, Gilasi HR, Asemi Z. The effects of zinc supplementation on wound healing and metabolic status in patients with diabetic foot ulcer: A randomized, double-blind, placebo-controlled trial. *Wound Repair Regen*. 2017;25(3):512-520. doi:https://dx.doi.org/10.1111/wrr.12537

63. Mozaffari-khosravi H, Haratian M, Moeintavakkoli H, Nadjarzadeh A. Comparative Effect of Two Different Doses of Vitamin D on Diabetic Foot Ulcer and Inflammatory Indices among the Type 2 Diabetic Patients: a Randomized Clinical Trial. 01/01 2016;8

64. Razzaghi R, Pidar F, Momen-Heravi M, Bahmani F, Akbari H, Asemi Z. Magnesium Supplementation and the Effects on Wound Healing and Metabolic Status in Patients with Diabetic Foot Ulcer: a Randomized, Double-Blind, Placebo-Controlled Trial. *Biol Trace Elem Res*. Feb 2018;181(2):207-215. doi:10.1007/s12011-017-1056-5

65. Soleimani Z, Hashemdokht F, Bahmani F, Taghizadeh M, Memarzadeh MR, Asemi Z. Clinical and metabolic response to flaxseed oil omega-3 fatty acids supplementation in patients with diabetic foot ulcer: A randomized, double-blind, placebo-controlled trial. Randomized Controlled Trial. *Journal of Diabetes & its Complications*. Sep 2017;31(9):1394-1400. doi:https://dx.doi.org/10.1016/j.jdiacomp.2017.06.010

66. Yanes-Quesada M, Navarro-Despaigne D, Cabrera-Rode E, et al. Effectiveness of Diamel® In the Treatment of Diabetic Foot Ulcers: A Randomized and Placebo-Controlled Phase II Clinical Trial. *J Diabetes Metab*. 2021;12:891. doi:10.35248/2155-6156.21.12.891

67. Yarahmadi A, Saeed Modaghegh M-H, Mostafavi-Pour Z, et al. The effect of platelet-rich plasma-fibrin glue dressing in combination with oral vitamin E and C for treatment of non-healing diabetic foot ulcers: a randomized, double-blind, parallel-group, clinical trial. Randomized Controlled Trial

Research Support, Non-U.S. Gov't. *Expert Opin Biol Ther*. 05 2021;21(5):687-696. doi:https://dx.doi.org/10.1080/14712598.2021.1897100

68. Bosede BE, Olubayo A, John A, et al. Ameliorative role of antioxidant micronutrients: Selenium, vitamins C and E on oxidative stress and wound healing in type 2 diabetic patients with foot ulcer in Ibadan. Article. *IIOAB Journal*. 2012;3(4):1-5.

69. Yang L, Zhou Z. Effects of nutrition intervention on the rehabilitation level and quality of life of patients with diabetes foot: Image observation based on image recognition technology. *Prev Med*. Aug 2023;173:107578. doi:10.1016/j.ypmed.2023.107578

70. Das AM, Sharan J, Biswas M. Role of Parenteral Amino Acid in Patients of Diabetic Foot Ulcer. *European Journal of Molecular and Clinical Medicine*. December 2022;9(3):2071-2079.

71. Rangabashyam SR, Ramalingam K, Nandhini K, Poovarasan M, Praveen Kumar V, Balasubramanian A. Research article a prospective study on effect of vitamin-d treatment in diabetic patients with chronic foot ulcer. Article. *International Journal of Pharmaceutical Research*. 2020;12(3):3014-3017. doi:10.31838/ijpr/2020.12.03.428

72. Sung JA, Gurung S, Lam T, et al. A 'Speed-Dating' Model of Wound Care? Rapid, High-Volume Assessment of Patients With Diabetes in a Multidisciplinary Foot Wound Clinic. *Exp Clin Endocrinol Diabetes*. May 7 2020;doi:10.1055/a-1151-4731

73. Kondrup J, Rasmussen H, Hamberg O, Stanga Z. Nutritional risk screening (NRS 2002): a new method based on an analysis of controlled clinical trials. *Clinical Nutrition*. 2003;22:321-36.

74. Tehan PE, Burrows T, Hawes MB, et al. Factors influencing diabetes-related foot ulcer healing in Australian adults: A prospective cohort study. *Diabet Med*. Jan 2023;40(1):e14951. doi:10.1111/dme.14951

75. Zinder R, Cooley R, Vlad LG, Molnar JA. Vitamin A and Wound Healing. *Nutr Clin Pract*. Dec 2019;34(6):839-849. doi:10.1002/ncp.10420

76. Aguilera JM. The food matrix: implications in processing, nutrition and health. *Critical Reviews in Food Science and Nutrition*. 2019/12/16 2019;59(22):3612-3629. doi:10.1080/10408398.2018.1502743

77. Bondonno NP, Bondonno CP, Ward NC, Hodgson JM, Croft KD. The cardiovascular health benefits of apples: Whole fruit vs. isolated compounds. *Trends in Food Science & Technology*. 2017/11/01/ 2017;69:243-256. doi:https://doi.org/10.1016/j.tifs.2017.04.012

78. O’Donnell TF, Jr., Passman MA, Marston WA, et al. Management of venous leg ulcers: Clinical practice guidelines of the Society for Vascular Surgery<sup>&#xae;</sup> and the American Venous Forum. *Journal of Vascular Surgery*. 2014;60(2):3S-59S. doi:10.1016/j.jvs.2014.04.049

79. Elia M, Ceriello A, Laube H, Sinclair AJ, Engfer M, Stratton RJ. Enteral Nutritional Support and Use of Diabetes-Specific Formulas for Patients With Diabetes: A systematic review and meta-analysis. *Diabetes Care*. 2005;28(9):2267-2279. doi:10.2337/diacare.28.9.2267

80. Champagne CM, Bray GA. Nutritional Status: An Overview of Methods for Assessment. In: Temple NJ, Wilson T, Bray GA, eds. *Nutrition Guide for Physicians and Related Healthcare Professionals*. Springer International Publishing; 2017:351-360.

81. Suresh S. Assessing nutritional status: Tools and techniques for nutritional assessment. *J Food Sci Nutr*. 2024;7(1):224.

82. National Health and Medical Research Council. Nutrient Reference Values for Australia and New Zealand. NHMRC. https://www.eatforhealth.gov.au/nutrient-reference-values/nutrients

83. Reber E, Gomes F, Vasiloglou MF, Schuetz P, Stanga Z. Nutritional Risk Screening and Assessment. *J Clin Med*. Jul 20 2019;8(7)doi:10.3390/jcm8071065

84. Deng L, Du C, Song P, et al. The Role of Oxidative Stress and Antioxidants in Diabetic Wound Healing. *Oxid Med Cell Longev*. 2021;2021:8852759. doi:10.1155/2021/8852759

85. Faghfouri AH, Zarezadeh M, Aghapour B, et al. Clinical efficacy of zinc supplementation in improving antioxidant defense system: A comprehensive systematic review and time-response meta-analysis of controlled clinical trials. *Eur J Pharmacol*. Sep 15 2021;907:174243. doi:10.1016/j.ejphar.2021.174243

86. Heshmati J, Farsi F, Shokri F, et al. A systematic review and meta-analysis of the probiotics and synbiotics effects on oxidative stress. *Journal of Functional Foods*. 2018/07/01/ 2018;46:66-84. doi:https://doi.org/10.1016/j.jff.2018.04.049

87. Heshmati J, Morvaridzadeh M, Maroufizadeh S, et al. Omega-3 fatty acids supplementation and oxidative stress parameters: A systematic review and meta-analysis of clinical trials. *Pharmacol Res*. Nov 2019;149:104462. doi:10.1016/j.phrs.2019.104462

88. Jakubczyk K, Drużga A, Katarzyna J, Skonieczna-Żydecka K. Antioxidant Potential of Curcumin-A Meta-Analysis of Randomized Clinical Trials. *Antioxidants (Basel)*. Nov 6 2020;9(11)doi:10.3390/antiox9111092

89. Silvestrini A, Meucci E, Ricerca BM, Mancini A. Total Antioxidant Capacity: Biochemical Aspects and Clinical Significance. *Int J Mol Sci*. Jul 1 2023;24(13)doi:10.3390/ijms241310978

90. Shivappa N, Steck SE, Hurley TG, Hussey JR, Hébert JR. Designing and developing a literature-derived, population-based dietary inflammatory index. *Public Health Nutr*. Aug 2014;17(8):1689-96. doi:10.1017/s1368980013002115

91. Fowler MJ. Microvascular and Macrovascular Complications of Diabetes. *Clinical Diabetes*. 2008;26(2):77-82. doi:10.2337/diaclin.26.2.77

92. Buse JB, Ginsberg HN, Bakris GL, et al. Primary Prevention of Cardiovascular Diseases in People With Diabetes Mellitus. *Circulation*. 2007;115(1):114-126. doi:doi:10.1161/CIRCULATIONAHA.106.179294

93. Ross LJ, Barnes KA, Ball LE, et al. Effectiveness of dietetic consultation for lowering blood lipid levels in the management of cardiovascular disease risk: A systematic review and meta-analysis of randomised controlled trials. *Nutr Diet*. Apr 2019;76(2):199-210. doi:10.1111/1747-0080.12509

94. Dudzik JM, Senkus KE, Evert AB, et al. The effectiveness of medical nutrition therapy provided by a dietitian in adults with prediabetes: a systematic review and meta-analysis. *The American Journal of Clinical Nutrition*. 2023;118(5):892-910. doi:10.1016/j.ajcnut.2023.08.022

95. Robertson S, Clarke ED, Gómez-Martín M, Cross V, Collins CE, Stanford J. Do Precision and Personalised Nutrition Interventions Improve Risk Factors in Adults with Prediabetes or Metabolic Syndrome? A Systematic Review of Randomised Controlled Trials. *Nutrients*. 2024;16(10):1479.
