# Supplementary material for: Effectiveness of dietary interventions in individuals with diabetes for preventing and healing chronic wounds; a systematic review with meta‐analysis
Source: Diabet Med. 2025 Jul 9;42(9):e70100. doi: 10.1111/dme.70100 (PMC12352720; doi:10.1111/dme.70100)
Supplement: Supplementary file 1 — Data S1. [file DME-42-e70100-s001.zip › dme70100-sup-0006-TableS1..docx]

| **Supplementary Table 1. Relevant trials derived from the trials databases. (n=37)** | | | |
| --- | --- | --- | --- |
| **Trial database** | **Trial registration No.** | **Title** | **Status** |
| ANZCTR | ACTRN12623001111662 | SENSATE trial: Smart Eating and Nutrition Supports solving Amputations, Toe loss and Exudate. Feasibility and acceptability of a personalised wound healing nutrition intervention in those living with diabetes-related foot ulcerations: a pilot randomised controlled trial | Recruiting |
| ANZCTR | ACTRN12624000675527p | FOOT-C: Foot Ulcer Treatment with vitamin C.  Efficacy of vitamin C treatment to aid the healing of foot ulcers in people with diabetes: a randomised, placebo-controlled double-blind trial | Not yet recruiting |
| ANZCTR | ACTRN12621000120875p | Blockade of mini-TrpRS for treatment of diabetic foot syndrome: A Prospective open-label phase 1a/1b randomized placebo-controlled trial | Ongoing |
| ISRCTN | 15570706 | REDUCE Trial: Reducing the impact of diabetic foot ulcers (DFUs)unsur | Recruiting |
| Clinical trials.gov | NCT05243368 | Evaluation of Personalized Nutritional Intervention on Wound Healing of Cutaneous Ulcers in Diabetics | Active, not recruiting |
| Clinical trials.gov | NCT05464407 | High Protein Oral Nutritional Support With Special Nutrients in Patients With Type 2 Diabetes and Foot Ulcer | Recruiting |
| Clinical trials.gov | NCT05308862 | PROSENIOR. Prevention of Pressure Ulcers, Malnutrition, Poor Oral Health and Falls Among Older Persons Receiving Municipal Health Care and Are Registered in the Quality Registry Senior Alert | Recruiting |
| Clinical trials.gov | NCT03576989 | Impact of Omega-3 Fatty Acid Oral Therapy on Healing of Chronic Venous Leg Ulcers in Older Adults | Recruiting |
| Clinical trials.gov | NCT04247451 | Diabetic Foot Surgery Patients: What is Their Metabolic Profile and Are Nutritional Goals Met (DIM-SUUM) | Recruiting |
| Clinical trials.gov | NCT03679273 | Nutritional Supplement on Wound Healing in Diabetic Foot.  Clinical Effectiveness and Molecular Mechanisms of Nutritional Supplement on Wound Healing in Diabetic Patients With Limb-threatening Foot Ulcer | Unknown. Recruitment status was recruiting |
| WHO | PACTR202106542254817 | Utility of Low Dose combination of Vitamin B (Fenomin) in the management of diabetic foot ulcer at the National Diabetes Management and Research Centre, Ghana | NR |
| WHO/ clinical trials.gov | NCT04370106 | The Impact of Frequenting a Social Leg Program on Therapeutic Adherence and Venous Leg Ulcer Wound Healing Outcomes (legclub) | Not yet recruiting |
| WHO/ clinical trials.gov | NCT04019340 | The Impact of a Pluridisciplinary Education Program on Venous Leg Ulcer Size Reduction (vened) | Recruiting |
| WHO/ clinical trials.gov | CTRI/2021/08/035385 | A study of the effects of Vitamin C and Vitamin E supplementation on the rate of wound healing in diabetic foot ulcers - A Randomized Controlled Trial | Not yet recruiting |
| WHO/ANZTCR | ACTRN12621001493831 | VITAFOOT- a pilot randomised controlled trial of vitamin supplementation on wound healing in people with diabetes-related foot ulcers | Not yet recruiting |
| ANZCTR | ACTRN12622001144707 | Vitamin C and zinc for healing of foot ulcers in people attending a high-risk foot service | Recruiting |
| WHO | PACTR202106542254817 | Fenomin and Diabetic foot ulcer: Utility of Low Dose combination of Vitamin B (Fenomin) in the management of diabetic foot ulcer at the National Diabetes Management and Research Centre, Ghana | Not yet recruiting |
| ANZCTR | 12613000142730 | Does Arginine supplementation need to be part of routine clinical education for Diabetes Educators in the management of foot ulcers | Not yet recruiting |
| CTRI | 19313 | Evaluation of efficacy of herbal lepa and vati in non-healing wound (Dushta vrana) and also its role in infection | Not yet recruiting |
| ANZCTR | ACTRN12612000036819 | The Effect of Nutritional Supplementation on the Healing of Diabetic Foot Ulcers | Completed however results not published |
| IRCT | IRCT2017040419669N3 | Effects of propolis on diabetic foot ulcer | Results not published |
| WHO | ACTRN12617001142325 | Vitamin C and healing of foot ulcers | Completed |
| Clinical trials.gov | NCT03813927 | Vitamin D Treatment of Diabetic Patients With Foot Ulcers | Completed |
| Clinical trials.gov | NCT04315909 | The Effect of Platelet-Rich Plasma-Fibrin Glue in Combination With Vitamin E and C for Treatment of Non-healing Diabetic Foot Ulcers | Completed |
| Clinical trials.gov | NCT04055064 | The Effects of Nutrition Supplementation and Education on the Healing of Diabetic Foot Ulcer (DFU) | Completed |
| WHO | IRCT201510315623N54 | Effects of vitamin D supplementation compared with the placebo on metabolic profiles, inflammatory factors and biomarkers of oxidative stress in patients with diabetic foot | Completed |
| WHO | IRCT201612225623N96 | Effect of supplementation in treatment of patients with diabetic foot ulcer | Completed |
| ANZCTR | ACTRN12612000036819 | The Effect of Nutritional Supplementation on the Healing of Diabetic Foot Ulcers | Completed |
| WHO | IRCT2017090533941N21 | Effect of combined magnesium and vitamin E supplementation in treatment of diabetic foot ulcer | Completed |
| WHO | IRCT201604025623N72 | Effect of supplementation in treatment of patients with diabetic foot | Completed |
| Clinical trials.gov | NCT00711217 | Evaluation of a Medical Food for Chronic Wound | Completed |
| Clinical trials.gov | NCT05281562 | Immunonutrition for Diabetic Foot Ulcers | Terminated (There are not enough patients at our institution meeting eligibility criteria to complete the study as intended.) |
| Clinical trials.gov | NCT03995407 | 100% Whey Protein Based Diet In Enhancing Pressure Ulcer Healing. | Terminated (Resourcing issues impacted by COVID-19 pandemic.) |
| Clinical trials.gov | NCT00502372 | Effect of an Oral Supplement Enriched in Amino Acids and the Leucine Metabolite B-hydroxy B-methylbutyrate (HMB) | Terminated (VA never granted approval following suspension of enrolment.) |
| Clinical trials.gov | NCT01350102 | The Relationship of Hemoglobin A1c and Diabetic Wound Healing | Terminated (study closed due to recruitment problems) |
| Clinical trials.gov | NCT03993990 | Effects of Empowerment-based Program on Post-discharge Glycemic Control, and Foot Ulcer | Withdrawn (Lack of financial resources) |
| Clinical trials.gov | NCT04475861 | Metabolomics and Wound Healing in Diabetes | Withdrawn (no participants enrolled) |
| Clinical trials.gov | NCT03995407 | 100% Whey Protein Based Diet In Enhancing Pressure Ulcer Healing | Terminated (Resourcing issues impacted by COVID-19 pandemic. |
| Clinical trials.gov | NCT01657318 | Olivamine-containing Products in the Management of Patients With Nonhealing Lower Extremity Ulcers | Terminated (challenges encountered in the methodology and concerns with the gold standard of treatment) |
| European Clinical Trials Register | EUCTR2007-005195-14 | A monocentre, randomised, double-blind, between-patient study to compare the effect of Vitamin D 300.000 IU orally vs placebo on bone metabolism, muscolar function, vascular system and infection in patients with diabetic foot at high cardiovascular and infections risks. | Prematurely ended - ?due to adverse events |
| Clinical trials.gov | NCT06255288 | Time to Heal (Wound, Healing, Dialogue, Nutrition) SNAK A Nurse-led Intervention Consisting of a Structured Dialogue, Patient and Relative Information, and Protein Supplement for Patients With Leg Ulcers in Clinical Practice: A Feasibility Study | Recruiting |
| Clinical trials.gov | NCT01657318 | Olivamine-containing Products in the Management of Patients With Nonhealing Lower Extremity Ulcers | Terminated (challenges in methodology and concerns regarding gold standard of treatment) |
| WHO | ITMCTR2200006700 | A clinical trial of Fuzheng Heying recipe in treatment of severe diabetic foot gangrene | Not yet recruiting |
| WHO | TCTR20230502001 | Golden Rice Cookies with red betle leaf: A Tasty and Effective Biomolecular Solution for Preventing Diabetic Foot Ulcers  Targeting MMP-9 with Golden Rice-based Cookies Containing Piper crocatum: A Novel Biomolecular Intervention for Preventing Diabetic Foot Ulcers | Pending (Not yet recruiting) |
| WHO | ISRCTN13413505 | MiFoot – reducing heart disease risk in those with a history of diabetic foot ulcers  A Multifactorial intervention to improve cardiovascular outcomes in adults with type 2 diabetes and current or previous diabetes-related Foot ulcers - randomised controlled trial (MiFoot RCT) | Ongoing |
| Clinical trials.gov | NCT06151769 | The Effect Pomegranate Seed Oil Prevention and Progression Pressure Ulcers in Intensive Care Patients | Not yet recruiting |
| WHO = World Health Organisation  ANZCTR = Australian New Zealand Clinical Trials Registry  IRCT = Iranian Clinical Trials  ISRCTN = International Standard Randomised Controlled Trial Number  NR = not reported | | | |
