# Supplementary material for: Effectiveness of dietary interventions in individuals with diabetes for preventing and healing chronic wounds; a systematic review with meta‐analysis
Source: Diabet Med. 2025 Jul 9;42(9):e70100. doi: 10.1111/dme.70100 (PMC12352720; doi:10.1111/dme.70100)
Supplement: Supplementary file 1 — Data S1. [file DME-42-e70100-s001.zip › dme70100-sup-0007-TableS2..docx]

| **Supplementary Table 2. Study characteristics.** | | | | | | | | | | | | | |
| --- | --- | --- | --- | --- | --- | --- | --- | --- | --- | --- | --- | --- | --- |
| **Reference,**  **Country** | **Study Design** | **Intervention** | **Duration** | **Final follow up** | **Control** | **Sample Size** | **Age (Years)**  **Mean +/- SD (range)** | **Sex (M:F)** | **Number with diabetes n(%) and diabetes type** | **Diabetes, mean years +/-SD(range Duration)** | **Wound duration, mean months (range)** | **Wound grading at baseline** | **How wound healing was measured (primary outcome)** |
| **Single nutrient supplement studies (n=13)** | | | | | | | | | | | | | |
| Bashmakov 2014,  Egypt | Placebo-controlled, examiner-blinded, parallel-group pilot RCT. | 50 mg of trans-resveratrol (t-RSV) capsules twice a day (morning and evening) after a meal with noncarbonated water. The t-RSV is a polyphenolic compound. A proprietary formulation of t-RSV (t-RSV-Lycosome) was used. | 60 days | 60 days | Placebo capsules which contained only inert excipients used in the resveratrol pill formulation twice a day over a 60-day time period. | Intervention  n =14 | 54.0+/- 10.1 (34-74) | 8:6 | No with diabetes: 14 (100%)  Type 2 | 15.0+/-6.9 | 18.2+/-17.1 | Neuroischemic: n=3  Neuropathic: n=11  Meggitt-Wagner’s scale, n  Garde 1: 9  Grade 2: 5 | Wound surface area, proportion of people healed, wound size change, and wound length, width and depth. |
|  |  |  |  |  |  | Control  n = 10 | 59.8+/- 6.6 (47-67) | 7:3 | No with diabetes: 10 (100%)  Type 2 | 15.2+/-9.5 | 15.0+/-11.5 | Neuroischemic: n=0  Neuropathic: n=10  Meggitt-Wagner’s scale, n  Grade 1: 4  Grade 2: 6 |  |
| Gunton 2021,  Australia | Pragmatic, investigator-initiated, double-blind, inactive-placebo-RCT. | Vitamin C 500 mg daily in a slow-release capsule provided by a pahrmacist. If vitamin C deficient after 28 days, they were dispensed both vitamin C and glucosam-  ine tablets for the second 28 days. If  baseline serum vitamin C normal, they continued their  original medication for a further 28 d so all  deficient people were offered treatment with vitamin C while  original treatment assignment remained blinded. | 56 days | NR however the paper reports participants were followed until complete ulcer healing, with the maximum days to complete healing for both groups being 190 days. | Placebo | Intervention  n=7 | 63.9+/-22.2 | 5:2 | No with diabetes: 4(57%)  Type 1 and 2 | NR | Median(range): 2.07(0.23-35.44)* | NR | Wound reduction and time to healing (days). |
|  |  |  |  |  |  | Control  n=9 | 57.7+/-13.8 | 9:0 | No with diabetes: 4(44%)  Type 1 and 2 | NR | Median(range): 2.07(0.92-11.05)* | NR |  |
| Halschou-Jensen 2021,  Denmark | Parallel, double-blinded RCT. | High dose Vitamin D3 (170µg (6800 IU/day)). | 48 weeks or until wound healing or surgical intervention. | 48 weeks or until wound healing or surgical intervention. | Low dose Vitamin D3 (20µg (800IU /day)) | Intervention  n=24 | 63.3+/-9.3 | 18:6 | No with diabetes: 24 (100%)  Type 1: 6(25.0)  Type 2 insulin dependent: 12(50.0)  Type 2 noninsulin dependent: 6(25.0) | NR | NR | NR | Proportion of wounds healed, wound reduction and wound area. |
|  |  |  |  |  |  | Control  n=24 | 64.5+/-11.4 | 22:2 | No with diabetes: 24 (100%)  Type 1: 4(17.4)  Type 2 insulin dependent: 10(43.5)  Type 2 noninsulin dependent: 9(39.1) | NR | NR | NR |  |
| Kamble 2020,  India | A single-centre prospective RCT. | Vitamin D (Cholecalciferol 60000 IU) sachet. | 12 weeks | 12 weeks | Provided standard care. | Intervention  n=30 | 60.2+/-9.3 | 25:5 | No with diabetes: 30(100%)  Type: NR | NR | NR | Meggitt-Wagner’s grade 2 or 3 | Wound surface area. |
|  |  |  |  |  |  | Control  n=30 | 59.7+/-8.4 | 23:7 | No with diabetes: 30(100%)  Type: NR | NR | NR | Meggitt-Wagner’s grade 2 or 3 |  |
| Mozaffari-Khosravi 2016,  Iran | RCT | 300,000IU of vitamin D (cholecalciferol) (half of 1 ml vitamin D ampule) through intramuscular injection once at start of intervention. | 4 weeks | 4 weeks | 150,000 IU of vitamin D (cholecalciferol) (half of 1 ml vitamin D ampule) through intramuscular injection once at start of intervention. | Intervention  n=25 | 57.46+/-8.68 | 14:11 | No with diabetes: 25(100%)  Type 2 | 11.29+/-3.95 | NR | Meggitt-Wagner’s Grade 1: 4 (16.7)  Grade 2: 20 (83.3) | Wound area |
|  |  |  |  |  |  | Control  n=25 | 56.52+/-7.61 | 16:9 | No with diabetes: 25(100%)  Type 2 | 11.3+/-3.77 | NR | Meggitt-Wagner’s  Grade 1: 6 (26.1)  Grade 2: 17 (73.9) |  |
| Rangabashyam 2020,  India | Quasi-experimental study. | Vitamin D with a standard dose of 2000 IU/day | 6 weeks | 6 weeks | No placebo given. | Intervention  n=50 | Most (n=36) belong to the range of 61-70 yrs followed by 51-60 yrs (30). | 53:47 | No with diabetes: 50(100%)  Type: 2 | NR | NR | NR | Wound depth, width, and length |
|  |  |  |  |  |  | Control  n=50 |  |  | No with diabetes: 50(100%)  Type: 2 | NR | NR | NR |  |
| Razzaghi 2017,  Iran | A prospective double-blind placebo-controlled RCT. | 50,000 IU vitamin D  supplements | 12 weeks | 12 weeks | Placebo | Intervention  n=30 | 59.6 +/- 8.2 | 22:8 | No with diabetes: 30(100%)  Type:NR | NR | NR | Meggitt-Wagner’s criteria: grade 3 | Wound depth, width, and length. |
|  |  |  |  |  |  | Control  n=30 | 58.6 +/- 8.6 | 22:8 | No with diabetes: 30(100%)  Type:NR | NR | NR | Meggitt-Wagner’s criteria: grade 3 |  |
| Jain 2012,  India | Quasi-experimental study. | Vitamin E (dose NR) | NR | 24 months | Insulin or OHAs only (standard care) | Intervention | | | | | | | Presence of DFU |
|  |  |  |  |  |  | Type 1 diabetes w/o complications (n=32) | >45 years | NR | No with diabetes: 32(100%)  Type 1 | 1-2 years | NR | NR |  |
|  |  |  |  |  |  | Type 2 diabetes w/o complications (n=32) | >45 years | NR | No with diabetes: 32(100%)  Type 2 | 1-2 years | NR | NR |  |
|  |  |  |  |  |  | Type 1 diabetes w/ complications (n=45) | >45 years | NR | No with diabetes: 45(100%)  Type 1 | 5-7 years | NR | NR |  |
|  |  |  |  |  |  | Type 2 diabetes w/ complications (n=45) | >45 years | NR | No with diabetes: 45(100%)  Type 2 | 5-7 years | NR | NR |  |
|  |  |  |  |  |  | Control | | | | | | |  |
|  |  |  |  |  |  | Type 1 diabetes w/o complications (n=32) | >45 years | NR | No with diabetes: 32(100%)  Type 1 | 1-2 years | NR | NR |  |
|  |  |  |  |  |  | Type 2 diabetes w/o complications (n=32) | >45 years | NR | No with diabetes: 32(100%)  Type 2 | 1-2 years | NR | NR |  |
|  |  |  |  |  |  | Type 1 diabetes w/ complications (n=45) | >45 years | NR | No with diabetes: 45(100%)  Type 1 | 5-7 years | NR | NR |  |
|  |  |  |  |  |  | Type 2 diabetes w/ complications (n=45) | >45 years | NR | No with diabetes: 45(100%)  Type 2 | 5-7 years | NR | NR |  |
| Mohseni 2018,  Iran | Double-blind, placebo-controlled RCT. | Probiotic capsule containing Lactobacillus acidophilus, Lactobacillus casei, Lactobacillus Fermentum, and Bifidobacterium bifidum (2 × 109 CFU/g each) | 12 weeks | 12 weeks | Placebo | Intervention  n=30 | 62.6+/-9.7 | 20:10 | No with diabetes: 30(100%)  Type: NR | NR | NR | Meggitt-Wagner’s criteria: grade 3 | Wound depth, width, and length. |
|  |  |  |  |  |  | Control  n=30 | 58.5+/-11.0 | 20:10 | No with diabetes: 30(100%)  Type: NR | NR | NR | Meggitt-Wagner’s criteria: grade 3 |  |
| Mokhtari 2020,  Iran | Double-blind, placebo-controlled RCT. | Nanocurcumin (80 mg/day) | 12 weeks | 12 weeks | Placebo | Intervention  n=30 | 57.4+/-11.7 | 24:6 | No with diabetes: 30(100%)  Type: NR | NR | NR | Meggitt-Wagner’s criteria: grade 3 | Wound depth, width, and length. |
|  |  |  |  |  |  | Control  n=30 | 55.8+/-9.4 | 25:5 | No with diabetes: 30(100%)  Type: NR | NR | NR | Meggitt-Wagner’s criteria: grade 3 |  |
| Momen-Heravi 2017,  Iran | Double-blind, placebo-controlled RCT. | 220 mg zinc sulfate supplements containing 50 mg elemental zinc daily. | 12 weeks | 12 weeks | Placebo | Intervention  n=30 | 58.3+/-8.6 | 21:9 | No with diabetes: 30(100%)  Type: NR | NR | NR | Meggitt-Wagner’s criteria: grade 3 | Wound depth, width, and length. |
|  |  |  |  |  |  | Control  n=30 | 60.0+/-10.0 | 21:9 | No with diabetes: 30(100%)  Type: NR | NR | NR | Wagner-Meggitt’s criteria: grade 3 |  |
| Razzaghi 2018,  Iran | A double-blind placebo-controlled RCT. | 50 mg/day magnesium supplements as magnesium oxide | 12 weeks | 12 weeks | Placebo | Intervention  n=35 | 60.1+/-11.1 | 22:13 | No with diabetes: 35(100%)  Type:NR | NR | NR | Wagner-Meggitt’s criteria: grade 3 | Wound depth, width, and length. |
|  |  |  |  |  |  | Control  n=35 | 59.0+/-10.1 | 24:11 | No with diabetes: 35(100%)  Type:NR | NR | NR | Wagner-Meggitt’s criteria: grade 3 |  |
| Soleimani 2017,  Iran | Double-blind placebo-controlled clinical RCT. | 1000 mg/day omega-3 fatty acid from flaxseed oil daily. | 12 weeks | 12 weeks | Placebo | Intervention  n=30 | 58.8+/-11.2 | 23:7 | No with diabetes: 30(100%  Type: NR | NR | 0.76+/-0.21* | Meggitt-Wagner’s criteria: grade 3 | Wound depth, width, and length. |
|  |  |  |  |  |  | Control  n=30 | 59.9+/-9.2 | 23:7 | No with diabetes: 30(100%  Type: NR | NR | 0.78+/-0.18* | Meggitt-Wagner’s criteria: grade 3 |  |
| **Multi-nutrient supplement studies (n=7)** | | | | | | | | | | | | | |
| Afzali 2019,  Iran | Double-blind, placebo-controlled RCT | ONS: 250 mg Mg oxide and 400 IU vitamin E daily. | 12 weeks | 12 weeks | Placebo | Intervention  n = 30 | 57.2+/-11.0 | 24:6 | No. with diabetes:  30 (100%)  Type: NR | NR | NR | Meggitt-Wagner’s criteria: grade 3 | Wound depth, width and length. |
|  |  |  |  |  |  | Control  n = 30 | 55.5+/-4.9 | 22:6 | No. with diabetes:  30 (100%)  Type: NR | NR | NR | Meggitt-Wagner’s criteria: grade 3 |  |
| Bosede 2012,  Nigeria | 3-arm Quasi -experimental study | Group B1: Supplemented 1000 mg ascorbic acid, 400 mg α-tocopherol and 100 μg Se, dissolved in 100 ml of vanilla flavoured milky beverage daily. | 16 weeks | 16 weeks | No placebo given. | Intervention  n = 50  (Group B1  n = 25  Group B2  n = 25) | 52.04+/-8.65 (40-60) | 27:23 | No with diabetes: 50 (100%)  Type 2 | NR | NR | Meggitt-Wagner’s scale, n  Grade 2: 50 | ABDEFS Tool  (Aetiology, Base, Discharge, Edge, Floor, Size) |
|  |  | Group B2: 100 ml of vanilla flavoured milky beverage only daily. (Placebo) |  |  |  | Control  n = 50 | NR (reports matched for age) | NR (reports matched for sex) | No with diabetes: 0 (0%)  Type – n/a | NR | NR | N/A |  |
| Yarahmadi 2021,  Iran | Double-blind, parallel-group clinical RCT. | Oral vitamin E (200IU/2 Day) and vitamin C (250IU/2Day) | 8 weeks | 8 weeks | Placebo | Intervention  n=13 | 55.62+/-9.68 | 9:4 | No with diabetes: 13(100%)  Type 1 and 2 | 11.54+/-7.27 | 10.44+/-9.29 | Meggitt-Wagner’s criteria: Wagner 1 or 2 | Proportion of people healed, wound area and wound width and length. |
|  |  |  |  |  |  | Control  n=12 | 59.58+/-9.24 | 9:3 | No with diabetes: 12(100%)  Type 1 and 2 | 12.25+/-8.04 | 11.03+/-8.70 | Meggitt-Wagner’s criteria: Wagner 1 or 2 |  |
| Das 2022,  India | Quasi-experimental study | Amino acids (200ml) was given intravenously on alternate days in addition to the diet they were taking previously. | 15 days | 15 days | Same diabetic diet as they were taking before. | Intervention  n = 30 | 46.67% aged 51-60 years. Minimum subjects were in the age group of 31-40 years followed by > 60 years. | More males than females. Does not specify number | No with diabetes: 30 (100%)  Type 2 | 9.73 | NR | NR | Asepsis wound scoring system and Southampton Scoring System. |
|  |  |  |  |  |  | Control  n= 30 | 60% aged b/n 51 -60 years.  Minimum subjects were in the age group of 31-40 years followed by > 60 years. |  | No with diabetes: 30 (100%)  Type 2 | 9.4 | NR | NR |  |
| Armstrong 2014,  USA, Europe and Taiwan | Prospective, double-blinded, multi-centre design RCT. | ONS packet: 79 kcal, 7 g L-arginine, 7 g L-glutamine and 1.5 g calcium HMB (providing 1.2 g HMB) dissolved in 237mL of water twice per day. | 16 weeks | 16 weeks | Placebo (Calorically similar (88 kcal), low glycaemic response supplement dissolved in 237mL of water twice per day.) | Intervention  n =129 | 58 (28-86) | 93:36 | No with diabetes: 129 (100%)  Type 1: 7(5.4)  Type 2: 122(94.6) | 13(0, 45) | 3 (1, 12) | At least one University of Texas grade 1A foot ulcer | Wound area, wound reduction and proportion of people healed. |
|  |  |  |  |  |  | Control  n =141 | 59 (29-88) | 111:30 | No with diabetes: 141 (100%)  Type 1: 17(12.1)  Type 2: 123(87.9) | 15(1, 50) | 3(1, 11) | At least one University of Texas grade 1A foot ulcer |  |
| Eneroth 2004,  Sweden | Prospective double-blind RCT. | Fortimel, an oral nutritional supplement with 1kcal/ml.  The supplement, offered in a choice of six flavours to increase concordance, was taken between meals. Authors were asked to specific which type of Fortimel oral nutrition supplement utilised however no response. | 6 months | 6 months | Placebo | Intervention  n=26 | 74 (59-88) | 19:7 | No with diabetes: 26 (100%)  NR | 16(1-51) | 5.75(0.92-23.01)* | Meggitt-Wagner’s grade 1 or 2 | Proportion of people healed. |
|  |  |  |  |  |  | Control n=27 | 75 (61-85) | 21:6 | No with diabetes: 27 (100%)  NR | 15 (1-41) | 5.06(0.92-24.16)* | Meggitt-Wagner’s grade 1 or 2 |  |
| Yanes-Quesada  2021,  Cuba | Double-blind placebo-controlled clinical RCT. | 2x 600mg capsules three times per day prior to main meals of Diamel® (trace elements, amino acids, vitamins, and lettuce and blueberry extracts) + conventional therapy (Treatment with insulin, hygiene/dietary guidelines, foot care and local ulcer treatment.) | 1 year | 1 year | Placebo + conventional therapy (Treatment with insulin, hygiene/dietary guidelines, foot care and local ulcer treatment.) | Intervention  n=50 | 64.6+/-9.4 | 30:20 | No with diabetes: 50 (100%)  Type 2 only | 18.9+/-6.1 | NR | Meggitt-Wagner’s grade 1-2 | Proportion of people healed. |
|  |  |  |  |  |  | Control  n=50 | 63.5+/-10.9 | 29:21 | No with diabetes: 50 (100%)  Type 2 only | 18.8+/-5.8 | NR | Meggitt-Wagner’s grade 1-2 |  |
| **Nutrition education (n=3)** | | | | | | | | | | | | | |
| Basiri 2020,  USA | Repeated measures RCT | Nutritional supplements concurrently with nutrition education from a dietitian (brief 5-10 minute education every 4 weeks) in addition to the standard-of-care regimen.  Two servings (474 mL) of a nutrient-dense formula, Boost Glucose Control, (Nestle), b/n meals—preferably one in morning b/n breakfast & lunch, & one in afternoon b/n lunch & dinner. | Until complete wound closure and/or up to 12 weeks. | Until complete wound closure and/or up to 12 weeks. | Standard-of-care regimen. | Intervention  n = 15 | 52.93+/-9.74  Median: 54  IQR: 12 | 8:7 | No with diabetes: 15 (100%)  Type: 1 and 2 | 14.40+/-8.03  Median: 12  IQR: 10 | 10.97+/-15.09  Median: 6  IQR: 10 | At least one University of Texas grade 1 or 2 stage A foot ulcer | Proportion of people healed and wound reduction. |
|  |  |  |  |  |  | Control  n = 14 | 53.79+/-12.84  Median: 55  IQR: 21 | 11:3 | No with diabetes: 14 (100%)  Type 1 and 2 | 11.71+/-6.17  Median: 12  IQR: 12 | 10.58+/-18.27  Median: 6  IQR:9.5 | At least one University of Texas grade 1 or 2 stage A foot ulcer |  |
| Sung 2021,  Australia | A retrospective case-control study | MDT  Dietitian, diabetes educator, clinical psychologist and endocrinologist consultation in addition to standard wound care. | Trial ran over 6 months however how many times someone saw the MDT was not specified. | Trial ran over 6 months however how many times someone saw the MDT was not specified. | Standard wound care. | Intervention  n=55 | 62.9+/-12.7 | 39:16 | No with diabetes: 55(100%)  Type 1: n= 2  Type 2: n= 53 | 16.3+/-7.3(n=54) | NR | NR | Proportion of wounds healed and wound volume. |
|  |  |  |  |  |  | Control  n=64 | 66.0+/-13.1 | 41:23 | No with diabetes: 64 (100%)  Type 1: n= 1  Type 2: n= 63 | 16.2+/-8.9 (n=43) | NR | NR |  |
| Yang 2023,  China | Case-control | Early nurse-led nutrition intervention based on NRS2002 system.  NRS2002 score of <3: control proportion of carbohydrates, increase protein intake, limit fat, and increase dietary fibre, vitamin and mineral intake.  NRS 2002 score of ≥3: more personalised based on patient’s metabolism and physical condition. | NR | NR | Routine nursing intervention which included: nutrition education, guiding patients to control dietary calories, maintain or slightly lower their weight, and control the intake of sugar, fat and salt, and increase protein, dietary fibre and water intake. Also education on sufficient sleep and moderate exercise. | Intervention  n = 100 | NR | NR | NR | NR | NR | NR | Proportion of people healed/improved. |
|  |  |  |  |  |  | Control  n = 100 | NR | NR | NR | NR | NR |  |  |
| *the wound duration was changed from weeks to months for this study  M:F= Male to Female ratio  NR = not reported  w/ = with  w/o = without  b/n = between  & = and  OHAs = oral hypoglycaemic agents  MDT = multidisciplinary team  HMB = beta-hydroxy-beta-methylbutyrate  DFU = Diabetes-related foot ulcer  Mg = Magnesium | | | | | | | | | | | | | |
