# Supplementary material for: Effectiveness of dietary interventions in individuals with diabetes for preventing and healing chronic wounds; a systematic review with meta‐analysis
Source: Diabet Med. 2025 Jul 9;42(9):e70100. doi: 10.1111/dme.70100 (PMC12352720; doi:10.1111/dme.70100)
Supplement: Supplementary file 1 — Data S1. [file DME-42-e70100-s001.zip › dme70100-sup-0008-TableS3.docx]

| **Supplementary Table 3. Wound healing outcome results including between-group difference reported in studies investigating the effectiveness of nutrition interventions for individuals with diabetes-related foot ulceration.** | | | | | | | | | | | | | | | | | | | |
| --- | --- | --- | --- | --- | --- | --- | --- | --- | --- | --- | --- | --- | --- | --- | --- | --- | --- | --- | --- |
| **Reference, country, intervention** | **Proportion of people healed (n=7)** | **Proportion of people healed between group difference at follow-up** | **Proportion of wounds healed (n=2)** | **Proportion of wounds healed between group difference at follow-up** | **Wound reduction (n=5)** | **Wound reduction between group difference at follow-up** | **Wound Metrics (n=6)** | **Wound Metrics between group difference at follow-up** | **Wound depth (n=9)** | **Wound depth between group difference at follow-up** | **Wound width (n=10)** | **Wound width between group difference at follow-up** | **Wound length (n=10)** | **Wound length between group difference at follow-up** | **Other (n=4)** | | **Other between group difference at follow-up** | |  |
| **Single nutrient supplement studies (n=13)** | | | | | | | | | | | | | | | | | | | |
| Bashmakov 2014,  Egypt, Trans-resveratrol | People with complete wound closure  After 30 days  Control: 0/10 (0%)  Intervention: 2/14 (14%)  After 60 days  Control: 1/10 (10%)  Intervention: 5/14 (35.7%)  The positive dynamics in the wound healing was also reflected by some reduction in the wound severity assessed by the Wagner scale. Such a reduction either from grade 2 to grade 1 or to complete wound closure took place in 8 out of 14 of the RSV-treated patients versus 3 out of 10 patients in the placebo group. | After 30 days  14% difference. NS (p=0.2169, 95%CI -0.32, 0.04)*  After 60 days  25.7% difference. NS (p=0.1517, 95%CI -0.57, 0.06)* | NR | NR | NR | NR | **Wound size (cm)**  Change after 4 weeks  Control: 0.98  Intervention: 1.96  Change after 8 weeks  Control: 2.10  Intervention: 3.13  **Wound area (cm^2^)**  Median  **Control**  pre-treatment: 4.25 cm2  30^th^ day: 2.75 cm2 (95% CI: 1.00, 12.59) (SD 9.35*)  60^th^ day: 2.13 cm2 (95% CI: 0.66, 7.38) (SD 5.42*)  **Intervention**  Pre-treatment: 2.63 cm2  30^th^ day: 0.75 cm2 (95% CI: 0.23, 3.50) (SD 3.12*)  60^th^ day: 0.13 cm2 (95% CI: 0.00, 1.08) (SD 1.03*) | **Wound size**  Change after 4 weeks: Significant ↓ favouring intervention (p=0.0335, 95%CI -1.88, -0.08)  Change after 8 weeks:  NS difference (p=0.2059, 95%CI -2.67, 0.61)  **Wound area**  30th day: NS difference (p=0.4614, 95%CI -3.53, 7.53*)  60^th^ day:  NS difference (p=0.1881, 95%CI -1.05, 5.05*) | **Control**  Baseline mean (SD): 0.9 (0.7)  Mean change after 4 weeks: 0.02 (SD 0.05) (95%CI −0.01, 0.05)  Mean change after 8 weeks: 0.20 (SD 0.28*) (95%CI 0.02, 0.37)  **Intervention**  Baseline mean (SD): 0.6 (0.2)  Mean change after 4 weeks: 0.23 (SD 0.22*) (95%CI 0.11, 0.34)  Mean change after 8 weeks: 0.35 (SD 0.25*) (95%CI 0.22, 0.48) | After 4 weeks: significant ↓ favouring intervention (p=0.0075, 95%CI -0.36, -0.06)*  After 8 weeks: NS difference (p=0.1817, 95%CI -0.38, 0.08)* | **Control**  Baseline mean (SD): 2.7 (1.7)  Mean change after 4 weeks: 0.56 (SD 0.42*) (95%CI 0.30, 0.82)  Mean change after 8 weeks: 1.00 (SD 0.90*) (95%CI 0.44, 1.56)  **Intervention**  Baseline mean (SD): 1.9 (1.3)  Mean change after 4 weeks: 0.84 (SD 0.65*) (95%CI 0.50, 1.18)  Mean change after 8 weeks: 1.23 (SD 0.73*) (95%CI 0.85, 1.61) | After 4 weeks: NS difference (p=0.2459, 95%CI -0.77, 0.21)*  After 8 weeks: NS difference (p=0.4968, 95%CI -0.92, 0.46)* | **Control**  Baseline mean (SD): 2.9 (1.9)  Mean change after 4 weeks: 0.40 (SD 0.37*) (95%CI 0.17, 0.63)  Mean change after 8 weeks: 0.90 (SD 0.86*) (95%CI 0.37, 1.43)  **Intervention**  Baseline mean (SD): 2.7 (2.6)  Mean change after 4 weeks: 0.89 (SD 0.81*) (95%CI 0.47, 1.32)  Mean change after 8 weeks: 1.55 (SD 1.24*) (95%CI 0.90, 2.20) | After 4 weeks: NS difference (p=0.0895, 95%CI -1.06, 0.08)*  After 8 weeks: NS difference (p=0.1678, 95%CI -1.59, 0.29)* | NR | | NR | |  |
| Gunton 2021,  Australia, Vitamin C | NR | NR | NR | NR | **Percentage reduction in ulcer volume at 8 weeks**  **Control**  Median -14%  **Intervention**  Median 100% | Significant ↓ favouring intervention (p= 0.041) | NR | NR | NR | NR | NR | NR | NR | NR | **Median time to 50% ulcer healing**  **Control**  Median 48 days  **Intervention**  Median 20 days  **Time to complete ulcer healing n, median days (day range)**  **Control**  5/9, 77 days (26-146)  **Intervention**  7/7, 77 days (21-190) | | Time to 50% ulcer healing: Significant ↓ favouring intervention (p=0.028) | |  |
| Halschou-Jensen 2021,  Denmark, Vitamin D | NR | NR | No. wounds healed within 48 weeks  Control:  Intention-to-treat analysis: 12/34 (35%)  Per-protocol analysis: 11/29 (37%)  Intervention:  Intention-to-treat analysis: 21/30(70%)  Per-protocol analysis: 20/28 (71%) | Intention-to-treat analysis: Significant ↑ favouring intervention (p=0.012)  Per-protocol analysis: Significant ↑ favouring intervention (p=0.023). | **Median ulceration reduction at final follow-up**  **Control:** 57%(IQR: -28 to 100)  **Intervention:** 100%(IQR: 72-100) | Significant ↓ favouring intervention (p=0.0008, 95%CI -67.12, -18.88)* | **Wound area (cm^2^)**  **Control median [IQR] (min, max)**  Baseline: 2.1[0.8-5.0](0.2, 20.8)  End of treatment: 1.0[0.0-3.1](0.0, 18.6)  **Intervention median [IQR] (min, max)**  Baseline: 1.25[0.4-3.2](0.1, 12.4)  End of treatment: 0[0.0-0.8](0.0, 11.7) | Significant ↓ favouring intervention (p=0.018) | NR | NR | NR | NR | NR | NR | NR | | NR | |  |
| Kamble 2020,  India, Vitamin D | NR | NR | NR | NR | NR | NR | **Wound surface area (units NR)**  Control change from baseline to follow up in 12 weeks mean +/- SD: 3.76+/-1.73  Intervention change from baseline to follow up in 12 weeks mean +/-SD: 8.06+/-6.82 | Significant ↓ favouring intervention (p=0.0014) | NR | NR | NR | NR | NR | NR | NR | | NR | |  |
| Mozaffari-Khosravi 2016,  Iran, Vitamin D | NR | NR | NR | NR | NR | NR | **Wound area (cm)**  **Control**  Baseline: 8.2+/-1.38  Week 4: 5.84+/-0.97  Change: -2.3+/-0.6  **Intervention**  Baseline: 7.92+/-1.9  Week 4: 5.23+/-1.29  Change: -2.8+/-1.0 | Baseline  NS difference (p=0.22)  Week 4  NS difference (p=0.07)  Change  NS difference (p=0.05) | NR | NR | NR | NR | NR | NR | NR | | NR | |  |
| Rangabashyam 2020,  India, Vitamin D | NR | NR | NR | NR | NR | NR | NR | NR | **Mean+/-SD**  **Week 1:**  Control: 1.29+/-0.39  Intervention: 1.57+/-0.34  **Week 2:**  Control: 1.26+/-0.38  Intervention: 1.39+/-0.34  **Week 3:**  Control: 1.16+/-0.39  Intervention: 1.12+/-0.33  **Week 4:**  Control: 1.08+/-0.39  Intervention: 1.08+/-0.32  **Week 5:**  Control: 0.97+/-0.41  Intervention: 0.99+/-0.24  **Week 6:**  Control: 0.86+/-0.42  Intervention: 0.76+/-0.14  Control: Decreased by 33.3%.  Intervention: Significant decrease by 77.0%. | **Week 2:** NS difference (p=0.0745, 95%CI -0.27, 0.01)*  **Week 3:** NS difference (p=0.5811, 95%CI -0.10, 0.18)*  **Week 4:** NS difference (p=1.0000, 95%CI -0.14, 0.14)*  **Week 5:** NS difference (p=0.7666, 95%CI -0.15, 0.11)*  **Week 6:** NS difference (p=0.1134, 95%CI -0.02, 0.22)* | **Mean+/-SD**  **Week 1:**  Control: 3.01+/-1.25  Intervention: 3.35+/-0.66  **Week 2:**  Control: 2.98+/-1.27  Intervention: 3.18+/-0.64  **Week 3:**  Control: 2.90+/-1.29  Intervention: 2.90+/-0.65  **Week 4:**  Control: 2.80+/-1.29  Intervention: 2.55+/-0.65  **Week 5:**  Control: 2.6+/-1.30  Intervention: 2.19+/-0.65  **Week 6:**  Control: 2.56+/-1.31  Intervention: 2.09+/-0.66  Control: Decreased by 14.95%.  Intervention: Significant decrease by 46.5%. | **Week 2:** NS difference (p=0.3225, 95%CI -0.60, 0.20)*  **Week 3**: NS difference (p=1.0000, 95%CI -0.41, 0.41)*  **Week 4:** NS difference (p=0.2240, 95%CI -0.16, 0.66)*  **Week 5:** significant ↓ favouring intervention (p=0.0489, 95%CI 0.00, 0.82)*  **Week 6:** significant ↓ favouring intervention (p=0.0257, 95%CI 0.06, 0.88)* | **Mean+/-SD**  **Week 1:**  Control: 3.44+/-1.46  Intervention: 4.1+/-1.15  **Week 2:**  Control: 3.40+/-1.50  Intervention: 3.9+/-1.13  **Week 3:**  Control: 3.37+/-1.52  Intervention: 3.60+/-1.11  **Week 4:**  Control: 3.30+/-1.54  Intervention: 3.55+/-1.11  **Week 5:**  Control: 3.18+/-1.55  Intervention: 3.29+/-1.12  **Week 6:**  Control: 3.08+/-1.56  Intervention: 3.1+/-1.11  Control: Decreased by 10.46%.  Intervention: Significant decrease by 27.7%. | **Week 2**: NS difference (p=0.0627, 95%CI -1.03, 0.03)*  **Week 3:** NS difference (p=0.3896, 95%CI -0.76, 0.30)*  **Week 4:** NS difference (p=0.3540, 95%CI -0.78, 0.28)*  **Week 5:** NS difference (p=0.6851, 95%CI -0.65, 0.43)*  **Week 6:** NS difference (p=0.9413, 95%CI -0.56, 0.52)* | NR | | NR | |  |
| Razzaghi 2017,  Iran, Vitamin D | NR | NR | NR | NR | NR | NR | NR | NR | **Non-adjusted mean +/-n SD**  **Control**  Change: -0.5+/-0.5  **Intervention**  Change: -1.0+/-0.5  **Adjusted^a^**  Control change from baseline to follow up in 12 weeks mean+/-SE: -0.5 +/- 0.1  Intervention change from baseline to follow up in 12 weeks mean+/-SE: -1.0 +/- 0.1 | Non-adjusted  Significant ↓ favouring intervention (p<0.001)  Adjusted  Significant ↓ favouring intervention (p<0.001) | **Non-adjusted mean +/-n SD**  **Control**  Change: -1.1+/-1.0  **Intervention**  Change: -2.0+/-1.2  **Adjusted**  Control change from baseline to follow up in 12 weeks mean+/-SE: -1.1 +/- 0.2  Intervention change from baseline to follow up in 12 weeks mean+/-SE: -1.9 +/- 0.2 | Non-adjusted  Significant ↓ favouring intervention (p=0.02)  Adjusted  Significant ↓ favouring intervention (p=0.01) | **Non-adjusted mean +/-n SD**  **Control**  Change: -1.1+/-1.1  **Intervention**  Change: -2.1+/-1.1  **Adjusted**  Control change from baseline to follow up in 12 weeks mean+/-SE: -1.1 +/- 0.2  Intervention change from baseline to follow up in 12 weeks mean+/-SE: -2.1 +/- 0.2 | Non-adjusted  Significant ↓ favouring intervention (p=0.001)  Adjusted  Significant ↓ favouring intervention (p=0.001) | NR | | NR | |  |
| Jain 2012,  India, Vitamin E | NR | NR | NR | NR | NR | NR | NR | NR | NR | NR | NR | NR | NR | NR | **18 months, n(%)**  Secondary prevention (with complications)  Type 1 diabetes w/ complications control group: 4 (12.5)  Type 1 diabetes w/ complications intervention group: 8 (25)  Type 2 diabetes w/ complications control group: 5 (15.63)  Type 2 diabetes w/ complications intervention group: 9 (28.13)  **24 months, n(%)**  Primary prevention (w/o complications)  Type 1 diabetes (w/o complications) control group: 12 (37.5)  Type 1 diabetes (w/o complications) intervention group: 8 (25)  Type 2 diabetes w/o complications) control group: 11 (34.38)  Type 2 diabetes (w/o complications) intervention group: 7 (21.88)  Secondary prevention  Type 1 diabetes w/ complications control group: 6 (18.8)  Type 1 diabetes w/ complications intervention group: 13 (40.63) *  Type 2 diabetes w/ complications control group: 7 (21.88)  Type 2 diabetes w/ complications intervention group: 16 (50) * | | **18 months**  Secondary prevention (with complications)  Type 1 diabetes w/ complications: NS difference (p=0.1287, 95%CI -0.28, 0.03)*  Type 2 diabetes w/ complications: NS difference (p=0.1515, 95%CI -0.29, 0.04)*  **24 months**  Primary prevention (w/o complications)  Type 1 diabetes w/o complications: NS difference (p=0.2807, 95%CI -0.10, 0.35)*  Type 2 diabetes w/o complications: NS difference (p=0.2661, 95%CI -0.09, 0.34)*  Secondary prevention  Type 1 diabetes w/ complications: Significant ↑ favouring intervention(p=0.0235, 95%CI -0.40, -0.03)*  Type 2 diabetes w/ complications: Significant ↑ favouring intervention(p=0.0054, 95%CI -0.47, -0.09)* | |  |
| Mohseni 2018,  Iran, Probiotic | NR | NR | NR | NR | NR | NR | NR | NR | **Non-adjusted mean +/-n SD**  **Control**  Baseline: 1.1+/-0.6  12 weeks: 0.8+/-0.5  Change: -0.3+/-0.3  **Intervention**  Baseline: 1.2+/-0.5  12 weeks: 0.7+/-0.5  Change: -0.5+/-0.3  **Adjusted^a^**  Control change from baseline to follow up in 12 weeks mean+/-SE: -0.3+/-0.1  Intervention change from baseline to follow up in 12 weeks mean+/-SE: -0.5+/-0.1 | Non-adjusted  Significant ↓ favouring intervention (p=0.02)  Adjusted Significant ↓ favouring intervention (p=0.02) | **Non-adjusted mean +/-n SD**  **Control**  Baseline: 2.6+/-1.3  12 weeks: 1.9+/-1.4  Change: -0.7+/-0.7  **Intervention**  Baseline: 2.4+/-1.3  12 weeks: 1.3+/-0.9  Change: -1.1+/-0.7  **Adjusted^a^**  Control change from baseline to follow up in 12 weeks mean+/-SE: -0.6+/-0.1  Intervention change from baseline to follow up in 12 weeks mean+/-SE: -1.1+/-0.1 | Non-adjusted Significant ↓ favouring intervention (p=0.02)  Adjusted Significant ↓ favouring intervention (p=0.01) | **Non-adjusted mean +/-n SD**  **Control**  Baseline: 3.2+/-1.8  12 weeks: 2.4+/-1.9  Change: -0.8+/-0.7  **Intervention**  Baseline: 3.2+/-1.7  12 weeks: 1.9+/-1.2  Change: -1.3+/-0.9  **Adjusted^a^**  Control change from baseline to follow up in 12 weeks mean+/-SE: -0.8+/-0.1  Intervention change from baseline to follow up in 12 weeks mean+/-SE: -1.3+/-0.1 | Non-adjusted  Significant ↓ favouring intervention (p=0.01)  Adjusted  Significant ↓ favouring intervention (p=0.007) | NR | | NR | |  |
| Mokhtari 2020,  Iran, Nanocurcumin | NR | NR | NR | NR | NR | NR | NR | NR | **Adjusted^b^**  **Baseline mean +/-SD**  Control: 1.5+/-0.7  Intervention: 1.5+/-0.5  **Week 12 mean +/-SD**  Control: 0.6+/-0.3  Intervention: 0.6+/-0.3 | NS difference (p=0.94)  B(95%CI): -0.003(-0.08, 0.08) | **Adjusted^b^**  **Baseline mean +/-SD**  Control: 1.6+/-0.9 Intervention: 1.8+/-0.9  **Week 12 mean +/-SD**  Control: 0.8+/-0.4  Intervention: 0.8+/-0.5 | NS difference (p=0.20)  B(95%CI): -0.07(-0.18, 0.04) | **Adjusted^b^**  **Baseline mean +/-SD**  Control: 2.0+/-1.0  intervention: 2.3+/-1.3  **Week 12 mean +/-SD**  Control: 0.9+/-0.5  Intervention: 1.2+/-0.8 | NS difference (p=0.64)  B(95%CI): 0.03(-0.11, 0.17) | NR | | NR | |  |
| Momen-Heravi 2017,  Iran, Zinc | NR | NR | NR | NR | NR | NR | NR | NR | **Non-adjusted mean +/-n SD**  **Control**  Baseline: 1.3+/-0.6  12 weeks: 1.0+/-0.9  Change: -0.3+/-1.0  **Intervention**  Baseline: 1.3+/-0.5  12 weeks: 0.5+/-0.5  Change: -0.8+/-0.6  **Adjusted^a^**  Control change from baseline to follow up in 12 weeks mean+/-SE: -0.3+/-0.1  Intervention change from baseline to follow up in 12 weeks: -0.7+/-0.1 | Non-adjusted  NS difference (p=0.05)  Adjusted  Significant ↓ favouring intervention (p=0.04) | **Non-adjusted mean +/-n SD**  **Control**  Baseline: 2.7+/-1.4  12 weeks: 1.9+/-1.0  Change: -0.8+/-1.0  **Intervention**  Baseline: 2.9+/-1.2  12 weeks: 1.5+/-1.3  Change: -1.4+/-0.8  **Adjusted^a^**  Control change from baseline to follow up in 12 weeks: -0.9+/-0.1  Intervention change from baseline to follow up in 12 weeks: -1.3+/-0.1 | Non-adjusted  Significant ↓ favouring intervention (p=0.02)  Adjusted Significant ↓ favouring intervention (p=0.02) | **Non-adjusted mean +/-n SD**  **Control**  Baseline: 3.1+/-1.6  12 weeks: 2.2+/-1.6  Change: -0.9+/-1.2  **Intervention**  Baseline: 3.1+/-1.5  12 weeks: 1.6+/-1.5  Change: -1.5+/-0.7  **Adjusted^a^**  Control change from baseline to follow up in 12 weeks: -0.9+/-0.2  Intervention change from baseline to follow up in 12 weeks: -1.5+/-0.2 | Non-adjusted  Significant ↓ favouring intervention (p=0.02)  Adjusted  Significant ↓ favouring intervention (p=0.02) | NR | | NR | |  |
| Razzaghi 2018,  Iran, Magnesium | NR | NR | NR | NR | NR | NR | NR | NR | **Non-adjusted mean +/-n SD**  **Control**  Baseline: 1.3+/-0.6  12 weeks: 0.9+/-0.5  Change: -0.3+/-0.5  **Intervention**  Baseline: 1.7+/-1.1  12 weeks: 0.9+/-1.4  Change: -0.8+/-0.8  **Adjusted^a^**  Control change from baseline to follow up in 12 weeks mean+/-SE: -0.4+/-0.1  Intervention change from baseline to follow up in 12 weeks mean+/-SE: -0.8+/-0.1 | Non-adjusted  Significant ↓ favouring intervention (p=0.003)  Adjusted Significant ↓ favouring intervention (p=0.005) | **Non-adjusted mean +/-n SD**  **Control**  Baseline: 2.9+/-1.4  12 weeks: 2.1+/-1.5  Change: -0.8+/-0.9  **Intervention**  Baseline: 3.3+/-2.8  12 weeks: 1.7+/-2.9  Change: -1.6+/-2.0  **Adjusted**  Control change from baseline to follow up in 12 weeks mean+/-SE: -0.8+/-0.2  Intervention change from baseline to follow up in 12 weeks mean+/-SE: -1.6+/-0.2 | Non-adjusted  Significant ↓ favouring intervention (p=0.02)  Adjusted  Significant ↓ favouring intervention (p=0.04) | **Non-adjusted mean +/-n SD**  **Control**  Baseline: 3.6+/-1.6  12 weeks: 2.7+/-1.9  Change: -0.9+/-1.1  **Intervention**  Baseline: 3.6+/-2.7  12 weeks: 1.8+/-2.9  Change: -1.8+/-2.0  **Adjusted^a^**  Control change from baseline to follow up in 12 weeks mean+/-SE: -0.9+/-0.3  Intervention change from baseline to follow up in 12 weeks mean+/-SE: -1.8+/-0.3 | Non-adjusted  Significant ↓ favouring intervention (p=0.01)  Adjusted  Significant ↓ favouring intervention (p=0.02) | NR | | NR | |  |
| Soleimani 2017,  Iran, Omega-3 | NR | NR | NR | NR | NR | NR | NR | NR | **Non-adjusted mean +/-n SD**  **Control**  Baseline: 1.3+/-0.6  12 weeks: 0.8+/-0.5  Change: -0.5+/-0.5  **Intervention**  Baseline: 1.4+/-0.5  12 weeks: 0.5+/-0.4  Change: -0.8+/-0.6  **Adjusted^a^**  Control change from baseline to follow up in 12 weeks mean+/-SE: -0.5+/-0.1  Intervention change from baseline to follow up in 12 weeks mean+/-SE: -0.8+/-0.1 | Non-adjusted  Significant ↓ favouring intervention (p=0.01)  Adjusted  Significant ↓ favouring intervention (p=0.008) | **Non-adjusted mean +/-n SD**  **Control**  Baseline: 2.9+/-1.3  12 weeks: 1.9+/-1.4  Change: -1.0+/-1.0  **Intervention**  Baseline: 2.9+/-2.1  12 weeks: 1.1+/-1.0  Change: -1.8+/-1.7  **Adjusted**  Control change from baseline to follow up in 12 weeks mean+/-SE: -1.0+/-0.2  Intervention change from baseline to follow up in 12 weeks mean+/-SE: -1.8+/-0.2 | Non-adjusted  Significant ↓ favouring intervention (p=0.02)  Adjusted  Significant ↓ favouring intervention (p=0.004) | **Non-adjusted mean +/-n SD**  **Control**  Baseline: 3.4+/-1.7  12 weeks: 2.4+/-1.8  Change: -1.0+/-1.1  **Intervention**  Baseline: 3.5+/-2.3  12 weeks: 1.4+/-1.0  Change: -2.1+/-2.3  **Adjusted**  Control change from baseline to follow up in 12 weeks mean+/-SE: -1.1+/-0.2  Intervention change from baseline to follow up in 12 weeks mean+/-SE: -2.0+/-0.2 | Non-adjusted  Significant ↓ favouring intervention (p=0.03)  Adjusted  Significant ↓ favouring intervention (p=0.006) | NR | | NR | |  |
| **Multi-nutrient supplement studies (n=7)** | | | | | | | | | | | | | | | |  | |  | |
| Afzali 2019,  Iran, Mg and vitamin E | NR | NR | NR | NR | NR | NR | NR | NR | **Adjusted^a^**  **Baseline mean +/-SD**  Control: 1.1+/-0.5  Intervention: 0.9+/-0.4  **Week 12 mean +/- SD**  Control: 0.9+/-0.5  Intervention: 0.4+/-0.3 | Significant ↓ favouring intervention (p=0.02)  B(95%CI): -0.18(-0.33,-0.02) | **Adjusted^a^**  **Baseline mean +/-SD**  Control: 2.5+/-0.7  Intervention: 2.1+/-1.3  **Week 12 mean +/-SD**  Control: 1.8+/-1.0  Intervention: 1.2+/-0.9 | Significant ↓ favouring intervention (p=0.02)  B(95%CI): -0.35 (-0.64, -0.05) | **Adjusted^a^**  **Baseline mean +/-SD**  Control: 3.1+/-1.1  Intervention: 2.8+/-1.3  **Week 12 mean +/-SD**  Control: 2.3+/-1.3  Intervention: 1.6+/-1.1 | Significant ↓ favouring intervention (p=0.003)  B(95%CI): -0.56 (-0.92, -0.20) | NR | | NR | |  |
| Bosede 2012,  Nigeria, Vitamin E, C and selenium | NR | NR | NR | NR | NR | NR | NR | NR | NR | NR | NR | NR | NR | NR | **ABDEFS Tool**  **(Aetiology, Base, Discharge, Edge, Floor, Size)**  **Baseline Score (mean +/-SD)**  Placebo: 9.32+/-1.44  Intervention: 9.08+/-1.61  **Week 8 (mean +/-SD)**  Placebo: 9.96+/-1.27  Intervention: 8.08+/-1.38  **Week 16 (mean +/-SD)**  Placebo: 10.72+/-1.24  Intervention: 7.0+/-1.26 | | Week 8: Significant ↓ favouring intervention (p=0.0000, 95%CI 1.13, 2.63)*  Week 16: Significant ↓ favouring intervention (p=0.0000, 95%CI 3.01, 4.43)* | |  |
| Yarahmadi 2021,  Iran, Vitamin E and C | **Control:** 2 completely closed (16.6%)  **Intervention**: 6 completely closed (46.1%) | Significant ↑ favouring intervention (P< 0.02) | NR | NR | NR | NR | **Wound area (cm^2^)**  **Control**  Baseline: 11.9+/-2.1  Week 8: 6.9+/-10  Change: -5.6+/-5.4  I**ntervention**  Baseline: 11.5+/-7.5 Week 8: 2.3+/-4.3 Change: -9.7+/-6.9 | Significant ↓ favouring intervention (p=0.019) | NR | NR | **Non-adjusted mean +/-n SD**  **Control**  Baseline: 2.7+/-1.4  12 weeks: 1.8+/-1.2  Change: -0.9+/-0.7  **Intervention**  Baseline: 2.7+/-1.0  12 weeks: 0.6+/-1.0  Change: -2.0+/-1.1 | Significant ↓ favouring intervention (p=0.010) | **Non-adjusted mean +/-n SD**  **Control**  Baseline: 4.3+/-3.1  12 weeks: 2.8+/-2.7  Change: -1.5+/-1.3  **Intervention**  Baseline: 5.0+/-2.3  12 weeks: 1.0+/-1.7  Change: -4.0+/-2.9 | Significant ↓ favouring intervention (p=0.023) | NR | | NR | |  |
| Das 2022,  India, Amino acids | NR | NR | NR | NR | NR | NR | NR | NR | NR | NR | NR | NR | NR | NR | **Southampton Scoring System**  **At Day 15**  **Normal Healing, n(%):**  Control: 8(26.67)  Intervention: 20(66.67)  **Minor Complication, n(%):**  Control: 9 (30)  Intervention: 7 (23.33)  **Wound Infection, n(%):**  Control: 10(33.33)  Intervention: 3(10)  **Major Haematoma, n(%):**  Control: 3(10)  Intervention: 0(0)  **Asepsis Wound Scoring System**  **At Day 15**  **Satisfactory Healing, n(%):**  Control: 7(23.33)  Intervention: 20(66.67)  **Disturbance of Healing, n(%):**  Control: 10(33.33)  Intervention: 7(23.33)  **Minor Wound Infection, n(%):**  Control: 10(33.33)  Intervention: 3(10)  **Severe Wound Infection, n(%):**  Control: 3(10)  Intervention: 0(0) | | **Southampton Scoring System**  Significant ↓ favouring intervention (p=0.007)  Chi Square = 12.16  **Asepsis Wound Scoring System**  Significant ↓ favouring intervention (p=0.006)  Chi Square = 12.27 | |  |
| Armstrong 2014,  USA, Europe and Taiwan, Arginine, glutamine and HMB | Proportion of people with total wound closure within 16 weeks  Control  Intent to treat: 46.7%  Intervention  Intent to treat: 50.1% | NS difference (p=0.6246, 95%CI -0.17, 0.10)* | NR | NR | **>= 15% at 1 week, n/N (%)**  Control: 79/128 (61.7)  Intervention: 71/112 (63.4)  **>= 50% at 4 weeks, n/N (%)**  Control: 46/101 (45.5)  Intervention: 56/95 (58.9) | Wound reduced ≥15%: NS difference (p=0.789)  Wound reduced≥50%: NS difference (p=0.087) | **Wound area (cm^2^)**  **Baseline median (min, max):**  Control: 1.8(0.3, 9.9)  Intervention: 1.6(0.4, 17.5)  **Median (Q1,Q3), n**  **Week 1**  Control: 1.8(1.2, 3.5), 139  Intervention: 1.65(1.2, 3.1), 126  **Week 4**  Control: 0.8(0.4, 2.0), 112  Intervention: 0.7(0.4, 2.1), 102  **Week 8**  Control: 0.8(0.2, 2.2), 79  Intervention: 0.7(0.3, 1.85), 84  **Week 12**  Control: 0.75(0.2, 2.15), 64  Intervention: 0.9(0.2, 2.8), 67  **Week 16**  Control: 1.0 (0.4, 2.2), 65  Intervention: 1.1(0.3, 3.9), 61 | Baseline NS difference (p=0.621)  Week 1 NS difference (p=0.621)  Week 4 NS difference (p=0.961)  Week 8 NS difference (p=0.732)  Week 12 NS difference (p=0.685)  Week 16 NS difference (p=0.623) | NR | NR | NR | NR | NR | NR | NR | | NR | |  |
| Eneroth 2004,  Sweden, Fortimel | No. people healing at 6 months excluding those who withdrew  Control: 8/23 (35%)  Intervention: 7/17 (41%)  No. wounds healed at 6 months including those who withdrew  Control: 10/27 (37%)  Intervention: 12/26 (46%) | 6 months excluding those withdrawn: NS difference (p=0.6985, 95%CI -0.36, 0.24)*  6 months including those withdrawn: NS difference (p= 0.5061, 95%CI -0.35, 0.17)* | NR | NR | NR | NR | NR | NR | NR | NR | NR | NR | NR | NR | NR | | NR | |  |
| Yanes-Quesada  2021,  Cuba, Diamel | **Achieved total epithelialisation n(%)**  Control: 34 (68%)  Intervention: 43 (86%) | Significant ↑ favouring intervention (p=0.032) | NR | NR | NR | NR | NR | NR | NR | NR | NR | NR | NR | NR | NR | | NR | |  |
| **Nutrition education (n=3)** | | | | | | | | | | | | | | | | | | | |
| Basiri 2020,  USA, Dietitian and Boost Glucose Control supplement | People with complete wound closure at 12 weeks, n  Control = 10/14 (71%)  Intervention = 9/15 (60%) | NS difference (p=0.5340, 95%CI -0.23, 0.45)* | NR | NR | **Control**  Baseline mean wound area: 45+/-15mm2  Decrease in wound area:  Week 4: 3.6  Week 8: 2.7  Week 12: 2.9  **Intervention**  Baseline mean wound area: 45+/-11mm2  Decrease in wound area:  Week 4: 11.8  Week 8: 3.6  Week 12: 3.4 | Significant ↓ in wound size favouring intervention during the first 4 weeks (p=0.01) | NR | NR | NR | NR | NR | NR | NR | NR | NR | | NR | |  |
| Sung 2021,  Australia, MDT | NR | NR | **Control**  Number of wounds per patient:  June: 1.36+/-0.70  July: 1.24  August: 1.17  September: 0.97  October: 0.97  November: 0.85  37.3% reduction in total numbers of wounds per patient  **Intervention**  Number of wounds per patient:  June: 1.58+/-0.90  July: 1.62  August: 1.37  September: 1.18  October: 1.14  November: 1.06  33.4% reduction in total numbers of wounds per patient | Reduction in the total number of DFUs: NS (p=0.971)  Reduction in wound size: NS (p=0.526) | **Wound size (cm^3^)**  **Control**  Baseline Mean+/-SD: 1.54 ± 6.73  From figure:  June 16 Mean: 2.12  November 16 Mean: 1.00  52.4% mean reduction in wound size  **Intervention**  Baseline Mean+/-SD: 3.9 ± 13.1  From figure:  June 16 mean: 3.84  November 16 mean: 1.56  60.1% mean reduction in wound size | NS difference (p=0.526) | NR | NR | NR | NR | NR | NR | NR | NR | NR | | NR | |  |
| Yang 2023,  China, Early nurse-led nutrition intervention | **Control, n(%)**  Cure: 43 (43)  Get better: 44 (44)  Invalid: 13 (13)  **Intervention, n(%)**  Cure: 58 (58)  Get better: 37 (37)  Invalid: 5 (5) | Healing: Significant ↑ favouring intervention (p<0.05) | NR | NR | NR | NR | NR | NR | NR | NR | NR | NR | NR | NR | NR | | NR | |  |
| Abbreviations  MDT = multidisciplinary team  HMB = beta-hydroxy-beta-methylbutyrate  Mg = magnesium  NS = Non-significant  NR = Not Reported  Q1 = Quartile 1  Q3 = Quartile 3  IQR = Interquartile Range  w/ = with  w/o = without  *= between group differences calculated from individual group summary statistics   1. Values are adjusted for baseline values of each biochemical variable, age and baseline BMI. 2. Values are adjusted for baseline values of each biochemical variable.   *Note:* results non-adjusted unless specified | | | | | | | | | | | | | | | | | | | |
