# Supplementary material for: Effectiveness of dietary interventions in individuals with diabetes for preventing and healing chronic wounds; a systematic review with meta‐analysis
Source: Diabet Med. 2025 Jul 9;42(9):e70100. doi: 10.1111/dme.70100 (PMC12352720; doi:10.1111/dme.70100)
Supplement: Supplementary file 1 — Data S1. [file DME-42-e70100-s001.zip › dme70100-sup-0009-TableS4..docx]

| **Supplementary Table 4. Secondary outcome other nutrition measures and other secondary outcome measures including between-group difference reported in studies investigating the effectiveness of nutrition interventions for individuals with diabetes-related foot ulceration.** | | | | | | | | | | |
| --- | --- | --- | --- | --- | --- | --- | --- | --- | --- | --- |
| **Reference, country** | **Dietary intake** | **Dietary intake**  **between group difference at follow-up between group difference at follow-up** | **Eating behaviours** | **Eating behaviours between group difference at follow-up** | **Nutritional status** | **Nutritional status**  **between group difference at follow-up** | **Patient satisfaction** | **Patient satisfaction between group difference at follow-up** | **Quality of life** | **Quality of life between group difference at follow-up** |
| **Single nutrient supplement studies (Reported outcomes n=5)** | | | | | | | | | | |
| Bashmakov 2014,  Egypt, Trans-resveratrol | NR | NR | NR | NR | NR | NR | NR | NR | NR | NR |
| Gunton 2021,  Australia, Vitamin C | NR | NR | NR | NR | NR | NR | NR | NR | NR | NR |
| Halschou-Jensen 2021,  Denmark, Vitamin D | NR | NR | NR | NR | NR | NR | NR | NR | NR | NR |
| Kamble 2020,  India, Vitamin D | NR | NR | NR | NR | NR | NR | NR | NR | NR | NR |
| Mozaffari-Khosravi 2016,  Iran, Vitamin D | NR | NR | NR | NR | NR | NR | NR | NR | NR | NR |
| Rangabashyam 2020,  India, Vitamin D | NR | NR | NR | NR | NR | NR | NR | NR | NR | NR |
| Razzaghi 2017,  Iran, Vitamin D | **Mean Energy (kcal/day) throughout trial**  Control: 2165+/-210  Intervention: 2238+/-217  **Mean CHO (g/d)**  Control: 299.6+/-43.0  Intervention: 310.3+/-50.0  **Total Dietary Fibre (g/d)**  Control: 19.7+/-4.3  Intervention: 18.3+/-4.3  **Mean Protein (g/d)**  Control: 80.3+/-19.1  Intervention: 80.9+/-11.2  **Mean Fat (g/d)**  Control: 75.7+/-13.8  Intervention: 78.3+/-14.0  **Short-chain Fatty Acids (g/d)**  Control: 23.5+/-4.9  Intervention: 24.6+/-4.6  **Polyunsaturated Fatty Acids (g/d)**  Control: 23.3+/-5.5  Intervention: 24.3+/-6.3  **Monounsaturated Fatty Acids (g/d)**  Control: 20.7+/-6.0  Intervention: 21.3+/-5.6  **Cholesterol (mg/d)**  Control: 188.0+/-106.3  Intervention: 198.0+/-106.3  **Vitamin D (µg/d)**  Control: 2.9+/-0.8  Intervention: 3.0+/-0.9  **Calcium (mg/d)**  Control: 1073.4+/-146.9  Intervention: 1116.6+/-185.7 | **Energy**  NS difference (p=0.19)  **CHO**  NS difference (p=0.37)  **Protein**  NS difference (p=0.89)  **Fat**  NS difference (p=0.46)  **SFAs**  NS difference (p=0.35)  **PUFAs**  NS difference (p=0.54)  **MUFAs**  NS difference (p=0.70)  **Cholesterol**  NS difference (p=0.71)  **TDF**  NS difference (p=0.22)  **Vitamin** **D**  NS difference (p=0.69)  **Calcium**  NS difference (p=0.32) | NR | NR | NR | NR | NR | NR | NR | NR |
| Jain 2012,  India, Vitamin E | NR | NR | NR | NR | NR | NR | NR | NR | NR | NR |
| Mohseni 2018,  Iran, Probiotic | Based on the 3-day dietary records, we found no significant difference in mean dietary macronutrient and micronutrient intakes between the 2 groups (data not shown). | Reports NS difference however data NR | NR | NR | NR | NR | NR | NR | NR | NR |
| Mokhtari 2020,  Iran, Nanocurcumin | NR | NR | NR | NR | NR | NR | NR | NR | NR | NR |
| Momen-Heravi 2017,  Iran, Zinc | **Mean Energy (kcal/day) throughout trial**  Control: 2219+/-212  Intervention: 2183+/-220  **Mean CHO (g/d)**  Control: 308.1+/-46.1  Intervention: 300.8+/-47.6  **Total Dietary Fibre (g/d)**  Control: 19.1+/-4.4  Intervention: 19.0+/-4.3  **Mean Protein (g/d)**  Control: 81.1+/-15.6  Intervention: 80.0+/-15.6  **Mean Fat (g/d)**  Control: 77.3+/-14.4  Intervention: 76.7+/-13.6  **Short-chain Fatty Acids (g/d)**  Control: 24.4+/-5.1  Intervention: 23.7+/-4.5  **Polyunsaturated Fatty Acids (g/d)**  Control: 23.6+/-5.7  Intervention: 24.0+/-6.2  **Monounsaturated Fatty Acids (g/d)**  Control: 21.2+/-6.0  Intervention: 20.8+/-5.7  **Cholesterol (mg/d)**  Control: 201.5+/-111.8  Intervention: 185.0+/-100.1  **Zinc (mg/d)**  Control: 9.9+/-2.7  Intervention: 9.7+/-2.4 | **Energy**  NS difference (p=0.52)  **CHO**  NS difference (p=0.60)  **TDF**  NS difference (p=0.96)  **Protein**  NS difference (p=0.77)  **Fat**  NS difference (p=0.88)  **SFAs**  NS difference (p=0.61)  **PUFAs**  NS difference (p=0.80)  **MUFAs**  NS difference (p=0.77)  **Cholesterol**  NS difference (p=0.53)  **Zinc**  NS difference (p=0.67) | NR | NR | NR | NR | NR | NR | NR | NR |
| Razzaghi 2018,  Iran, Magnesium | Based on the 3-day dietary records obtained at base-  line, end of treatment, and throughout the trial, no significant differences were observed between the two groups in terms of dietary intakes of energy, carbohydrates, proteins, fats, saturated fatty acids, polyunsaturated fatty acids, monounsaturated fatty acids, cholesterol, crude fiber, total dietary fiber, magnesium, and manganese (data not shown). | NR | NR | NR | NR | NR | NR | NR | NR | NR |
| Soleimani 2017,  Iran, Omega-3 | **Throughout the trial**  **Mean Energy (kcal/day) throughout trial**  Control: 2344+/-139  Intervention: 2326+/-190  **Mean CHO (g/d)**  Control: 320.3+/-33.6  Intervention: 318.2+/-46.7  **Total Dietary Fibre (g/d)**  Control: 17.8+/-3.2  Intervention: 18.5+/-4.7  **Mean Protein (g/d)**  Control: 87.1+/-14.4  Intervention: 85.5+/-23.9  **Mean Fat (g/d)**  Control: 82.8+/-14.5  Intervention: 82.6+/-19.5  **Short-chain Fatty Acids (g/d)**  Control: 24.6+/-6.4  Intervention: 24.7+/-7.6  **Polyunsaturated Fatty Acids (g/d)**  Control: 26.7+/-5.6  Intervention: 26.1+/-5.5  **Monounsaturated Fatty Acids (g/d)**  Control: 21.9+/-5.5  Intervention: 22.8+/-8.0  **Cholesterol (mg/d)**  Control: 224.8+/-124.9  Intervention: 240.2+/-165.1  **Omega-3 (g/d)**  Control: 1.0+/-0.4  Intervention: 1.0+/-0.3  **Omega-6 (g/d)**  Control: 23.5+/-3.6  Intervention: 24.1+/-4.2 | **Energy**  NS difference (p=0.66)  **CHO**  NS difference (p=0.84)  **TDF**  NS difference (p=0.53)  **Protein**  NS difference (p=0.77)  **Fat**  NS difference (p=0.96)  **SFAs**  NS difference (p=0.97)  **PUFAs**  NS difference (p=0.64)  **MUFAs**  NS difference (p=0.62)  **Cholesterol**  NS difference (p=0.68)  **Omega-3**  NS difference (p=0.45)  **Omega-6**  NS difference (p=0.52) | NR | NR | NR | NR | NR | NR | NR | NR |
| **Multi-nutrient supplement studies (Reported outcomes n=1** | | | | | | | | | | |
| Afzali 2019,  Iran, Mg and vitamin E | We found no significant difference in mean dietary macro- and  micro-nutrient intakes between the two groups (Data not shown). | NR | NR | NR | NR | NR | NR | NR | NR | NR |
| Bosede 2012,  Nigeria, Vitamin E, C and selenium | NR | NR | NR | NR | NR | NR | NR | NR | NR | NR |
| Yarahmadi 2021,  Iran, Vitamin E and C | NR | NR | NR | NR | NR | NR | NR | NR | NR | NR |
| Das 2022,  India, Amino acids | NR | NR | NR | NR | NR | NR | NR | NR | NR | NR |
| Armstrong 2014,  USA, Europe and Taiwan, Arginine, glutamine and HMB | NR | NR | NR | NR | NR | NR | NR | NR | NR | NR |
| Eneroth 2004,  Sweden, Fortimel | NR | NR | NR | NR | NR | NR | NR | NR | NR | NR |
| Yanes-Quesada  2021,  Cuba, Diamel | NR | NR | NR | NR | NR | NR | NR | NR | NR | NR |
| **Nutrition education (Reported outcomes n=2)** | | | | | | | | | | |
| Basiri 2020,  USA, Dietitian and Boost Glucose Control supplement | **Mean energy compared to National Pressure Ulcer Advisory Panel (NPUAP) recommendation. Mean% +/- SD**  Control  Baseline: 42.7% +/- 16.8  Intervention  Baseline: 53.2% +/-22.6  **Protein: Mean compared to National Pressure Ulcer Advisory Panel (NPUAP) recommendation. Mean% +/- SD**  Control  Baseline: 43.1% +/- 18.2  Intervention  Baseline: 54.5% +/- 24.7  **Micronutrients**  **Vitamin A (ug RAE)**  Control  Baseline: 168.0  Week 4: 155.8  Week 8: 109.5  Week 12: 120.5  Intervention  Baseline: 126.6  Week 4: 298.2  Week 8: 286.0  Week 12: 290.9  **Vitamin C (mg)**  Control  Baseline: 36.0  Week 4: 33.0  Week 8: 36.0  Week 12: 51.3  Intervention  Baseline: 47.0  Week 4: 141.1  Week 8: 144.2  Week 12: 144.2  **Vitamin E (mg)**  Control  Baseline: 1.3  Week 4: 1.0  Week 8: 1.0  Week 12: 0.8  Intervention  Baseline: 0.7  Week 4: 22.7  Week 8: 23.2  Week 12: 23.2  **Zinc (mg)**  Control  Baseline: 1.7  Week 4: 2.6  Week 8: 2.8  Week 12: 3.0  Intervention  Baseline: 2.2  Week 4: 4.5  Week 8: 5.0  Week 12: 5.5  **Copper (ug)**  Control  Baseline: 0.14  Week 4: 0.23  Week 8: 0.19  Week 12: 0.22  Intervention  Baseline: 0.15  Week 4: 0.55  Week 8: 0.53  Week 12: 0.56  For all participants Mean intake compared to DRI:  Baseline intake  Vitamin E 11.62%  Vitamin C: 100.00%  Vitamin A (REA): 34.30%  Copper: 25.60%  Maganese: 36.00%  Zinc: 26.90% | **Protein intake**  NS difference (P-value NR)  **Copper**  Significant difference however direction of significance NR (p < 0.001)  **Vitamin A**  Significant difference however direction of significance NR (p = 0.001)  **Vitamin C**  Significant difference however direction of significance NR (p < 0.001)  **Vitamin E**  Significant difference however direction of significance NR (p < 0.001) | **Control**  Baseline  Dietary restrictions (Y/N): 1/13  Appetite problem (Y/N): 1/13  Visited RD(Y/N): 3/11  Need food (Y/N): 1/13  **Intervention**  Baseline  Dietary restrictions (Y/N): 2/13  Appetite problem (Y/N): 1/14  Visited RD(Y/N): 7/8  Need food (Y/N): 2/13 | **Dietary restrictions**  NS difference (p=1)  **Appetite problem**  NS difference (p=1)  **Visited RD**  NS difference (p=0.3)  **Need food**  NS difference (p=0.6) | NR | NR | NR | NR | NR | NR |
| Sung 2021,  Australia, MDT | NR | NR | NR | NR | NR | NR | NR | NR | NR | NR |
| Yang 2023,  China, Early nurse-led nutrition intervention | NR | NR | NR | NR | **NRS2002 score**  **Control**  Baseline: 2.16+/-1.82  After: 1.59+/-1.74  **Intervention**  Baseline: 1.90+/-1.63  After: 0.66+/-1.49 | **Baseline**  NS difference (p=0.564)  **After**  Significant ↓ favouring intervention (p=0.024) | **Control**  Satisfied: 52 (52)  Generally: 33 (33)  Dissatisfied: 15 (15)  **Intervention**  Satisfied: 78 (78)  Generally: 18 (18)  Dissatisfied: 4 (4) | **Satisfied:** Significant ↑ favouring intervention (p=0.0001, 95%CI -0.39, -0.13)*  **Generally:** Significant ↑ favouring control (p=0.0150, 95%CI 0.03, 0.27)*  **Dissatisfied** Significant ↓ favouring intervention (p=0.0080, 95%CI 0.03, 0.19)* | **Control**  Active coping score  Before: 21.51+/-2.72  After: 25.58+/-3.17  Negative coping score  Before: 13.01+/-2.42  After: 7.18+/-1.26  **Intervention**  Active coping score  Before: 21.57+/-2.74  After: 29.66+/-3.41  Negative coping score  Before: 13.08+/-2.47  After: 4.99+/-1.01 | Active coping score  Before: NS difference (p=0.914)  After: Significant ↑ favouring intervention (p=0.001)  Negative coping score  Before: NS difference (p=0.921)  After: Significant ↓ favouring intervention (p=0.001) |
| Abbreviations  NS = Non-significant  NR = Not Reported  RD = Registered Dietitian  TG = Triglycerides  VLDL = Very Low-Density Lipoprotein  TC = Total Cholesterol  LDL = Low-Density Lipoprotein  HDL = High-Density Lipoprotein  SD = Standard Deviation  CI = Confidence Intervals   1. Values are adjusted for baseline values of each biochemical variable, age and baseline BMI. 2. Values are adjusted for baseline values of each biochemical variable.   ^The worst result was selected in order to not misrepresent the data, as different tables reported different results. Nil email found for authors on the published paper.  *= between group differences calculated from individual group summary statistics  *Note:* results non-adjusted unless specified  *Note:* A calculated conversion completed for studies that report HbA1c in mg/dl as per journal author guidelines | | | | | | | | | | |
