# Supplementary material for: Effectiveness of dietary interventions in individuals with diabetes for preventing and healing chronic wounds; a systematic review with meta‐analysis
Source: Diabet Med. 2025 Jul 9;42(9):e70100. doi: 10.1111/dme.70100 (PMC12352720; doi:10.1111/dme.70100)
Supplement: Supplementary file 1 — Data S1. [file DME-42-e70100-s001.zip › dme70100-sup-0010-TableS5.docx]

| **Supplementary Table 5. Secondary outcome glycaemic control markers and nutrient biochemistry measures including between-group difference reported in studies investigating the effectiveness of nutrition interventions for individuals with diabetes-related foot ulceration.** | | | | | | | | | |
| --- | --- | --- | --- | --- | --- | --- | --- | --- | --- |
| **Reference, country** | **Fasting Plasma Glucose (mg/dl)** | **Fasting Plasma Glucose between group difference at follow-up** | **Random Blood Sugar** | **Random Blood Sugar**  **between group difference at follow-up** | **HbA1c (%)** | **Calculated Conversion for HbA1c^a^**  **(mmol/mol +/- SD mmol/mol (%))** | **HbA1c between group difference at follow-up** | **Biochemistry Nutrients** | **Nutrients between group difference at follow-up** |
| **Single nutrient supplement studies (Reported outcome n=13)** | | | | | | | | | |
| Bashmakov 2014,  Egypt, Trans-resveratrol | Control:  Baseline:161.2(72.8)  Mean within group changes (95%CI): -19.70 (mean reduction average) (−27.17, 66.57) (SD 75.62*)  Intervention:  Baseline: 201.8(81.8)  Mean within group changes (95%CI): -31.57 (mean reduction average) (3.45, 59.69) (SD 53.68*) | NS difference (p=0.6565, 95%CI -42.72, 66.46)* | NR | NR | NR | NR | NR | NR | NR |
| Gunton 2021,  Australia, Vitamin C | NR | NR | NR | NR | **At inclusion**  **HbA1c(%)**  Control: Median(range): 9.5(5.9-11.4)  Intervention: Median(range): 9.5(9.5-9.6) | **At inclusion**  **HbA1c**  Median  Control: Median: 80 (9.5)  Intervention: Median: 80 (9.5) | NR | Vitamin C(umol/l): Mean was 30.5umol/l overall with IQR (4-52). Eight of  the sixteen subjects were vitamin C deficient, with four subjects  having undetectable levels. Four people in each treatment group had baseline deficiency. | NR |
| Halschou-Jensen 2021,  Denmark, Vitamin D | NR | NR | NR | NR | Control  HbA1c at baseline: 9.8+/-2.1  Mean(95%CI)  Baseline: 9.8(8.9-10.7) (SD 2.15*)  4 weeks: 9.8(8.8-10.9) (SD 2.51*)  12 weeks: 9.9(8.7-11.1) (SD 2.87*)  24 weeks: 9.8(8.3-11.3) (SD 3.59*)  36 weeks: 10.1(7.9-12.4) (SD 5.38*)  48 weeks: 10.6(5.8-12.4) (SD 7.90*)  Intervention  HbA1c at baseline: 11.6+/-2.5  Mean(95%CI)  Baseline: 11.8(10.7-12.9) (SD 2.69*)  4 weeks: 11.3(10.1-12.5) (SD 2.94*)  12 weeks: 11.2(9.8-12.6) (SD 3.43*)  24 weeks: 10.8(8.9-12.6) (SD 4.53*)  36 weeks: 10.0(7.6-12.4) (SD 5.87*)  48 weeks: 11.2(7.4-15.0) (SD 9.30*) | Baseline: 84 +/-23.5 (9.8)  4 weeks: 84+/-27.4 (9.8)  12 weeks: 85+/-31.4 (9.9)  24 weeks: 84+/-39.2 (9.8)  36 weeks: 87+/-58.8 (10.1)  48 weeks: 92+/-86.3 (10.6)  Intervention  HbA1c at baseline: 11.6+/-2.5  Mean(95%CI)  Baseline: 105+/-29.4 (11.8)  4 weeks: 100+/-32.1 (11.3)  12 weeks: 99+/-37.5 (11.2)  24 weeks: 95+/-49.5 (10.8)  36 weeks: 86+/-64.2 (10.0)  48 weeks: 99+/-101.6 (11.2) | **P value at baseline:**  Significant difference (p=0.006)  **Week 4:** NS difference (p=0.0732, 95%CI -3.15, 0.15)*  **Week 12:** NS difference (p=0.1761, 95%CI -3.21, 0.61)*  **Week 24:** NS difference (p=0.4177, 95%CI -3.46, 1.46)*  **Week 36:** NS difference (p=0.9528, 95%CI -3.29, 3.49)*  **Week 48:** NS difference | **Vitamin D (Deficiency <50nmol/L).**  Control  Deficiency at baseline: n=11  Mean(95%CI)  Baseline: 55.1(43.3-67.2)  4 weeks: 62.6(52.1-72.9)  12 weeks: 67.8(56.3-70.2)  24 weeks: 68.1(55.1-80.7)  36 weeks: 73.5(57.8-88.6)  48 weeks: 79.8(55.7-89.8)  Intervention  Deficiency at baseline: n=12  Mean(95%CI)  Baseline: 52.4(42.4-62.6)  4 weeks: 82.3(72.3-92.8)  12 weeks: 91.6(82.0-101.3)  24 weeks: 110.3(94.3-125.7)  36 weeks: 92.2(76.5-107.6)  48 weeks: 88.3(59.6-116.7)  **Calcium (mmol/l)**  Control  Mean(95%CI)  Baseline: 1.27(1.24-1.29)  4 weeks: 1.27(1.25-1.29)  12 weeks: 1.27(1.25-1.29)  24 weeks: 1.25(1.23-1.28)  36 weeks: 1.25(1.20-1.29)  48 weeks: 1.24(1.17-1.30)  Intervention  Mean(95%CI)  Baseline: 1.27(1.24-1.29)  4 weeks: 1.26(1.23-1.28)  12 weeks: 1.28(1.25-1.31)  24 weeks: 1.29(1.26-1.31)  36 weeks: 1.27(1.23-1.32)  48 weeks: 1.27(1.19-1.35)  **Parathyroid hormone (pmol/L)**  Control  Mean(95%CI)  Baseline: 6.4(4.7-8.1)  4 weeks: 6.4(4.3-8.5)  12 weeks: 6.3(3.6-8.9)  24 weeks: 6.3(2.7-10.0)  36 weeks: 7.4(1.5-13.3)  48 weeks: 6.7(2.1-14.2)  Intervention  Mean(95%CI)  Baseline: 7.1(3.9-10.3)  4 weeks: 7.3(1.7-13.0)  12 weeks: 6.6(2.2-11.0)  24 weeks: 4.1(2.1-5.9)  36 weeks: 5.8(3.2-8.4)  48 weeks: 6.0(3.3-8.6) | **Vitamin D**  **4 weeks:** significant ↑ favouring intervention (p=0.0114, 95%CI -34.73, -4.67)*  **12 weeks:** significant ↑ favouring intervention (p=0.0003, 95%CI -36.13, -11.47)*  **24 weeks:** significant ↑ favouring intervention (p=0.0002, 95%CI -63.15, -21.25)*  **36 weeks:** NS difference (p=0.1015, 95%CI -41.23, 3.83)*  **48 weeks:** NS difference (p=0.6228, 95%CI -43.10, 26.10)*  **Calcium**  **4 weeks:** NS difference (p=0.5478, 95%CI -0.02, 0.04)*  **12 weeks:** NS difference (p=0.5857, 95%CI -0.05, 0.03)*  **24 weeks:** significant ↑ favouring intervention (p=0.0306, 95%CI -0.08, -0.00)*  **36 weeks:** NS difference (p=0.5453, 95%CI -0.09, 0.05)*  **48 weeks:** NS difference (p=0.5824, 95%CI -0.14, 0.08)*  **Parathyroid hormone**  **4 weeks:** NS difference (p=0.7750, 95%CI -7.21, 5.41)*  **12 weeks:** NS difference (p=0.9104, 95%CI -5.64, 5.04)*  **24 weeks:** NS difference (p=0.2943, 95%CI -1.98, 6.38)*  **36 weeks:** NS difference (p=0.6240, 95%CI -4.93, 8.13)*  **48 weeks:** NS difference (p=0.8340, 95%CI -5.99, 7.39)* |
| Kamble 2020,  India, Vitamin D | NR | NR | NR | NR | **HbA1c(%)**  Control:  Baseline: 9.8+/-3.5  End of trial: 9.9+/-4.8  Change: -0.1+/-0.8  Intervention:  Baseline: 9.1+/-2.1  End of trial: 7.9+/-2.7  Change: 1.2+/-0.03 | Control:  Baseline: 84+/-38(9.8)  End of trial: 85+/-53(9.9)  Intervention:  Baseline: 76+/-23(9.1)  End of trial: 63 +/-30(7.9) | Significant ↓ favouring intervention (p=0.0001) | **Vitamin D (Serum 25-hydroxyvitamin D (25(OH) D) (ng/ml)**  Control  Baseline: 20.5+/-9.3  End of trial: 20.1+/-8.5  Change: 0.4+/-0.3  Intervention  Baseline: 18.5+/-11.6  End of trial: 31+/-9.8  Change: -12.5+/-10.1 | Significant ↑ favouring intervention (p=0.0001) |
| Mozaffari-Khosravi 2016,  Iran, Vitamin D | Control  Baseline: 166.09+/-28.44  After 4 weeks: 154.17+/-28.53  Change: -11.9+/-6.9  Intervention  Baseline: 158.5+/-32.34  After 4 weeks: 137.21+/-25.68  Change: -21.3+/-11.1 | Baseline  NS difference (p=0.95)  After 4 weeks  Significant ↓ favouring intervention (p=0.03)  Change  Significant ↓ favouring intervention (p=0.001) | NR | NR | NR | NR | NR | **Vitamin D status (ng/ml) (25-hydroxyvitamin D)**  **Deficient(<20)/Sufficient(>20) at baseline**  Control  Deficient: 11(47.8)  Sufficient: 12(52.2)  Baseline: 27.91+/-19.7  After 4 weeks: 40.53+/-15.49  Change:12.6+/-5.0  Intervention  Deficient: 13(54.2)  Sufficient: 11(45.8)  Baseline: 23.03+/-15.98  After 4 weeks: 41.62+/-13.93  Change: 18.4+/-6.4 | Baseline  NS difference (p=0.56)  After 4 weeks  NS difference (p=0.80)  Change  Significant ↑ favouring intervention (p=0.001) |
| Rangabashyam 2020,  India, Vitamin D | **Fasting Blood Glucose (mg/dl)**  Control:  Week 1: 221.52+/-34.64  Week 6: 180.42+/-28.73  Decreased by 18.5%.  Intervention:  Week 1: 169.78+/-19.79  Week 6: 114.45+/-17.28  Decreased by 32.5% | Significant ↓ favouring intervention (p=0.0000, 95%CI 56.56, 75.38)* | **Postprandial Blood Glucose (mg/dl)**  Control:  Week 1: 240.96+/-33.16  Week 6: 195.52+/-27.71  Intervention:  Week 1: 196.10+/-16.58  Week 6: 132.40+/-13.48  Reduced significantly by 32.4%. | **Postprandial Blood Glucose**  Significant ↓ favouring intervention (p=0.0000, 95%CI 54.47, 71.77)* | **HbA1c (%)**  Control:  Week 1: 9.54+/-0.91  Week 6: 8.12+/-0.62  Decrease of 14.8%  Intervention:  Week 1: 7.59+/-0.65  Week 6: 6.04+/-0.48  Significant decrease of 20.4% | Control:  Week 1: 80+/-10(9.5)  Week 6: 65+/-7(8.1)  Intervention:  Week 1: 60+/-7(7.6)  Week 6: 42+/-5(6.0) | Significant ↓ favouring intervention (p=0.0000, 95%CI 1.86, 2.30)* | **Vitamin D (ng/ml)**  Control  Week 1: 15.24+/-1.67  Week 6: 15.83+/-1.43  Intervention  Week 1: 17.09+/-3.94  Week 6: 40.49+/-3.58  (Increased by 136.9%)  **Serum Calcium (mg/dl)**  Control  Week 1: 6.45+/-0.53  Week 6: 7.50+/-0.52  Intervention  Week 1: 6.78+/-0.72  Week 6: 9.34+/-0.29  (Increased by 37.7%) | **Vitamin D**  Significant ↑ favouring intervention (p=0.0000, 95%CI -25.74, -23.58)*  **Calcium**  Significant ↑ favouring intervention (p=0.0000, 95%CI -2.01, -1.67)* |
| Razzaghi 2017,  Iran, Vitamin D | **Adjusted^a^**  Control: -9.3+/-6.9  Intervention: -35.5+/-6.9 | Significant ↓ favouring intervention (p=0.01) | NR | NR | **Adjusted^a^**  Control: -0.2+/-0.1  Intervention: -0.5+/-0.1 | NA as HbA1c reported as mean change | Significant ↓ favouring intervention (p=0.04) | **Adjusted^a^**  **Vitamin D (ng/ml)**  Control: -0.005+/-1.8  Intervention: 11.1+/-1.8 | Significant ↑ favouring intervention (p<0.001) |
| Jain 2012,  India, Vitamin E | Primary prevention (w/o complications)  Type 1 diabetes (w/o complications) control group  Baseline: 101.4+/-13.35  12 months: 103+/-13.21  18 months: 105.4+/-10.15  24 months: 107+/-11.12  Type 1 diabetes (w/o complications) intervention group  Baseline: 102.8+/-14.92  12 months: 104+/-13.6  18 months: 104.5+/-12.15  24 months: 105+/-12.32  Type 2 diabetes w/o complications) control group  Baseline: 106.3+/-13.3  12 months: 107+/-13.85  18 months: 107.8+/-15.61  24 months: 108.2+/-14.05  Type 2 diabetes (w/o complications) intervention group  Baseline: 106.1+/-16.51  12 months: 105+/-15.12  18 months: 103.8+/-15.92  24 months: 102+/-16  Secondary prevention  Type 1 diabetes w/ complications control group  Baseline: 106.7+/-22.49  12 months: 102.6+/-11.54  18 months: 107.1+/-13.2  24 months: 112.1+/-14.2  Type 1 diabetes w/ complications intervention group  Baseline: 107.5+/-14.30  12 months: 103+/-14.6  18 months: 105+/-11.91  24 months: 109.3+/-12.85  Type 2 diabetes w/ complications control group  Baseline: 111.2+/-7.78  12 months: 110+/-5.91  18 months: 110+/-5.91  24 months: 108.5+/-5.5  Type 2 diabetes w/ complications intervention group  Baseline: 111.6+/-13.58  12 months: 107.5+/-12.95  18 months: 107+/-12.8  24 months: 106+/-17.96 | Primary prevention (w/o complications)  **Type 1 diabetes (w/o complications)**  **12 months:** NS difference (p=0.7664, 95%CI -7.70, 5.70)*  **18 months:** NS difference (p=0.7489, 95%CI -4.69, 6.49)*  **24 months:** NS difference (p=0.4980, 95%CI -3.86, 7.86)*  **Type 2 diabetes (w/o complications)**  **12 months:** NS difference (p=0.5831, 95%CI -5.25, 9.25)*  **18 months:** NS difference (p=0.3141, 95%CI -3.88, 11.88)*  **24 months:** NS difference (p=0.1046, 95%CI -1.32, 13.72)*  Secondary prevention  **Type 1 diabetes w/ complications**  **12 months:** NS difference (p=0.8857, 95%CI -5.91, 5.11)*  **18 months:** NS difference (p=0.4303, 95%CI -3.17, 7.37)*  **24 months:** NS difference (p=0.3294, 95%CI -2.87, 8.47)*  **Type 2 diabetes w/ complications**  **12 months:** NS difference (p=0.2419, 95%CI -1.72, 6.72)*  **18 months:** NS difference (p=0.1570, 95%CI -1.18, 7.18)*  **24 months:** NS difference (p=0.3744, 95%CI -3.06, 8.06)* | **Postprandial Blood Glucose (%)**  Primary prevention (w/o complications)  Type 1 diabetes (w/o complications) control group  Baseline: 180.2+/-8.69  12 months: 182.7+/-8.25  18 months: 184.5+/-27.22  24 months: 186+/-16.45  Type 1 diabetes (w/o complications) intervention group  Baseline: 180+/-7.99  12 months: 181+/-12.84  18 months: 179+/-24.50  24 months: 178+/-14.73  Type 2 diabetes w/o complications) control group  Baseline: 181.7+/-6.76  12 months: 182.8+/-7  18 months: 183.5+/-8.29  24 months: 183+/-8.49  Type 2 diabetes (w/o complications) intervention group  Baseline: 181+/-8.83  12 months: 181+/-9.45  18 months: 185+/-7.55  24 months: 178.2+/-7.58  Secondary prevention  Type 1 diabetes w/ complications control group  Baseline: 183+/-8.34  12 months: 183.8+/-7.6  18 months: 184.2+/-8.14  24 months: 185.5+/-7.39  Type 1 diabetes w/ complications intervention group  Baseline: 183.9+/-8.31  12 months: 182.4+/-7.4  18 months: 182+/-6.9  24 months: 182+/-5.64  Type 2 diabetes w/ complications control group  Baseline: 178.7+/-6.7  12 months: 180+/-5.6  18 months: 183+/-7.02  24 months: 184.5+/-6.56  Type 2 diabetes w/ complications intervention group  Baseline: 180.1+/-8.26  12 months: 181.8+/8.87  18 months: 182+/8.30  24 months: 181.2+/8.14 | **Postprandial Blood Glucose**  Primary prevention (w/o complications)  **Type 1 diabetes (w/o complications)**  **12 months:** NS difference (p=0.5878, 95%CI -3.92, 6.86)*  **18 months:** NS difference (p=0.3988, 95%CI -7.44, 18.44)*  **24 months:** Significant ↓ favouring intervention (p=0.0447, 95%CI 0.20, 15.8)*  **Type 2 diabetes (w/o complications)**  **12 months:** NS difference (p=0.3899, 95%CI -2.36, 5.96)*  **18 months:** NS difference (p=0.4521, 95%CI -5.46, 2.46)*  **24 months:** Significant ↓ favouring intervention (p=0.0201, 95%CI 0.78, 8.82)*  Secondary prevention  **Type 1 diabetes w/ complications**  **12 months:** NS difference (p=0.3784, 95%CI -1.74, 4.54)*  **18 months:** NS difference (p=0.1702, 95%CI -0.96, 5.36)*  **24 months:** Significant ↓ favouring intervention (p=0.0133, 95%CI 0.75, 6.25)*  **Type 2 diabetes w/ complications**  **12 months:** NS difference (p=0.2528, 95%CI -4.91, 1.31)*  **18 months:** NS difference (p=0.5388, 95%CI -2.22, 4.22)*  **24 months:** Significant ↓ favouring intervention (p=0.0370, 95%CI 0.20, 6.40)* | NR | NR | NR | NR | NR |
| Mohseni 2018,  Iran, Probiotic | **Adjusted^a^**  Control: -8.7+/-6.2  Intervention: -26.8+/-6.2 | Significant ↓ favouring intervention (p=0.04) | NR | NR | **Adjusted^a^**  Control: -0.2+/-0.1  Intervention: -0.5+/-0.1 | NA as HbA1c reported as mean change | Significant ↓ favouring intervention (p=0.004) | NR | NR |
| Mokhtari 2020,  Iran, Nanocurcumin | **Adjusted^b^**  Control  Baseline: 190.2+/-68.9  Week 12: 148.0+/-45.0  Intervention  Baseline: 198.9+/-79.6  Week 12: 136.1+/-32.5 | Significant ↓ favouring intervention (p=0.02, B(95%CI): -15.54(-29.46, -1.62)) | NR | NR | **Adjusted^b^**  Control  Baseline: 8.4+/-1.7  Week 12: 8.1+/-1.7  Intervention  Baseline: 8.6+/-2.3  Week 12: 8.3+/-2.2 | **Adjusted^b^**  Control  Baseline: 68+/-19(8.4)  Week 12: 65+/-19(8.1)  Intervention  Baseline: 70+/-25(8.6)  Week 12: 67+/-24(8.3) | NS difference (p=0.44, B(95%CI): -0.07(-0.26, 0.11)) | NR | NR |
| Momen-Heravi 2017,  Iran, Zinc | **Adjusted^a^**  Control: -2.2+/-10.2  Intervention: -42.1+/-10.2 | Significant ↓ favouring intervention (p=0.008) | NR | NR | **Adjusted^a^**  Control: -0.04+/-0.1  Intervention: -0.5+/-0.1 | NA as HbA1c reported as mean change | Significant ↓ favouring intervention (p=0.01) | **Adjusted Change^a^**  **Zinc (mg/dl)**  Control: -3.5+/-0.8  Intervention: 12.7+/-0.8 | Significant ↑ favouring intervention (p<0.001) |
| Razzaghi 2018,  Iran, Magnesium | **Adjusted^a^**  Control: -18.5+/-9.8  Intervention: -37.5+/-9.8 | NS difference (p=0.18) | NR | NR | **Adjusted^a^**  Control: -0.2+/-0.1  Intervention: -0.6+/-0.1 | NA as HbA1c reported as mean change | NS difference (p=0.10) | **Adjusted^a^**  **Magnesium (mg/dL)**  Control: -0.1+/-0.03  Intervention: 0.3+/-0.03 | Significant ↑ favouring intervention (p=<0.001) |
| Soleimani 2017,  Iran, Omega-3 | **Adjusted^a^**  Control: -8.6+/-10.5  Intervention: -40.1+/-10.5 | Significant ↓ favouring intervention (p=0.03) | NR | NR | **Adjusted^a^**  Control: -0.02+/-0.2  Intervention: -1.0+/-0.2 | NA as HbA1c reported as mean change | Significant ↓ favouring intervention (p<0.001) | NR | NR |
| **Multi-nutrient supplement studies (Reported outcome n=6)** | | | | | | | | | |
| Afzali 2019,  Iran, Mg and vitamin E | **Adjusted^a^**  Control:  Baseline: 164.1+/-39.3  Week 12: 156.0+/-38.4  Intervention:  Baseline: 173.7+/-60.8  Week 12: 149.9+/-47.5 | Significant ↓ favouring intervention (p=0.001, B(95%CI): -13.41 (-20.96, -5.86)) | NR | NR | **Adjusted^a^**  Control:  Baseline: 7.6+/-0.6  Week 12: 7.4+/-0.5  Intervention:  Baseline: 7.4+/-0.8  Week 12: 6.8+/-0.6 | **Adjusted^a^**  Control:  Baseline: 60+/-7 (7.6)  Week 12: 57+/-6 (7.4)  Intervention:  Baseline: 57 +/-9 (7.4)  Week 12: 51 +/-7 (6.8) | Significant ↓ favouring intervention (p<0.001, B(95%CI): -0.32 (-0.48, -0.16)) | **Adjusted^a^**  **Magnesium (mg/dL)**  Control  Baseline: 1.51+/-0.15  Week 12: 1.50+/-0.15  Intervention  Baseline: 1.55+/-0.18  Week 12: 1.83+/-0.20 | Significant ↑ favouring intervention  (p<0.001, B(95%CI): 0.29 (0.24, 0.34)) |
| Bosede 2012,  Nigeria, Vitamin E, C and selenium | Control  Baseline: 13.10+/-3.50  Week 8: 12.50+/-3.52  Week16: 11.78+/-3.63  Intervention  Baseline: 12.92+/-3.57  Week 8: 11.98+/-3.36  Week16: 11.38+/-3.40 | **Week 8:** NS difference (p=0.5956, 95%CI -1.44, 2.48)*  **Week 16:** NS difference (p=0.6894, 95%CI -1.60, 2.40)* | NR | NR | Control  Baseline: 8.59+/-2.10  Week 8: 8.18+/-1.90  Week16: 7.98+/-1.86  Intervention  Baseline: 8.43+/-1.94  Week 8: 7.92+/-1.77  Week16: 7.55+/-1.67 | Control  Baseline: 70+/-23 (8.6)  Week 8: 66+/-21 (8.2)  Week16: 64+/-20 (8.0)  Intervention  Baseline: 68+/-21 (8.4)  Week 8: 63+/-19 (7.9)  Week16: 60+/-18 (7.6) | **Week 8:** NS difference (p=0.6189, 95%CI -0.78, 1.30)*  **Week 16:** NS difference (p=0.3940, 95%CI -0.58, 1.44)* | **Vitamin C (mmol/L)**  Control  Baseline: 0.003+/-0.002  Week 8: 0.003+/-0.002  Week 16: 0.003+/-0.003  Intervention  Baseline: 0.003+/-0.002  Week 8: 0.01+/-0.005  Week 16: 0.01+/-0.005  **Vitamin E (mmol/L)**  Control  Baseline: 0.05+/-0.02  Week 8: 0.05+/-0.02  Week 16: 0.04+/-0.02  Intervention  Baseline: 0.05+/-0.02  Week 8: 0.06+/-0.02  Week 16: 0.07+/-0.02  **Se (umol/L)**  Control  Baseline: 0.51+/-.013  Week 8: 0.46+/-0.14  Week 16: 0.43+/-0.14  Intervention  Baseline: 0.47+/-0.12  Week 8: 0.53+/-0.12  Week 16: 0.58+/-0.12 | **Vitamin C:**  **Week 8:** Significant ↑ favouring intervention (p=0.0000, 95%CI -0.01, -0.00)*  **Week 16:** Significant ↑ favouring intervention (p=0.0000, 95%CI -0.01, -0.00)*  **Vitamin E:**  **Week 8:** NS difference (p=0.0835, 95%CI -0.02, 0.00)*  **Week 16:** Significant ↑ favouring intervention (p=0.0000, 95%CI -0.04, -0.02)*  **Selenium:**  **Week 8:** NS difference (p=0.0637, 95%CI -0.14, 0.00)*  **Week 16:** Significant ↑ favouring intervention (p=0.0002, 95%CI -0.22, -0.08)* |
| Yarahmadi 2021,  Iran, Vitamin E and C | Control:  Baseline FBS: 137.8+/-44.5  Week 8 FBS: 142.8+/-49.4  Change FBS: 10.6+/-26.6  Intervention:  Baseline FBS: 132.7+/-38.7  Week 8 FBS: 131.2+/-35.7  Change FBS: -13.0+/-42.2 | NS difference (p=0.82) | NR | NR | Control:  Baseline HbA1c: 9.5+/-1.6  Week 8 HbA1c: 9.6+/-1.7  Change HbA1c: 0.2+/-2.46  Intervention:  Baseline HbA1c: 9.8+/-1.7  Week 8 HbA1c: 9.0+/-1.8  Change HbA1c: -1.1+/-1.0 | Control:  Baseline HbA1c: 80+/-18(9.5)  Week 8 HbA1c: 81+/-19(9.6)  Intervention:  Baseline HbA1c: 84+/-19(9.8)  Week 8 HbA1c: 75+/-20(9.0) | NS difference (p=0.56) | NR | NR |
| Das 2022,  India, Amino acids | NR | NR | **At inclusion**  **Random Blood Sugar (units NR)**  Control: 178.2  Intervention: 180.87 | NR | **At inclusion**  **HbA1c (%)**  Control: 7.74  Intervention: 7.92 | **At inclusion**  **HbA1c**  Control: 61 (7.7)  Intervention: 63(7.9) | NR | NR | NR |
| Armstrong 2014,  USA, Europe and Taiwan, Arginine, glutamine and HMB | NR | NR | NR | NR | NR | NR | NR | NR | NR |
| Eneroth 2004,  Sweden, Fortimel | NR | NR | **At inclusion**  **Glucose (mmol/l)**  Median(range)  Control: 8.5(10.7)  Intervention: 8.3(8.8) | NR | **At inclusion**  **HbA1c (%)**  Median(range)  Control: 7.0(5.2)  Intervention: 7.1(5.4) | Median  Control: 53+/- (7.0)  Intervention: 54+/- (7.1) | NR | NR | NR |
| Yanes-Quesada  2021,  Cuba, Diamel | NR | NR | **Blood Glucose (mmol/L)**  **Control**  Baseline: 9.5+/-3.7  12 months later: 9.5+/-3.7  **Intervention**  Baseline: 10.7+/-3.8^  12 months later: 8.2+/-2.5 | **Blood Glucose (mmol/L)**  Baseline  NR  12 months later  NS (p=0.064) | **Control**  Baseline: 7.9+/-1.6  12 months later: 7.9+/-1.68  **Intervention**  Baseline: 8.1+/-1.6  12 months later: 7.2+/-1.28 | **Control**  Baseline: 63+/-18(7.9)  12 months later: 63+/-18(7.9)  **Intervention**  Baseline: 65+/-18(8.1)  12 months later: 55+/-14.0(7.2) | Baseline  NR  12 months later  Significant ↓ favouring intervention (p=0.025) | NR | NR |
| **Nutrition education (Reported outcome n=2))** | | | | | | | | | |
| Basiri 2020,  USA, Dietitian and Boost Glucose Control supplement | NR | NR | NR | NR | **HbA1c (%)**  Control:  Baseline: 8.40% (68 mmol/mol)  Week 12: 8.01% (65 mmol/mol)  Intervention:  Baseline: 7.95% (63 mmol/mol)  Week 12: 7.67% (60 mmol/mol) | Baseline: 68 (8.4)  Week 12: 65 (8.0)  Intervention:  Baseline: 63 (8.0)  Week 12: 60 (7.7) | NR and unable to calculate due to no SD OR CI provided | NR | NR |
| Sung 2021,  Australia, MDT | NR | NR | NR | NR | Control:  Baseline  %: 7.55 (6.5-8.83,  n = 26)  mmol/mol:  59 (48-73)  5 months later: 7.2%  Intervention:  %: 8.10 (7.0-9.9,  n = 35)  mmol/mol:  65 (53-85)  5 months later: 8.5% | Control:  Baseline: 60(7.6)  5 months later: 55(7.2)  Intervention: 65(8.1)  5 months later: 69(8.5) | Baseline  NS difference (p=0.091)  5 months later  NS difference (p=0.11) | NR | NRV |
| Yang 2023,  China, Early nurse-led nutrition intervention | NR | NR | NR | NR | NR | NR | NR | NR | NR |
| Abbreviations  NS = Non-significant  NR = Not Reported  NA = Not Applicable  RD = Registered Dietitian  TG = Triglycerides  VLDL = Very Low-Density Lipoprotein  TC = Total Cholesterol  LDL = Low-Density Lipoprotein  HDL = High-Density Lipoprotein  SD = Standard Deviation  CI = Confidence Intervals   1. Values are adjusted for baseline values of each biochemical variable, age and baseline BMI. 2. Values are adjusted for baseline values of each biochemical variable.   ^The worst result was selected in order to not misrepresent the data, as different tables reported different results. Nil email found for authors on the published paper.  *= between group differences calculated from individual group summary statistics  *Note:* results non-adjusted unless specified  *Note:* A calculated conversion completed for studies that report HbA1c in mg/dl as per journal author guidelines | | | | | | | | | |
