# Supplementary material for: Effectiveness of dietary interventions in individuals with diabetes for preventing and healing chronic wounds; a systematic review with meta‐analysis
Source: Diabet Med. 2025 Jul 9;42(9):e70100. doi: 10.1111/dme.70100 (PMC12352720; doi:10.1111/dme.70100)
Supplement: Supplementary file 1 — Data S1. [file DME-42-e70100-s001.zip › dme70100-sup-0011-TableS6.docx]

| **Supplementary Table 6. Secondary outcome lipid marker measures and other measured including between-group difference reported in studies investigating the effectiveness of nutrition interventions for individuals with diabetes-related foot ulceration.** | | | | | | | | | | | | |
| --- | --- | --- | --- | --- | --- | --- | --- | --- | --- | --- | --- | --- |
| **Reference, country** | **TG (mg/dl)** | **TG**  **between group difference at follow-up** | **VLDL (mg/dl)** | **VLDL**  **between group difference at follow-up** | **TC (mg/dl)** | **TC between group difference at follow-up** | **LDL (mg/dl)** | **LDL between group difference at follow-up** | **HDL (mg/dl)** | **HDL between group difference at follow-up** | **Other** | **Other between group difference at follow-up** |
| **Single nutrient supplement studies (Report outcome n=9)** | | | | | | | | | | | | |
| Bashmakov 2014,  Egypt, Trans-resveratrol | NR | NR | NR | NR | Control: Mean within group changes (95%CI): 32.60 (4.29, 60.91) (SD 45.68*)  Intervention: Mean within group changes (95%CI): 17.29 (3.52, 31.05) (SD 26.28*) | NS difference (p=0.3092, 95%CI -15.19, 45.81)* | Control: Mean within group changes (95%CI): 23.80 (−1.36, 48.96) (SD 40.59*)  Intervention: Mean within group changes (95%CI): 20.0 (6.2, 33.8) (SD 26.34*) | NS difference (p=0.7830, 95%CI -24.47, 32.07)* | Control: Mean within group changes (95%CI): 3.40 (−0.11, 6.91) (SD 5.66*)  Intervention: Mean within group changes (95%CI): -1.43 (−6.27, 3.42) (SD 9.25*) | NS difference (p=0.1579, 95%CI -2.02, 11.68)* | **Plasma fibrinogen (mg/dl)**  Control: Mean within group changes (95%CI): 20.70 (−235.48, 276.88) (SD 143.32*)  Intervention: Mean within group changes (95%CI): 118.93 (12.29, 225.56) (SD 203.57*) | **Plasma fibrinogen (mg/dl)**  NS difference (p=0.2043, 95%CI -253.96, 57.50)* |
| Gunton 2021,  Australia, Vitamin C | NR | NR | NR | NR | NR | NR | NR | NR | NR | NR | NR | NR |
| Halschou-Jensen 2021,  Denmark, Vitamin D | NR | NR | NR | NR | NR | NR | NR | NR | NR | NR | NR | NR |
| Kamble 2020,  India, Vitamin D | Control  Baseline: 159.4+/-68.8  End of trial: 155.9+/-70.5  Change: 3.8+/-6.4  Intervention  Baseline: 169.6+/-54.4  End of trial: 150.7+/-50.3  Change: 18.8+/-51.6 | NS difference (p=0.126) | NR | NR | Control  Baseline: 172.4+/-38.2  End of trial: 179.2+/-40.6  Change: -6.6+/-2.2  Intervention  Baseline: 180.8+/-30.3  End of trial: 165.5+/-30.7  Change: 15.1+/-0.07 | Significant ↓ favouring intervention (p=0.0001) | NR | NR | Control  Baseline: 37.6+/-4.6  End of trial: 32.6+/-5.1  Change: 5.1+/-1.3  Intervention  Baseline: 34.8+/-3.8  End of trial: 36.1+/-2.4  Change: -1.3+/-1.2 | Significant ↑ favouring intervention (p=0.0001) | NR | NR |
| Mozaffari-Khosravi 2016,  Iran, Vitamin D | NR | NR | NR | NR | NR | NR | NR | NR | NR | NR | NR | NR |
| Rangabashyam 2020,  India, Vitamin D | NR | NR | NR | NR | NR | NR | NR | NR | NR | NR | NR | NR |
| Razzaghi 2017,  Iran, Vitamin D | **Adjusted^a^**  Control: -2.4+/-5.7  Intervention: -16.0+/-5.7 | NS difference (p=0.09) | **Adjusted^a^**  Control: -0.5+/-1.1  Intervention: -3.2+/-1.1 | NS difference (p=0.09) | **Adjusted^a^**  Control: 3.4+/-4.7  Intervention: -13.9+/-4.7 | Significant ↓ favouring intervention (p=0.01) | **Adjusted^a^**  Control: 0.3+/-4.4  Intervention: -15.3+/-4.4 | Significant ↓ favouring intervention (p=0.01) | **Adjusted^a^**  Control: 3.4+/-0.9  Intervention: 4.9+/-0.9 | NS difference (p=0.25) | **Adjusted^a^**  **Total-/HDL-cholesterol ratio**  Control: -0.2+/-0.2  Intervention: -1.1+/-0.2 | Significant ↓ favouring intervention (p=0.003) |
| Jain 2012,  India, Vitamin E | NR | NR | NR | NR | **Total Cholesterol (mg%)**  Primary prevention (w/o complications)  Type 1 diabetes (w/o complications) control group  Baseline: 202.5+/-10.14  12 months: 200.2+/-8.88  18 months: 201+/-+/-8.41  24 months: 202+/-7.36  Type 1 diabetes (w/o complications) intervention group  Baseline: 205+/-13.22  12 months: 202.8+/-14.54  18 months: 199.5+/-14.84  24 months: 195.2+/-16.57  Type 2 diabetes w/o complications) control group  Baseline: 206.45+/-8.19  12 months: 208.05+/-8.08  18 months: 208.95+/-7.65  24 months: 210+/-7.68  Type 2 diabetes (w/o complications) intervention group  Baseline: 207.65+/-10.67  12 months: 205.85+/-12.34  18 months: 205+/-11.43  24 months: 204+/-11.18  Secondary prevention  Type 1 diabetes w/ complications control group  Baseline: 206.4+/-8.09  12 months: 206+/-7.54  18 months: 207+/-7.53  24 months: 208+/-8.86  Type 1 diabetes w/ complications intervention group  Baseline: 206.8+/-10.07  12 months: 205.8+/-9.1  18 months: 205+/-8.68  24 months: 204.5+/-4.33*  Type 2 diabetes w/ complications control group  Baseline: 209.8+/-9.69  12 months: 210.7+/-10  18 months: 212+/-+/-10.68  24 months: 212.8+/-10.34  Type 2 diabetes w/ complications intervention group  Baseline: 211+/-13.01  12 months: 211.8+/-14.4  18 months: 209.0+/-9.98  24 months: 208+/-12.04* | **Primary prevention (w/o complications)**  **Type 1 diabetes (w/o complications)**  **12 months:** NS difference (p=0.3913, 95%CI -8.62, 3.42)*  **18 months:** NS difference (p=0.6206, 95%CI -4.53, 7.53)*  **24 months:** Significant ↓ favouring intervention (p=0.0379, 95%CI 0.39, 13.21)*  **Type 2 diabetes w/o complications)**  **12 months:** NS difference (p=0.4021, 95%CI -3.01, 7.41)*  **18 months:** NS difference (p=0.1093, 95%CI -0.91, 8.81)*  **24 months:** Significant ↓ favouring intervention (p=0.0150, 95%CI 1.21, 10.79)*  **Secondary prevention**  **Type 1 diabetes w/ complications**  **12 months:** NS difference (p= 0.9099, 95%CI -3.30, 3.70)*  **18 months:** NS difference (p=0.2461, 95%CI -1.40, 5.40)*  **24 months:** Significant ↓ favouring intervention (p=0.0194, 95%CI 0.58, 6.42)*  **Type 2 diabetes w/ complications**  **12 months:** NS difference (p=0.6749, 95%CI -6.29, 4.09)*  **18 months:** NS difference (p=0.1721, 95%CI -1.33, 7.33)*  **24 months:** Significant ↓ favouring intervention (p=0.0455, 95%CI 0.10, 9.50)* | NR | NR | NR | NR | NR | NR |
| Mohseni 2018,  Iran, Probiotic | Control: 2.1+/-3.6  Intervention: -3.5+/-3.6 | NS difference (p=0.28) | Control: 0.4+/-0.7  Intervention: -0.7+/-0.7 | NS difference (p=0.28) | Control: 6.2+/-4.1  Intervention: -4.1+/-4.1 | NS difference (p=0.08) | Control: 3.7+/-3.8  Intervention: -5.3+/-3.8 | NS difference (p=0.10) | Control: 2.5+/-0.7  Intervention: 1.5+/-0.7 | NS difference (p=0.33) | NR | NR |
| Mokhtari 2020,  Iran, Nanocurcumin | **Adjusted^b^**  Control  Baseline: 145.7+/-45.5  Week 12: 147.7+/-47.0  Intervention  Baseline: 140.6+/-75.3  Week 12: 138.9+/-72.6 | NS difference (p=0.25, B(95%CI): -3.94(-10.80, 2.92) | **Adjusted^b^**  Control  Baseline: 29.1+/-9.1  Week 12: 29.5+/-9.4  Intervention  Baseline: 28.1+/-15.1  Week 12: 27.8+/-14.5 | NS difference (p=0.25, B(95%CI): -0.78(-2.16, 0.58)) | **Adjusted^b^**  Control  Baseline: 160.2+/-42.3  Week 12: 165.0+/-44.2  Intervention  Baseline: 157.1+/-38.2  Week 12: 148.8+/-33.4 | Significant ↓ favouring intervention (p<0.001, B(95%CI): -13.41(-19.83, -6.98)) | **Adjusted^b^**  Control  Baseline: 91.2+/-39.4  Week 12: 96.2+/-41.2  Intervention  Baseline: 87.9+/-29.0  Week 12: 79.9+/-22.0 | Significant ↓ favouring intervention (p<0.001, B(95%CI): -14.03(-21.08, -6.97)) | **Adjusted^b^**  Control  Baseline: 39.8+/-4.8  Week 12: 39.3+/-5.1  Intervention  Baseline: 41.1+/-7.9  Week 12: 41.8+/-9.1 | NS difference (p=0.23, B(95%CI): 1.20(-0.80, 3.22)) | NR | NR |
| Momen-Heravi 2017,  Iran, Zinc | **Adjusted Change^a^**  Control: -2.1+/-5.6  Intervention: -10.9+/-5.6 | NS difference (p=0.27) | **Adjusted Change^a^**  Control: -0.4+/-1.1  Intervention: -2.2+/-1.1 | NS difference (p=0.27) | **Adjusted Change^a^**  Control: -1.7+/-4.6  Intervention: -9.6+/-4.6 | NS difference (p=0.24) | **Adjusted Change^a^**  Control: -1.8+/-4.4  Intervention: -12.1+/-4.4 | NS difference (p=0.10) | **Adjusted Change^a^**  Control: 1.1+/-0.9  Intervention: 4.1+/-0.9 | Significant ↑ favouring intervention (p=0.01) | **Adjusted Change^a^**  **Total Cholesterol/HDL ratio (mg/dl)**  Control: -0.1+/-0.2  Intervention: -0.8+/-0.2 | Significant ↓ favouring intervention (p=0.01) |
| Razzaghi 2018,  Iran, Magnesium | **Adjusted^a^**  Control: -3.4+/-9.3  Intervention: -10.9+/-9.3 | NS difference (p=0.57) | **Adjusted^a^**  Control: -0.7+/-1.9  Intervention: -2.2+/-1.9 | NS difference (p=0.57) | **Adjusted^a^**  Control: 5.7+/-6.6  Intervention: 5.2+/-6.6 | NS difference (p=0.95) | **Adjusted^a^**  Control: 4.7+/-5.7  Intervention: 5.1+/-5.7 | NS difference (p=0.96) | **Adjusted^a^**  Control: 1.8+/-1.4  Intervention: 2.1+/-1.4 | NS difference (p=0.86) | NR | NR |
| Soleimani 2017,  Iran, Omega-3 | **Adjusted^a^**  Control: 0.7+/-7.5  Intervention: -15.5+/-7.5 | NS difference (p=0.13) | **Adjusted^a^**  Control: 0.1+/-1.5  Intervention: -3.1+/-1.5 | NS difference (p=0.13) | **Adjusted^a^**  Control: 9.7+/-5.5  Intervention: 2.7+/-5.5 | NS difference (p=0.38) | **Adjusted^a^**  Control: 5.8+/-5.1  Intervention: 4.4+/-5.1 | NS difference (p=0.83) | **Adjusted^a^**  Control: 3.7+/-1.0  Intervention: 1.5+/-1.0 | NS difference (p=0.11) | NR | NR |
| **Multi-nutrient supplement studies (Reported outcome n=6)** | | | | | | | | | | | | |
| Afzali 2019,  Iran, Mg and vitamin E | **Adjusted^a^**  Control  Baseline: 139.5+/-38.9  Week 12:137.1+/-45.4  Intervention  Baseline: 156.5+/-40.6  Week 12: 141.7+/-36.5 | Significant ↓ favouring intervention  (p=0.04, B(95%CI): -10.08 (-19.70, -0.46)) | **Adjusted^a^**  Control  Baseline: 27.9+/-8.0  Week 12: 27.4+/-9.1  Intervention  Baseline: 31.3+/-8.1  Week 12: 28.3+/-7.3 | Significant ↓ favouring intervention  (p=0.04, B(95%CI): -2.01 (-3.94, -0.09)) | **Adjusted^a^**  Control  Baseline: 154.8+/-40.7  Week 12: 151.6+/-42.1  Intervention  Baseline: 166.7+/-50.5  Week 12: 156.8+/-43.4 | NS difference  (p=0.14, B(95%CI): -4.88 (-11.47, 1.70)) | **Adjusted^a^**  Control  Baseline: 91.6+/-35.7  Week 12: 88.4+/-37.0  Intervention  Baseline: 100.8+/-47.7  Week 12: 90.7+/-41.3 | Significant ↓ favouring intervention  (p=0.03, B(95%CI): -5.88 (-11.42, -0.34)) | **Adjusted^a^**  Control  Baseline: 35.4+/-4.9  Week 12: 35.7+/-6.1  Intervention  Baseline: 34.5+/-9.5  Week 12: 37.7+/-6.8 | Significant ↑ favouring intervention  (p=0.01, B(95%CI): 2.62 (0.60, 4.63)) | NR | NR |
| Bosede 2012,  Nigeria, Vitamin E, C and selenium | NR | NR | NR | NR | NR | NR | NR | NR | NR | NR | NR | NR |
| Yarahmadi 2021,  Iran, Vitamin E and C | Control  Baseline: 104.9+/-22.2  Week 8: 118.8+/-29.7  Change: 18.2+/-24.7  Intervention  Baseline: 129.4+/-86.3  Week 8: 139.5+/-31.2  Change: 3.4+/-95.3 | NS difference (p=0.17) | Control  Baseline: 20.8+/-4.5  Week 8: 23.7+/-6.1  Change: 3.8+/-4.9  Intervention  Baseline: 25.8+/-17.1  Week 8: 27.7+/-6.2  Change: -0.7+/-19.1 | NS difference (p=0.16) | Control  Baseline: 141.6+/-33.9  Week 8: 167.7+/-38.5  Change: 20.7+/-18.3  Intervention  Baseline: 158.9+/-37.5  Week 8: 166.8+/-27.8  Change: 11.2+/-35.3 | NS difference (p=0.96) | Control  Baseline: 72.3+/-31.1  Week 8: 95.3+/-27.9  Change: 18.1+/-15.0  Intervention  Baseline: 81.5+/-26.1  Week 8: 93.6+/-23.6  Change: 14.8+/-28.9 | NS difference (p=0.99) | Control  Baseline: 43.4+/-19.9  Week 8: 42.3+/-14.8  Change: -2.6+/-14.0  Intervention  Baseline: 42.5+/-17.8  Week 8: 38.6+/-7.1  Change: 0.2+/-13.8 | NS difference (p=0.85) | **Total Cholesterol/HDL ratio (mg/dl)**  Control  Baseline: 3.5+/-1.1  Week 8: 4.1+/-1  Change: 0.5+/-1.1  Intervention  Baseline: 4.0+/-1.0  Week 8: 4.3+/-0.6  Change: 0.2+/-1.0 | NS difference (p=0.56) |
| Das 2022,  India, Amino acids | NR | NR | NR | NR | Control  Baseline: 171.67+/-36.33  Day 5: 178.33+/-38.39  Day 10: 183.07+/-33.41  Day 15: 183.14+/-32.89  Intervention  Baseline: 165.20+/-34.81  Day 5: 166.27+/-35.79  Day 10: 162.11+/-33.08  Day 15: 160.76+/-37.71 | Baseline  NS difference (p=0.48, t test: 0.49)  Day 5  NS difference (p=0.29, t test: 1.37)  Day 10  NS difference (p=0.13, t test: 2.13)  Day 15  NS difference (p=0.10, t test: 2.27) | Control  Baseline: 102.40+/-18.81  Day 5: 104.40+/-16.53  Day 10: 100.32+/-16.94  Day 15: 101.33+/-15.71  Intervention  Baseline: 99.82+/-17.01  Day 5: 96.27+/-16.13  Day 10: 96.18+/-18.59  Day 15: 95.16+/-15.51 | Baseline  NS difference (p=0.32, t test: 0.91)  Day 5  NS difference (p=0.58, t test: 0.30)  Day 10  NS difference (p=0.24, t test: 1.38)  Day 15  NS difference (p=0.12, t test: 2.31) | Control  Baseline: 35.17+/-12.99  Day 5: 36.07+/-8.70  Day 10: 36.32+/-8.88  Day 15: 36.02+/-9.09  Intervention  Baseline: 35.97+/-13.08  Day 5: 36.23+/-9.25  Day 10: 36.53+/-9.20  Day 15: 36.48+/-8.91 | Baseline  NS difference (p=0.41, t test: 0.69)  Day 5  NS difference (p=0.48, t test: 0.52)  Day 10  NS difference (p=0.36, t test: 0.81)  Day 15  NS difference (p=0.62, t test: 0.40) | **Creatinine (mg/dl)**  Control  Baseline: 1.46+/-1.09  Day 5: 1.24+/-1.24  Day 10: 1.16+/-1.13  Day 15: 1.07+/-1.38  Intervention  Baseline: 1.51+/-1.42  Day 5: 1.22+/-1.33  Day 10: 1.13+/-1.21  Day 15: 1.09+/-1.44  **Uric acid (mg/dl)**  Control  Baseline: 5.58+/-2.96  Day 5: 5.27+/-2.57  Day 10: 4.86+/-2.90  Day 15: 4.68+/-3.07  Intervention  Baseline: 6.03+/-2.53  Day 5: 5.50+/-2.64  Day 10: 5.10+/-2.84  Day 15:4.83+/-2.91 | **Creatinine**  Baseline  NS difference (p=0.61, t test: 0.26)  Day 5  NS difference (p=0.73, t test: 0.12)  Day 10  NS difference (p=0.71, t test: 0.14)  Day 15  NS difference (p=0.65, t test: 0.21)  **Uric acid**  Baseline  NS difference (p=0.29, t test: 1.16)  Day 5  NS difference (p=0.56, t test: 0.34)  Day 10  NS difference (p=0.32, t test: 0.71)  Day 15  NS difference (p=0.66, t test: 0.19) |
| Armstrong 2014,  USA, Europe and Taiwan, Arginine, glutamine and HMB | NR | NR | NR | NR | NR | NR | NR | NR | NR | NR | **Albumin (g/l) at baseline**  ≤40g/l = 127 participants | NR |
| Eneroth 2004,  Sweden, Fortimel | NR | NR | NR | NR | NR | NR | NR | NR | NR | NR | **At inclusion only - abnormal values**  **Albumin (Abnormal = <36g/l)**  **Transthyretin (Abnormal = <0.2g/l)**  N(%)  Control  Albumin: 6/25 (24%)  Transthyretin: 9/26(35%)  Intervention  Albumin: 6/21(29%)  Transthyretin: 4/20(20%)  **Creatinine (umol/l)**  Median(range)  Control: 91(140)  Intervention: 83(211) | NR |
| Yanes-Quesada  2021,  Cuba, Diamel | **(mmol/L)**  Control  Baseline: 2.8+/-2.4  1 year: 2.8+/-2.4  Intervention  Baseline: 2.3+/-0.9  1 year: 2.1+/-0.8 | NS Difference (p=0.069) | NR | NR | **(mmol/L)**  Control  Baseline: 5.3+/-0.6  1 year: 5.3+/-0.6  Intervention  Baseline: 5.3+/-0.9  1 year: 5.1+/-0.7 | NS Difference (p=0.375) | NR | NR | NR | NR | **Creatinine (mmol/L)**  Control  Baseline: 87.6+/-16.0  1 year: 87.2+/-11.5  Intervention  Baseline: 88.1+/-21.8^  1 year: 84.8+/-14.7 | NS Difference (p=0.360) |
| **Nutrition education (Reported outcome n=1))** | | | | | | | | | | | | |
| Basiri 2020,  USA, Dietitian and Boost Glucose Control supplement | NR | NR | NR | NR | NR | NR | NR | NR | NR | NR | NR | NR |
| Sung 2021,  Australia, MDT | NR | NR | NR | NR | NR | NR | NR | NR | NR | NR | NR | NR |
| Yang 2023,  China, Early nurse-led nutrition intervention | NR | NR | NR | NR | NR | NR | NR | NR | NR | NR | **Albumin (g/L)**  Control  Before: 35.01+/-5.44  After: 36.69+/-4.93  Intervention  Before: 35.48+/-5.11  After: 40.06+/-5.36  **Haemoglobin (g/L)**  Control  Before: 103.97+/-14.73  After: 108.36+/-15.71  Intervention  Before: 106.43+/-17.64  After: 118.24+/-18.91 | **Albumin**  Before  NS Difference (p=0.746)  After  Significant ↑ favouring intervention (p=0.013)  **Haemoglobin**  Before  NS Difference (p=0.545)  After  Significant ↑ favouring intervention (p=0.023) |
| Abbreviations  NS = Non-significant  NR = Not Reported  RD = Registered Dietitian  TG = Triglycerides  VLDL = Very Low-Density Lipoprotein  TC = Total Cholesterol  LDL = Low-Density Lipoprotein  HDL = High-Density Lipoprotein  SD = Standard Deviation  CI = Confidence Intervals   1. Values are adjusted for baseline values of each biochemical variable, age and baseline BMI. 2. Values are adjusted for baseline values of each biochemical variable.   ^The worst result was selected in order to not misrepresent the data, as different tables reported different results. Nil email found for authors on the published paper.  *= between group differences calculated from individual group summary statistics  *Note:* results non-adjusted unless specified  *Note:* A calculated conversion completed for studies that report HbA1c in mg/dl as per journal author guidelines | | | | | | | | | | | | |
