# Supplementary material for: Effectiveness of dietary interventions in individuals with diabetes for preventing and healing chronic wounds; a systematic review with meta‐analysis
Source: Diabet Med. 2025 Jul 9;42(9):e70100. doi: 10.1111/dme.70100 (PMC12352720; doi:10.1111/dme.70100)
Supplement: Supplementary file 1 — Data S1. [file DME-42-e70100-s001.zip › dme70100-sup-0012-TableS7.docx]

| **Supplementary Table 7. Secondary outcome inflammatory marker measures including between-group difference reported in studies investigating the effectiveness of nutrition interventions for individuals with diabetes-related foot ulceration.** | | | | | | | | | | | | | | |
| --- | --- | --- | --- | --- | --- | --- | --- | --- | --- | --- | --- | --- | --- | --- |
| **Reference, country** | **High-sensitivity C-reactive Protein (µg/mL)** | **High-sensitivity C-reactive Protein between group difference at follow-up** | **Erythrocyte Sedimentation Rate (mm/h)** | **Erythrocyte between group difference at follow-up** | **Nitric Oxide (µmol/L)** | **Nitric Oxide**  **between group difference at follow-up** | **Total Antioxidant Capacity (mmol/L)** | **Total Antioxidant Capacity between group difference at follow-up** | **Total Glutathione (GSH) (µmol/L)** | **Total Glutathione between group difference at follow-up** | **Malondialdehyde (µmol/L)** | **Malondialdehyde between group difference at follow-up** | **Other** | **Other between group difference at follow-up** |
| **Single nutrient supplement studies (Reported outcomes n=8)** | | | | | | | | | | | | | | |
| Bashmakov 2014,  Egypt, Trans-resveratrol | **C-reactive Protein (g/L**  Control  Baseline: 2.4(3.2)  Median within group changes (95%CI): -0.05 (−2.50, 1.40) (SD 3.15*)  Intervention  Baseline: 3.2(3.4)  Median within group changes (95%CI): -0.25 (−1.50, 1.50) (SD 2.86*) | NS difference (p=0.8728, 95%CI -2.36, 2.76)* | NR | NR | NR | NR | NR | NR | NR | NR | NR | NR | NR | NR |
| Gunton 2021,  Australia, Vitamin C | NR | NR | NR | NR | NR | NR | NR | NR | NR | NR | NR | NR | NR | NR |
| Halschou-Jensen 2021,  Denmark, Vitamin D | NR | NR | NR | NR | NR | NR | NR | NR | NR | NR | NR | NR | NR | NR |
| Kamble 2020,  India, Vitamin D | NR | NR | NR | NR | NR | NR | NR | NR | NR | NR | NR | NR | NR | NR |
| Mozaffari-Khosravi 2016,  Iran, Vitamin D | Control  Baseline: 7.3+/-2.2  After 4 weeks: 5.52+/-2.0  Change: -1.7+/-1.0  Intervention  Baseline: 7.25+/-2.85  After 4 weeks: 4.38+/-1.41  Change: -2.9+/-1.7 | Baseline  NS difference (p=0.71)  After 4 weeks  Significant ↓ favouring intervention (p=0.02)  Change  Significant ↓ favouring intervention (p=0.01) | Control  Baseline: 31.52+/-6.18  After 4 weeks: 20.52+/-6.11  Change: -11.0+/-3.1  Intervention  Baseline: 29.00+/-6.97  After 4 weeks: 16.37+/-5.31  Change: -12.6+/-3.2 | Baseline  NS difference (p=0.46)  After 4 weeks  Significant ↓ favouring intervention (p=0.01)  Change  NS difference (p=0.08) | NR | NR | NR | NR | NR | NR | NR | NR | NR | NR |
| Rangabashyam 2020,  India, Vitamin D | NR | NR | NR | NR | NR | NR | NR | NR | NR | NR | NR | NR | NR | NR |
| Razzaghi 2017,  Iran, Vitamin D | **Adjusted^a^**  Control: 2.2+/-0.6  Intervention: -0.7+/-0.6 | Significant ↓ favouring intervention (p=0.001) | **Adjusted^a^**  Control: -19.1+/-3.7  Intervention: -33.6+/-3.7 | Significant ↓ favouring intervention (p=0.008) | **Adjusted^a^**  Control: 2.8+/-1.0  Intervention: 3.3+/-1.0 | NS difference (p=0.75) | **Adjusted^a^**  Control: -99.1+/-32.1  Intervention: -90.6+/-32.1 | NS difference (p=0.85) | **Adjusted^a^**  Control: -44.7+/-16.3  Intervention: -31.3+/-16.3 | NS difference (p=0.56) | **Adjusted^a^**  Control: -0.3+/-0.1  Intervention: -0.7+/-0.1 | Significant ↓ favouring intervention (p=0.01) | NR | NR |
| Jain 2012,  India, Vitamin E | NR | NR | NR | NR | NR | NR | NR | NR | NR | NR | NR | NR | NR | NR |
| Mohseni 2018,  Iran, Probiotic | **Adjusted Change^a^**  Control: -2.2+/-2.0  Intervention: -8.5+/-2.0 | Significant ↓ favouring intervention (p=0.02) | **Adjusted Change^a^**  Control: -10.0+/-2.2  Intervention: -11.9+/-2.2 | NS difference (p=0.56) | **Adjusted Change^a^**  Control: 0.5+/-1.5  Intervention: 6.4+/-1.5 | Significant ↑ favouring intervention (p=0.01) | **Adjusted Change^a^**  Control: -96.8+/-27.9  Intervention: 191.0+/-27.9 | Significant ↑ favouring intervention (p <0.001) | **Adjusted Change^a^**  Control: 28.2+/-22.8  Intervention: 44.8+/-22.8 | NS difference (p=0.61) | **Adjusted Change^a^**  Control: -0.3+/-0.1  Intervention: -0.7+/-0.1 | Significant ↓ favouring intervention (p=0.008) | NR | NR |
| Mokhtari 2020,  Iran, Nanocurcumin | **Adjusted^b^**  Control  Baseline: 52.8+/-27.0  Week 12: 22.1+/-17.5  Intervention  Baseline: 48.5+/-27.0  Week 12: 15.4+/-16.3 | NS difference (p=0.17, B(95%CI): -4.97(-12.27, 2.32)) | **Adjusted^b^**  Control  Baseline: 60.6+/-27.2  Week 12: 23.4+/-13.3  Intervention  Baseline: 59.4+/-22.6  Week 12: 22.3+/-18.2 | NS difference (p=0.87, B(95%CI): -0.58(-7.78, 6.61)) | **Total Nitrite (µmol/L)**  **Adjusted^b^**  Control  Baseline: 46.9+/-5.3  Week 12: 46.6+/-6.2  Intervention  Baseline: 43.2+/-5.3  Week 12: 44.4+/-4.6 | NS difference (p=0.28, B(95%CI): 0.99(-0.86, 2.85)) | **Adjusted^b^**  Control  Baseline: 1277.9+/-248.2  Week 12: 1263.4+/-229.5  Intervention  Baseline: 1396.6+/-266.9  Week 12: 1499.1+/-249.1 | Significant ↑ favouring intervention (p<0.001, B(95%CI): 140.03(69.52, 210.55)) | **Adjusted^b^**  Control  Baseline: 760.6+/-255.1  Week 12: 736.1+/-230.1  Intervention  Baseline: 915.7+/-382.1  Week 12: 948.8+/-350.5 | Significant ↑ favouring intervention (p=0.01, B(95%CI): 80.22(18.30, 142.14)) | **Adjusted^b^**  Control  Baseline: 2.4+/-0.3  Week 12: 2.4+/-0.3  Intervention  Baseline: 2.5+/-0.3  Week 12: 2.4+/-0.2 | NS difference (p= 0.14, B(95%CI): -0.06(-0.15, 0.02)) | NR | NR |
| Momen-Heravi 2017,  Iran, Zinc | **Adjusted^a^**  Control: -7.4+/-3.9  Intervention: -19.8+/-3.9 | Significant ↓ favouring intervention (p=0.02) | **Adjusted^a^**  Control: -16.1+/-5.3  Intervention: -31.7+/-5.3 | Significant ↓ favouring intervention (p=0.04) | **Adjusted^a^**  Control: -1.1+/-1.3  Intervention: 9.4+/-1.3 | Significant ↑ favouring intervention (p<0.001) | **Adjusted^a^**  Control: -83.6+/-32.5  Intervention: 63.4+/-32.5 | Significant ↑ favouring intervention (p=0.003) | **Adjusted^a^**  Control: -30.9+/-18.5  Intervention: 64.0+/-18.5 | Significant ↑ favouring intervention (p=0.001) | **Adjusted^a^**  Control: -0.2+/-0.1  Intervention: -0.7+/-0.1 | Significant ↓ favouring intervention (p=0.03) | NR | NR |
| Razzaghi 2018,  Iran, Magnesium | **Adjusted Change^a^**  Control: -3.5+/-3.3  Intervention: -21.0+/-3.3 | Significant ↓ favouring intervention (p<0.001) | **Adjusted Change^a^**  Control: -6.5+/-3.1  Intervention: -10.7+/-3.1 | NS difference (p=0.35) | **Adjusted Change^a^**  Control: -0.8+/-0.8  Intervention: -0.5+/-0.8 | NS difference (p=0.79) | **Adjusted Change^a^**  Control: -95.7+/-25.7  Intervention: -27.8+/-25.7 | NS difference (p=0.09) | **Adjusted Change^a^**  Control: -10.4+/-16.5  Intervention: -14.9+/-16.5 | NS difference (p=0.84) | **Adjusted Change^a^**  Control: 0.1+/-0.1  Intervention: -0.3+/-0.1 | Significant ↓ favouring intervention (p=0.01) | NR | NR |
| Soleimani 2017,  Iran, Omega-3 | **Adjusted Change^a^**  Control: -6.9+/-3.1  Intervention: -26.8+/-3.1 | Significant ↓ favouring intervention (p<0.001) | NR | NR | **Adjusted Change^a^**  Control: 0.3+/-1.2  Intervention: 1.7+/-1.2 | NS difference (p=0.42) | **Adjusted Change^a^**  Control: -67.6+/-29.6  Intervention: 77.7+/-29.6 | Significant ↑ favouring intervention (p=0.001) | **Adjusted Change^a^**  Control: -6.7+/-19.0  Intervention: 51.9+/-19.0 | Significant ↑ favouring intervention (p=0.03) | **Adjusted Change^a^**  Control: 0.1+/-0.1  Intervention: -0.2+/-0.1 | NS difference (p=0.15) | NR | NR |
| **Multi-nutrient supplement studies (Reported outcomes n=4)** | | | | | | | | | | | | |  |  |
| Afzali 2019,  Iran, Mg and vitamin E | **Adjusted^a^**  Control  Baseline: 13.9+/-4.9  Week 12: 13.6+/-5.0  Intervention  Baseline: 10.2+/-5.0  Week 12: 6.9+/-4.2 | Significant ↓ favouring intervention (p<0.001, B(95%CI): -3.42 (-4.44, -2.41)) | **Adjusted^a^**  Control  Baseline: 30.8+/-17.2  Week 12: 26.7+/-17.5  Intervention  Baseline: 23.8+/-18.1  Week 12: 8.3+/-5.8 | Significant ↓ favouring intervention (p<0.001, B(95%CI): -14.54 (-19.73, -9.35)) | **Adjusted^a^**  Control  Baseline: 45.3+/-3.5  Week 12: 45.8+/-4.5  Intervention  Baseline: 46.9+/-1.8  Week 12: 48.3+/-1.7 | NS difference (p=0.06, B(95%CI): 1.23 (-0.09, 2.56)) | **Adjusted^a^**  Control  Baseline: 1163.0+/-136.3  Week 12: 1106.8+/-153.0  Intervention  Baseline: 1108.1+/-68.5  Week 12: 1118.7+/-73.0 | Significant ↑ favouring intervention (p=0.03, B(95%CI): 53.61 (4.65, 102.57)) | **Adjusted^a^**  Control  Baseline: 565.7+/-95.9  Week 12: 550.4+/-75.7  Intervention  Baseline: 624.2+/-116.2  Week 12: 611.7+/-96.6 | NS difference (p=0.13, B(95%CI): 25.34 (-8.03, 58.72)) | **Adjusted^a^**  Control  Baseline: 3.2+/-0.7  Week 12: 3.1+/-0.6  Intervention  Baseline: 2.8+/-0.2  Week 12: 2.5+/-0.2 | Significant ↓ favouring intervention (p<0.001, B(95%CI): -0.30 (-0.45, -0.15)) | NR | NR |
| Bosede 2012,  Nigeria, Vitamin E, C and selenium | NR | NR | NR | NR | NR | NR | Control  Baseline: 0.67+/-0.15  Week 8: 0.64+/-0.13  Week16: 0.61+/-0.14  Intervention  Baseline: 0.65+/-0.10  Week 8: 0.69+/-0.09  Week16: 0.72+/-0.08 | **Week 8:** NS difference (p=0.1204, 95%Ci -0.11, 0.01)*  **Week 16:** Significant ↑ favouring intervention (p=0.0013, 95%CI -0.17, -0.05)* | NR | NR | NR | NR | **Lipid peroxide (LPO) (umol/L)**  Control  Baseline: 50.41+/-8.85  Week 8: 54.67+/-13.24  Week16: 59.85+/-13.45  Intervention  Baseline: 62.28+/-7.36  Week 8: 54.49+/-8.06  Week16: 48.27+/-10.15  **8-hydroxy-2’-deoxyguanosine (9-OHdG) (ng/ml)**  Control  Baseline: 45.23+/-7.78  Week 8: 52.15+/-9.98  Week16: 58.80+/-11.91  Intervention  Baseline: 53.51+/-5.23  Week 8: 47.93+/-8.38  Week16: 41.48+/-9.82  **Superoxide Dismutase (U/g Hb)**  Control  Baseline: 4214.33+/-644.93  Week 8: 4009.28+/-513.51  Week16: 3789.33+/-469.20  Intervention  Baseline: 4123.99+/-634.43  Week 8: 4128.62+/-632.42  Week16: 4239.29+/-669.45  **Glutathione Peroxidase (GPx) (U/g Hb)**  Control  Baseline: 1237.99+/-184.26  Week 8: 3245.38+/-3349.87  Week16: 3159.66+/-3329.29  Intervention  Baseline: 1325.28+/-282.90  Week 8: 2307.26+/-2559.90  Week16: 2853.99+/-3434.48 | **LPO**  **Week 8:** NS difference (p=0.9539, 95%Ci -6.05, 6.41)*  **Week 16:** Significant ↓ favouring intervention (p=0.0012, 95%CI 4.80, 18.36)*  **9-OHdG**  **Week 8:** NS difference (p=0.1120, 95%CI -1.02, 9.46)*  **Week 16:** Significant ↓ favouring intervention (p=0.0000, 95%CI 11.11, 23.53)*  **Superoxide Dismutase**  **Week 8:** NS difference (p=0.4674, 95%CI -446.93, 208.25)*  **Week 16:** Significant ↑ favouring intervention (p=0.0083, 95%CI -778.70, -121.22)*  **GPx**  **Week 8:** NS difference (p=0.2714, 95%CI -757.25, 2633.49)*  **Week 16:** NS difference (p=0.7507, 95%CI -1617.82, 2229.16)* |
| Yarahmadi 2021,  Iran, Vitamin E and C | Control  Baseline: 26.6+/-29.1  Week 8: 21.8+/-27.2  Change: -4.8+/-4.5  Intervention  Baseline: 29.8+/-33.9  Week 8: 5.9+/-6.6  Change: -26.1+/-28.8 | Significant ↓ favouring intervention (p=0.04) | Control  Baseline: 41.2+/-35.7  Week 8: 32.4+/-29.5  Change: -4.8+/-10.7  Intervention  Baseline: 54.6+/-41.5  Week 8: 16.4+/-12.2  Change: -38.1+/-34.6 | Significant ↓ favouring intervention (p=0.002) | NR | NR | NR | NR | NR | NR | NR | NR | **Prooxidant-Antioxidant Balance (HK unit)**  Control  Baseline: 166.6+/-46.3  Week 8: 177.3+/-26  Change: 2.6+/-31.4  Intervention  Baseline: 172.2+/-50.4  Week 8: 113.4+/-53.2  Change: -54.1+/-35.6 | Significant ↓ favouring intervention (p=0.04) |
| Das 2022,  India, Amino acids | NR | NR | NR | NR | NR | NR | NR | NR | NR | NR | NR | NR | NR | NR |
| Armstrong 2014,  USA, Europe and Taiwan, Arginine, glutamine and HMB | NR | NR | NR | NR | NR | NR | NR | NR | NR | NR | NR | NR | NR | NR |
| Eneroth 2004,  Sweden, Fortimel | **At inclusion**  **CRP(mg/l)**  Median(range)  Control: 7(5)  Intervention: 6(67) | NR | **At inclusion**  **Erythrocyte volume fraction(%)**  Median(range)  Control: 41(19)  Intervention: 40(17) | NR | NR | NR | NR | NR | NR | NR | NR | NR | NR | NR |
| Yanes-Quesada  2021,  Cuba, Diamel | NR | NR | NR | NR | NR | NR | NR | NR | NR | NR | NR | NR | NR | NR |
| **Nutrition education (Reported outcomes n=0)** | | | | | | | | | | | | |  |  |
| Basiri 2020,  USA, Dietitian and Boost Glucose Control supplement | NR | NR | NR | NR | NR | NR | NR | NR | NR | NR | NR | NR | NR | NR |
| Sung 2021,  Australia, MDT | NR | NR | NR | NR | NR | NR | NR | NR | NR | NR | NR | NR | NR | NR |
| Yang 2023,  China, Early nurse-led nutrition intervention | NR | NR | NR | NR | NR | NR | NR | NR | NR | NR | NR | NR | NR | NR |
| Abbreviations  NS = Non-significant  NR = Not Reported  RD = Registered Dietitian  TG = Triglycerides  VLDL = Very Low-Density Lipoprotein  TC = Total Cholesterol  LDL = Low-Density Lipoprotein  HDL = High-Density Lipoprotein  SD = Standard Deviation  CI = Confidence Intervals   1. Values are adjusted for baseline values of each biochemical variable, age and baseline BMI. 2. Values are adjusted for baseline values of each biochemical variable.   ^The worst result was selected in order to not misrepresent the data, as different tables reported different results. Nil email found for authors on the published paper.  *= between group differences calculated from individual group summary statistics  *Note:* results non-adjusted unless specified  *Note:* A calculated conversion completed for studies that report HbA1c in mg/dl as per journal author guidelines | | | | | | | | | | | | | | |
