# Supplementary material for: Effectiveness of dietary interventions in individuals with diabetes for preventing and healing chronic wounds; a systematic review with meta‐analysis
Source: Diabet Med. 2025 Jul 9;42(9):e70100. doi: 10.1111/dme.70100 (PMC12352720; doi:10.1111/dme.70100)
Supplement: Supplementary file 1 — Data S1. [file DME-42-e70100-s001.zip › dme70100-sup-0013-TableS8..docx]

| **Supplementary Table 7. Secondary outcome amputation and ulcer measures and other secondary outcome measures including between-group difference reported in studies investigating the effectiveness of nutrition interventions for individuals with diabetes-related foot ulceration.** | | | | | | |
| --- | --- | --- | --- | --- | --- | --- |
| **Reference, country** | **Amputation rate** | **Amputation rate between group difference at follow-up between group difference at follow-up** | **Surgical Intervention** | **Surgical Intervention between group difference at follow-up** | **Recurrence of ulcer** | **Recurrence of ulcer between group difference at follow-up** |
| **Single nutrient supplement studies (Reported outcomes n=2)** | | | | | | |
| Bashmakov 2014,  Egypt, Trans-resveratrol | NR | NR | NR | NR | NR | NR |
| Gunton 2021,  Australia, Vitamin C | **Amputation (n)**  Control:2 (22.22%)  Intervention: 0 (0.00%) | NS difference (p=0.1849, 95%CI -0.05, 0.49)* | NR | NR | NR | NR |
| Halschou-Jensen 2021,  Denmark, Vitamin D | **Control**  Lower leg amputation (n): 2 (9.09%)  Metatarsal amputation (n): 5 (22.72%)  Toe amputation (n): 0 (0.00%)  **Intervention**  Lower leg amputation (n): 0 (0.00%)  Metatarsal amputation (n): 2 (8.70%)  Toe amputation (n): 2 (8.70%) | **Lower leg amputation:** NS difference (p=0.1412, 95%CI -0.03, 0.21)*  **Metatarsal amputation:** NS difference (p=0.1946, 95%CI -0.07, 0.35)*  **Toe amputation:** NS difference (p=0.1570, 95%CI -0.20, 0.03)* | **Extensive toe debridement (n)**  Control: 2 (9.09%)  Intervention: 1 (4.35%) | NS difference (p=0.5240, 95%CI -0.10, 0.19) | NR | NR |
| Kamble 2020,  India, Vitamin D | NR | NR | NR | NR | NR | NR |
| Mozaffari-Khosravi 2016,  Iran, Vitamin D | NR | NR | NR | NR | NR | NR |
| Rangabashyam 2020,  India, Vitamin D | NR | NR | NR | NR | NR | NR |
| Razzaghi 2017,  Iran, Vitamin D | NR | NR | NR | NR | NR | NR |
| Jain 2012,  India, Vitamin E | NR | NR | NR | NR | NR | NR |
| Mohseni 2018,  Iran, Probiotic | NR | NR | NR | NR | NR | NR |
| Mokhtari 2020,  Iran, Nanocurcumin | NR | NR | NR | NR | NR | NR |
| Momen-Heravi 2017,  Iran, Zinc | NR | NR | NR | NR | NR | NR |
| Razzaghi 2018,  Iran, Magnesium | NR | NR | NR | NR | NR | NR |
| Soleimani 2017,  Iran, Omega-3 | NR | NR | NR | NR | NR | NR |
| **Multi-nutrient supplement studies (Reported outcomes n=3)** | | | | | | |
| Afzali 2019,  Iran, Mg and vitamin E | NR | NR | NR | NR | NR | NR |
| Bosede 2012,  Nigeria, Vitamin E, C and selenium | NR | NR | NR | NR | NR | NR |
| Yarahmadi 2021,  Iran, Vitamin E and C | NR | NR | NR | NR | NR | NR |
| Das 2022,  India, Amino acids | NR | NR | NR | NR | NR | NR |
| Armstrong 2014,  USA, Europe and Taiwan, Arginine, glutamine and HMB | **Amputation n/N%**  Control: 5/141 (3.5)  Intervention: 3/129 (2.3) | NS difference (p=0.552) | NR | NR | **Recurrence n/N(%)**  Control: 12/141 (8.5)  Intervention: 11/129(8.5)  **Additional lesions n/N(%)**  Control: 40/141 (28.4)  Intervention: 38/129 (29.5)  **Wound infection n/N(%)**  Control: 15/141 (10.6)  Intervention: 21/129 (16.3) | **Recurrence**  NS difference (p=0.996)  **Additional lesions**  NS difference (p=0.844)  **Wound infection**  NS difference (p=0.173) |
| Eneroth 2004,  Sweden, Fortimel | **Amputation within 6 months (n)**  Control:0/23 (0%)  Intervention: 2/17 (11.76%) | NS difference (p=0.0915, 95%CI -0.27, 0.04)* | NR | NR | NR | NR |
| Yanes-Quesada  2021,  Cuba, Diamel | NR | NR | NR | NR | **Recurrence n(%)**  Control: 8/44 (18)*  Intervention: 3/46 (7)* | NS difference (p=0.1132, 95%CI -0.03, 0.25)* |
| **Nutrition education (Reported outcomes n=0)** | | | | | | |
| Basiri 2020,  USA, Dietitian and Boost Glucose Control supplement | NR | NR | NR | NR | NR | NR |
| Sung 2021,  Australia, MDT | NR | NR | NR | NR | NR | NR |
| Yang 2023,  China, Early nurse-led nutrition intervention | NR | NR | NR | NR | NR | NR |
| Abbreviations  NS = Non-significant  NR = Not Reported  RD = Registered Dietitian  TG = Triglycerides  VLDL = Very Low-Density Lipoprotein  TC = Total Cholesterol  LDL = Low-Density Lipoprotein  HDL = High-Density Lipoprotein  SD = Standard Deviation  CI = Confidence Intervals   1. Values are adjusted for baseline values of each biochemical variable, age and baseline BMI. 2. Values are adjusted for baseline values of each biochemical variable.   ^The worst result was selected in order to not misrepresent the data, as different tables reported different results. Nil email found for authors on the published paper.  *= between group differences calculated from individual group summary statistics  *Note:* results non-adjusted unless specified  *Note:* A calculated conversion completed for studies that report HbA1c in mg/dl as per journal author guidelines | | | | | | |
