# Supplementary material for: Effectiveness of dietary interventions in individuals with diabetes for preventing and healing chronic wounds; a systematic review with meta‐analysis
Source: Diabet Med. 2025 Jul 9;42(9):e70100. doi: 10.1111/dme.70100 (PMC12352720; doi:10.1111/dme.70100)
Supplement: Supplementary file 1 — Data S1. [file DME-42-e70100-s001.zip › dme70100-sup-0014-TableS9..docx]

| **Supplementary Table 9. Food sources of key nutrients for wound healing** | | |
| --- | --- | --- |
| **Nutrient** | **Wound healing properties** | **Food sources** |
| Magnesium | Role in inflammation and oxidative stress^1^ | Green vegetables, legumes, peas, and nuts^2^ |
| Zinc | White blood cell formation, collagen synthesis, fibroblast proliferation in wound healing, and within the immune response^3^ | Meat, fish, and eggs^3^ |
| Omega 3 ((Eicosapentaenoic acid (EPA) and docosahexaenoic acid (DHA)) | Support membrane fluidity/signalling, apoptosis modulator, and intracellular signalling^3^ | Oily fish including mackerel, sardines, salmon and tuna^4^ |
| Probiotics | Anti-inflammatory and supports immune system^5^ | Yoghurt, cheese^5,6^ |
| Vitamin C | Antioxidant, collagen synthesis and immunomodulation involved in proliferative and remodelling phases of wound healing^3^ | Citrus fruits, tomatoes, potatoes and broccoli^3^ |
| Vitamin D | Anti-inflammatory effects and supports structural integrity in wound healing^3,7^ | Fatty fish, fortified milk and breakfast cereals, sunlight^3^ |
| Vitamin E | Antioxidant, quenches free radicals, collagen synthesis and helps membrane integrity^3^ | Wheat germ, seeds, nuts, vegetable oils, green leafy vegetables and fortified cereals^3^ |
| References  1. Razzaghi R, Pidar F, Momen-Heravi M, Bahmani F, Akbari H, Asemi Z. Magnesium Supplementation and the Effects on Wound Healing and Metabolic Status in Patients with Diabetic Foot Ulcer: a Randomized, Double-Blind, Placebo-Controlled Trial. *Biol Trace Elem Res*. Feb 2018;181(2):207-215. doi:10.1007/s12011-017-1056-5  2. National Health and Medical Research Council. Magnesium - Nutrient Reference Values for Australia and New Zealand. NHMRC. <https://www.eatforhealth.gov.au/nutrient-reference-values/nutrients/magnesium>  3. Armstrong D, Mills J, Molina M, Molnar J. Nutrition Interventions in Adults with Diabetic Foot Ulcers Expert Consensus and Guidance. American Limb Preservation Society <https://eguideline.guidelinecentral.com/i/1428995-nutrition-in-dfu-guidelines-advisory-pocket-guide/0>?  4. National Health and Medical Research Council. Fats: Total fat & fatty acids - Nutrient Reference Values for Australia and New Zealand. NHMRC. <https://www.eatforhealth.gov.au/nutrient-reference-values/nutrients/fats-total-fat-fatty-acids>  5. Mohseni S, Bayani M, Bahmani F, et al. The beneficial effects of probiotic administration on wound healing and metabolic status in patients with diabetic foot ulcer: a randomized, double-blind, placebo-controlled trial. *Diabetes/Metabolism Research and Reviews*. 2017;(no pagination)doi:10.1002/dmrr.2970  6. Bodke H, Jogdand S. Role of Probiotics in Human Health. *Cureus*. Nov 2022;14(11):e31313. doi:10.7759/cureus.31313  7. Apergi K, Dimosthenopoulos C, Papanas N. The Role of Nutrients and Diet Characteristics in the Management of Diabetic Foot Ulcers: A Systematic Review. *Int J Low Extrem Wounds*. Feb 3 2023:15347346231153531. doi:10.1177/15347346231153531 | | |
